# Metformin and epigenetic age in non-diabetic older people with HIV in Madrid (METFORAGING): a double-blind, randomised, placebo-controlled, pilot trial

**DOI:** 10.1016/j.eclinm.2026.103874

**Published:** 2026-04-18

**Authors:** Cristina Marcelo-Calvo, Andrés Esteban-Cantos, Francisco Jurado, Rocío Montejano, Javier Rodríguez-Centeno, Lucía Gutiérrez-García, Alejandro de Gea-Grela, Patricia Martínez-Martín, Alejandro Díez Vidal, Rosa de Miguel, Carlos M. Oñoro-López, Juan Carlos González, Luz Martín-Carbonero, José Ignacio Bernardino, Rocío Menéndez Colino, Noemí González Pérez de Villar, Berta Rodés, José Ramón Arribas

**Affiliations:** a Department of Internal Medicine, HIV Unit, La Paz University Hospital, Madrid, Spain; b Hospital La Paz Institute for Health Research (IdiPAZ), Madrid, Spain; c CIBER of Infectious Diseases (CIBERINFEC), Madrid, Spain; d Genetic and Molecular Epidemiology Group, Spanish National Cancer Research Centre (CNIO), Madrid, Spain; e Department of Geriatrics, La Paz University Hospital, Madrid, Spain; f Department of Endocrinology and Nutrition, La Paz University Hospital, Madrid, Spain

**Keywords:** HIV, Aging, Epigenetic clocks, Metformin, Double-blind, Randomised, Pilot trial

## Abstract

**Background:**

Metformin is increasingly studied as a potential geroprotective agent in the general population. We aimed to test the efficacy and safety of metformin to improve epigenetic age in older, well-controlled, non-diabetic people living with HIV.

**Methods:**

METFORAGING was a single-centre, double-blind, randomised, parallel-group, placebo-controlled pilot trial. Non-diabetic participants living with HIV who were aged 50 years or older, virologically suppressed, on a stable antiretroviral regimen with undetectable viral load for at least 12 months, and had CD4^+^ T-cell counts >500 cells/μL were recruited from the HIV clinic at La Paz University Hospital (Madrid, Spain). Participants were randomly assigned (1:1) to receive 850 mg oral metformin or matching placebo twice a day for 96 weeks. Participants, investigators, and outcome assessors were masked to treatment allocation. Study visits were conducted at baseline and at weeks 4, 8, 24, 48, 72, and 96. Adherence was evaluated at each visit through pill count and self-report. At baseline and week 96, whole-blood samples were used to calculate biological age across 11 epigenetic biomarkers: first-generation epigenetic clocks (Horvath’s clock and Hannum’s clock), second-generation epigenetic clocks (PhenoAge and GrimAge V2), principal component-derived epigenetic clocks (PC-Horvath, PC-Hannum, PC-PhenoAge and PC-GrimAge), a third-generation clock (DunedinPACE), and the DNA methylation-based estimator of blood telomere length (DNAmTL). The primary outcome was the adjusted between-group difference in epigenetic age acceleration (EAA) change measured by the PhenoAge clock at week 96 in the per-protocol population. Analyses were stratified by age, sex, baseline CD4 count, smoking status, statin treatment, and route of HIV transmission. The trial was registered with EudraCT, 2021-003299-15.

**Findings:**

Between March 2, and Oct 2, 2022, 55 individuals were screened, and 40 were randomly assigned to metformin (n = 19) or placebo (n = 21). Enrolment was closed at 40 participants because of slow recruitment, below the pragmatic target of 60 outlined in the study protocol. Median age was 56.4 years (IQR 53.0–60.8), 12 (30%) were female, and 35 (87.5%) self-identified as White. Mean adherence by pill count was 97.5% in both groups. 35 (87.5%) of 40 participants continued treatment to 96 weeks (n = 17 in the metformin group and n = 18 in the placebo group; per-protocol population). At week 96, the adjusted between-group difference (metformin vs. placebo) for PhenoAge EAA was −1.02 years (95% confidence interval [CI] −5.30 to 3.26; p = 0.627). 48 adverse events occurred in 16 (84%) of 19 participants who received metformin and 48 adverse events occurred in 19 (90%) of 21 participants who received placebo. In the metformin group, no serious adverse events were attributed to the medication and no deaths or hospitalisation occurred.

**Interpretation:**

Although no significant difference was noted in the primary outcome between groups, these preliminary findings support the feasibility of geroscience-trials in this population and support further investigation of metformin in larger, adequately powered studies to determine whether metformin can modify biological ageing in people with HIV.

**Funding:**

Fondo de Investigaciones Sanitarias, Instituto de Salud Carlos III, and the European Union.

**Research in context**

**Evidence before this study**

People living with HIV exhibit higher epigenetic age acceleration (EAA) than the general population, even after long-term virological suppression. Metformin is increasingly studied as a potential geroprotective agent in the general population. We searched PubMed on October 2025 using the terms (“metformin”) AND (“HIV” OR “people living with HIV”) AND (“epigenetic clocks” OR “DNA methylation” OR “biological ageing”), with no date or language restrictions. While growing evidence links HIV with accentuated biological ageing, only limited data exist on geroprotective interventions in this population. Prior studies suggest metformin may reduce T-cell exhaustion or affect epigenetic ageing in selected immune cell types, but no randomised trial has assessed its impact on whole-blood DNA methylation clocks in non-diabetic persons with HIV.

**Added value of this study**

This is the first randomised, double-blind, placebo-controlled trial to evaluate the long-term safety and biological effects of metformin on ageing biomarkers in non-diabetic, virologically suppressed people with HIV. Although no statistically significant between-group differences were observed, metformin was well tolerated over 96 weeks, and the direction of change across epigenetic clocks and DNA methylation–derived telomere length favoured metformin.

**Implications of all the available evidence**

Metformin appears safe in older individuals without diabetes who are living with HIV and may influence biological ageing markers. These preliminary findings support the feasibility of geroscience-trials in this population and justify further investigation of metformin in larger studies.

## Introduction

In people living with HIV (PLWH), accentuated ageing occurs despite effective antiretroviral therapy (ART), resulting in a higher incidence of age-related diseases compared with the general population.^1^ Accordingly, individuals aged 50 years or older are commonly classified as older adults in HIV research and clinical practice.^2^ Proposed drivers for this include persistent immune activation, chronic inflammation, and immunosenescence, although the underlying mechanisms remain incompletely understood.^1^^,^^3^

In recent years, epigenetic clocks—based on DNA methylation patterns—have emerged as promising tools for estimating biological age. These clocks correlate strongly with chronological age and predict morbidity, mortality, and functional decline.^4^ First-generation epigenetic clocks (such as the Horvath and Hannum clocks) were designed to estimate chronological age, while second-generation models, such as PhenoAge and GrimAge—trained on clinical and mortality outcomes—and their principal component (PC) derivatives (PC-PhenoAge, PC-GrimAge, PC-Hannum, and PC-Horvath), which apply dimensionality reduction to improve measurement reliability, improve prediction of age-related comorbidities and all-cause mortality.^5^ More recently, third-generation clocks like DunedinPACE, which estimate the pace of biological ageing, have shown value in predicting functional and cognitive decline as well as mortality risk.^6^ PLWH exhibit higher epigenetic age acceleration (EAA) than the general population, even after long-term virological suppression.^7^ Whether this acceleration can be reversed through targeted interventions remains unknown.

Among pharmacological interventions to delay ageing, metformin—a widely used antidiabetic drug— has emerged as a potential geroprotective agent. Beyond its glucose-lowering effects, metformin modulates multiple pathways implicated in ageing, including AMPK activation, mTOR inhibition, mitochondrial regulation, autophagy, and inflammation.^8^ Observational studies in people with type 2 diabetes have associated metformin with reduced cardiovascular events, cancer incidence, frailty, and all-cause mortality.^9^^,^^10^ These findings have prompted the development of large-scale clinical trials, most notably the TAME (Targeting Aging with Metformin) study, designed to evaluate metformin’s effects on ageing-related outcomes in non-diabetic older adults.^11^

Preliminary studies suggest that metformin may reduce EAA measured by DNA methylation clocks in healthy individuals and animal models.^12^^,^^13^ In the context of HIV, short-term studies in well-controlled, non-diabetic PLWH suggest that metformin may attenuate immune exhaustion and residual reservoir transcription, and a small 24-week trial reported reductions in monocyte epigenetic age (PC-PhenoAge and PC-GrimAge) with no change in CD8^+^ T cells.^14^^,^^15^ Whether such cell-type–specific signals are detectable in whole blood, persist with longer-term treatment, and are demonstrable in a randomised, placebo-controlled setting remains unknown.

To address this gap, we aimed to conduct a randomised, double-blind, placebo-controlled, phase 2 pilot trial to evaluate the long-term safety and explore potential effects of metformin on biological ageing in PLWH. Using validated DNA methylation–based biomarkers, the study is designed to provide preliminary data on metformin’s capacity to modulate epigenetic ageing in this population and to inform the design of future, larger trials.

## Methods

### Study design and ethics

METFORAGING is a double-blind, randomised, parallel-group, placebo-controlled, pilot trial. Participants were recruited from the HIV clinic at La Paz University Hospital (Madrid, Spain). Enrolment began on March 2, 2022. Participants are being followed through week 144, with the intervention period ending at week 96 and subsequent follow-up continuing without study medication. The present report describes results through the primary efficacy endpoint at week 96. Potentially eligible participants underwent a screening assessment in which initial eligibility criteria were confirmed. The trial is registered in August 5, 2021 in the EudraCT database (2021-003299-15) where the protocol is available. The study protocol is also available within the Supplementary Materials. The trial sponsor is La Paz University Hospital Research Foundation.

All participants provided written informed consent. The trial was approved by the Ethics Committee of La Paz University Hospital (approval number HULP-5971). It is being conducted in accordance with the principles of the Declaration of Helsinki and the International Council for Harmonisation Good Clinical Practice (ICH-GCP).

### Participants

Eligible participants were PLWH aged 50 years or older, on stable ART for at least three months before screening, with plasma HIV-RNA < 50 copies/mL for at least one year prior to study entry, and a CD4^+^ T-cell count >500 cells/μL. Additional inclusion criteria were HOMA-IR ≤ 2.6, body mass index (BMI) < 30 kg/m^2^, and normal vitamin B12 levels (>211 pg/mL). Postmenopausal status was required for women, defined as 12 months or more of amenorrhoea or missed at least one menstrual period in the previous year. Exclusion criteria included diabetes or prediabetes (fasting glucose 100–125 mg/dL, 2-h glucose after a 75 g oral glucose tolerance test 140–199 mg/dL, or glycated haemoglobin 5.7–6.4%)^16^; estimated glomerular filtration rate (eGFR) less than 60 mL/min; chronic hepatitis B, active hepatitis C or progressive liver disease; liver enzyme abnormalities (ALT > 5 times the upper limit of normal [ULN], or ALT > 3 × ULN with bilirubin > 1.5 × ULN); significant comorbidities including stroke, cancer, heart failure, myocardial infarction, cognitive impairment, or previous lactic acidosis; haemodynamic instability; alcohol abuse (≥15 drinks/week for men or ≥8 drinks/week for women); and contraindicated medications with metformin and hypersensitivity or intolerance to metformin. Sex assigned at birth was collected during screening.

### Randomisation and masking

Participants were randomly assigned (1:1) to either metformin or matched placebo. Randomisation, stratified by age, was done by a statistician using a computer-generated sequence (SAS 9.0 for Windows). Allocation was centrally managed through the REDCap electronic data system. Study medication was prepared in identical bottles by GalenicumVitae (Barcelona, Spain) and dispensed by trial investigators with no indication of randomisation assignment on the label. Participants, investigators, and outcome assessors were masked to treatment allocation. Unblinding was allowed only in case of serious adverse events, in accordance with regulatory requirements and sponsor policy.

### Procedures

Participants received oral metformin 850 mg (GalenicumVitae, Barcelona, Spain) twice daily or matched placebo for 96 weeks. To improve gastrointestinal tolerance, dosing began at 850 mg once daily and was increased to twice daily after four weeks. For participants receiving dolutegravir, the dose remained at 850 mg once daily due to a known interaction via organic cation transporter 2 (OCT2), which increases metformin exposure and reduces clearance.^17^ The dose was based on prior ageing-related studies.

Study visits were conducted at baseline, week 4, week 8 (if dose escalation occurred), and weeks 24, 48, 72, and 96. Adherence was evaluated at each visit through pill count and self-report, and calculated as follows: (the number of tablets taken/the number of tablets expected to be taken by the final study visit) × 100. Serum GDF15 levels, a biomarker of metformin exposure, were measured at baseline and week 96.^18^ Blood samples for complete blood count, CD4^+^ T-cell count, HIV viral load, serum creatinine, liver enzymes, and fasting glucose were obtained at each visit.

Adverse events were collected at each study visit by clinical evaluation and laboratory tests. Events were recorded if considered possibly, probably, or definitely related to the study drug, and graded according to the Division of AIDS Table for Grading the Severity of Adult and Paediatric Adverse Events (version 2.1). Adverse reactions were defined as adverse events which were possibly, probably, or definitely causally related to metformin.

#### DNA methylation analysis

We analysed DNA methylation patterns in whole blood samples. Genomic DNA was extracted and subjected to sodium-bisulfite conversion using the MagPurix Blood DNA Extraction Kit 200 (Zinexts Life Science; New Taipei, Taiwan) and the EZ DNA Methylation Kit (Zymo Research; Irvine, CA, USA), respectively. DNA methylation profiling was performed using the Infinium MethylationEPIC v2.0 kit (Illumina; San Diego, CA, USA) according to the manufacturer’s instructions at the same core facility (Genomics Service of Life & Brain; Bonn, Germany). To minimise batch effects, samples were randomly distributed across plates and chips, and baseline and follow-up samples from the same participants were placed on the same chips. All samples were required to meet Illumina’s quality control standards.

#### Epigenetic biomarkers of ageing

At baseline and week 96, we calculated first-generation epigenetic clocks (Horvath’s clock^19^ and Hannum’s clock^20^), second-generation epigenetic clocks (PhenoAge^21^ and GrimAge V2^5^), and the DNA methylation-based estimator of blood telomere length (DNAmTL)^22^ using Horvath’s DNA Methylation Age calculator software.^19^ The third-generation clock DunedinPACE was generated using the code provided by Belsky et al. (https://github.com/danbelsky/DunedinPACE).^6^ We also evaluated the principal component (PC)-based versions of Horvath’s, Hannum’s, PhenoAge, and GrimAge epigenetic clocks, as well as DNAmTL, as previously reported.^22^^,^^23^

For first-generation and second-generation epigenetic clocks (original and PC versions), epigenetic age acceleration (EAA) was defined as the residual from regressing epigenetic age on chronological age (positive values indicate older-than-expected epigenetic age). For DNAmTL, age-adjusted values (DNAmTL-AA) were residuals from regressing DNAmTL on chronological age (positive values indicate longer-than-expected telomere length). DunedinPACE estimates the pace of biological ageing, with a value of 1.0 representing the reference point equivalent to one biological year per chronological year.

#### Frailty assessment

Frailty was assessed at baseline and week 96 using the Fried frailty phenotype, FRAIL scale, Frail-VIG index, and the Short Physical Performance Battery (SPPB). The Fried phenotype includes five components:^24^ self-reported unintentional weight loss and exhaustion, physical activity (short version of the Minnesota Leisure Time Physical Activity Questionnaire, adjusted by sex), gait speed over 4-m (adjusted for sex and height), and dominant handgrip strength (adjusted for sex and BMI), measured with a Jamar hydraulic dynamometer (Sammons Preston, Bolingbrook, IL, USA). Participants meeting three or more criteria were classified as frail; those with one or two criteria as pre-frail; and those meeting none as robust.

The FRAIL scale includes five self-reported items: fatigue, difficulty climbing ten stairs, difficulty walking 100 m, presence of ≥5 chronic conditions, and unintentional weight loss ≥5% in the past year. Each positive item scores one point, categorised as robust (0), pre-frail (1–2), or frail (≥3).

The Frail-VIG index evaluates deficits across functional, cognitive, nutritional, and social domains. The score ranges from 0 to 1, with higher scores indicating greater frailty. Participants were categorised as fit (<0.2), mildly frail (0.2–0.35), moderately frail (0.36–0.5), or severely frail (>0.5). The SPPB included three timed tasks: standing balance, gait speed, and chair stands, with scores ranging from 0 to 12.^25^

### Outcomes

The primary outcome was the adjusted between-group difference in the change of EAA at week 96, measured by the PhenoAge epigenetic clock in the per-protocol population.

Secondary outcomes were between-group and within-group differences in the change of EAA at week 96 assessed by additional clocks: Horvath, Hannum, GrimAge V2, DunedinPACE, and principal component-based versions of these clocks. Other secondary outcomes were change in DNAmTL at week 96; change in frailty status at week 96 using multiple validated instruments (Fried phenotype, FRAIL scale, Frail-VIG index, and SPPB), and safety outcomes, including adverse events and laboratory parameters at all visits. Adverse events were classified by severity and causality as previously described.

Additionally, body weight and anthropometric parameters, together with inflammatory, metabolic, and immunophenotypic changes, were assessed as secondary outcomes and will be reported elsewhere.

### Statistical analysis

As this was an exploratory pilot study, no formal sample size calculation was undertaken; the protocol specified a pragmatic target of 60 participants to be randomised. All analyses were interpreted as hypothesis-generating. Participant characteristics in the intention-to-treat population were reported as absolute and relative frequencies for categorical variables, and as medians with interquartile ranges (IQRs) for continuous variables. Efficacy analyses were conducted in the per-protocol population (all randomised participants who continued treatment to 96 weeks), whereas safety analyses used the safety population (all participants who received at least one dose of study medication).

All primary and secondary analyses were prespecified in the study protocol. The primary analysis was conducted using a prespecified linear regression-based analysis of covariance (ANCOVA) model. The treatment effect on PhenoAge EAA at week 96 was modelled as the dependent variable, with treatment group as the main independent variable and the baseline value of that biomarker, age, sex, baseline CD4 count, smoking status, statin treatment, and route of HIV transmission as prespecified covariates. This approach is statistically appropriate for randomised clinical trials with two time points, as adjusting for baseline values accounts for between-subject variability without requiring subject-specific random effects. The “lm” function from the “stats” package (R version 4.2.2) was used for this analysis. Results were presented as adjusted mean differences (95% CI). Secondary epigenetic ageing outcomes were analysed analogously using the same prespecified covariates. Model assumptions were evaluated, and because the distribution of variables deviated from normality, prespecified non-parametric two-sided Wilcoxon signed-rank tests were performed to assess within-group differences between baseline and week-96 EAA; the “wilcox.test” function from the “stats” package was used. In a post-hoc analysis added during peer review, Spearman correlations were calculated to evaluate the association between changes in serum GDF-15 levels and changes in each epigenetic ageing biomarker from baseline to week 96. No sensitivity analyses were performed, as the limited sample size (n = 35) precluded such analyses without substantially compromising statistical reliability. No other post-hoc analyses were conducted.

Safety outcomes were evaluated using descriptive statistics. Categorical frailty phenotypes (Fried phenotype, FRAIL scale, and Frail-VIG index) were summarised by category and compared between groups at week 96 using Fisher’s exact test. The SPPB frailty scale was analysed as a continuous variable and reported as the median with range. Model assumptions were assessed by visual inspection of residuals.

All analyses were conducted in R (version 4.2.2). We report descriptive and inferential statistics with two-sided p-values and 95% confidence intervals, without adjustment for multiple comparisons.

### Role of the funding source

The funder (Instituto de Salud Carlos III and European Union) and sponsor (La Paz University Hospital Research Foundation) had no role in the collection, data analysis, data interpretation, writing of the report, or the decision to submit the results for publication.

## Results

**Fig. 1:**
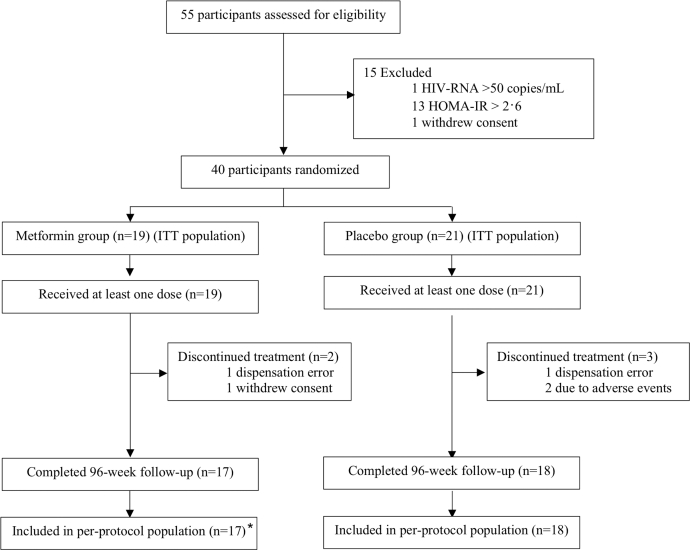
**Trial profile.** ∗All randomised participants who continued treatment to 96 weeks.

Between March 2 and Oct 2, 2022, 55 individuals were screened; 15 were excluded (14 ineligible, one withdrew consent). 40 participants were randomly assigned to metformin (n = 19) or placebo (n = 21) (Fig. 1). Enrolment was closed at 40 because of slow recruitment in this single-centre pilot, below the protocol’s pragmatic target of 60. Five participants discontinued treatment before week 96: two in the metformin group (one withdrew consent and one due to a dispensing error) and three in the placebo group (one due to a dispensing error, one drug-related adverse event, and one non–drug-related adverse event). 35 participants (metformin 17, placebo 18) continued their allocated treatment through week 96 and comprised the per-protocol population. Median age was 56.4 years (interquartile range [IQR] 53.0–60.8); 12 (30%) were female and 35 (87.5%) were white (Table 1).

**Table 1:** Baseline characteristics of the intention-to-treat-population.

	Metformin group (n = 19)	Placebo group (n = 21)
Chronological age (years)	56.5 (52.1–62.4)	56.2 (53.7–59.7)
Sex at birth		
Female	7 (36.8%)	5 (23.8%)
Male	12 (63.2%)	16 (76.2%)
Ethnicity		
White	17 (89.5%)	18 (85.7%)
Latin	2 (10.5%)	3 (14.3%)
Current smokers	7 (36.8%)	4 (19.0%)
Mode of HIV transmission		
Men who have sex with men	10 (52.6%)	10 (47.6%)
Heterosexual	6 (31.6%)	2 (9.5%)
Injection drug use	3 (15.8%)	9 (42.9%)
Nadir CD4 (cells/mm^3^)	311 (91–350)	304 (200–428)
CD4 count (cells/mm^3^)	777 (630.5–971)	729 (644–887)
Ratio CD4/CD8	1.1 (0.9–2.1)	1.2 (1–1.4)
Years since HIV diagnosis	22.5 (18.2–25.0)	26.6 (20.0–31.8)
BMI (kg/m^2^)	24.3 (22.9–25.8)	24.5 (22.0–26.0)
HOMA-IR	1.6 (1.3–2.3)	1.4 (1.1–2.5)
Fasting glucose (mg/dL)	88 (83.5–94.5)	83 (79–90)
HbA1c (%)	5.4 (5.3–5.6)	5.5 (5.3–5.7)
Hypertension (%)	6 (31.6%)	7 (33.3%)
Dyslipidemia (%)	9 (47.4%)	6 (28.6%)
ASCVD risk	5% (3–4.9)	5.6% (5.1–6)
Osteoporosis (%)	2 (10.5%)	1 (4.8%)
Antihypertensive treatment (%)	6 (31.6%)	7 (33.3%)
Statin therapy (%)	8 (42.1%)	3 (14.3)
Cytomegalovirus IgG seropositive	18 (94.7%)	20 (95.2%)
Antiretroviral treatment with dolutegravir (%)	11 (57.9%)	16 (76.2%)

Data are presented as n (%) or median (IQR).

BMI = body mass index. HOMA-IR = Homeostatic Model Assessment for Insulin Resistance. HbA1c = glycated haemoglobin. ASCVD = Atherosclerotic Cardiovascular Disease.

Mean adherence by pill count was 97.5% in both groups. In the metformin arm, mean adherence among participants receiving and not receiving dolutegravir was 97.7% and 96.7%, respectively. The increase in serum GDF15 levels from baseline to week 96 was significantly greater in the metformin group compared with the placebo group (median change: 486.9 and 104.4 pg/mL respectively; p < 0.001; Supplementary Fig. S1).

No significant between-group difference was noted for the primary outcome. At week 96, the adjusted between-group difference (metformin vs. placebo) for PhenoAge EAA was −1.02 years (95% CI −5.30 to 3.26; p = 0.627).

**Fig. 2:**
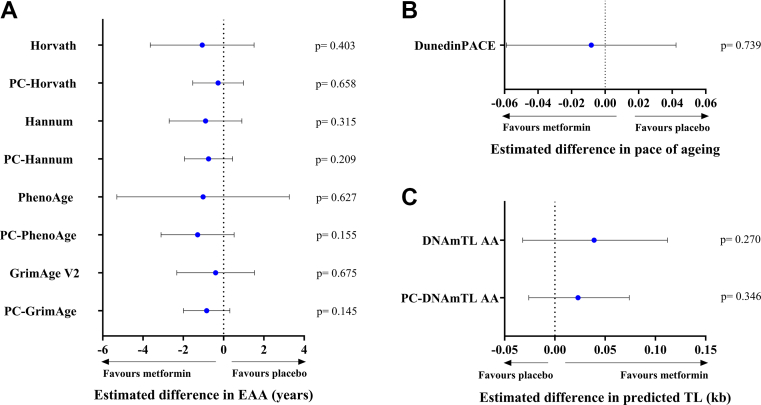
**Adjusted between-group differences in epigenetic ageing biomarkers at week 96 (metformin—placebo).** Forest plots representing between-group differences at week 96 in A) epigenetic age acceleration (EAA) as measured by first- and second-generation epigenetic clocks (original and principal component (PC)-derived versions); B) DunedinPACE; and C) original and PC-derived version of the DNA methylation-based estimator of telomere length (DNAmTL). Points show adjusted mean differences; horizontal bars indicate 95% confidence intervals (CIs). Estimates were obtained by analysis of covariance (ANCOVA) with week-96 biomarker value as the dependent variable, adjusting for baseline value of the same biomarker, age, sex, baseline CD4 count, smoking status, statin treatment, and HIV transmission route (per-protocol: metformin n = 17; placebo n = 18). Negative values for EAA and DunedinPACE indicate lower (favourable) values with metformin; positive values for DNAmTL indicate longer telomeres (favourable). Two-sided p-values from ANCOVA are displayed adjacent to each point estimate.

Findings were consistent across the other clocks in the fully adjusted model. For the original clocks, the adjusted differences in EAA were: Horvath, −1.06 years (95% CI −3.64 to 1.51; p = 0.403); Hannum, −0.89 years (95% CI −2.70 to 0.90; p = 0.315); and GrimAge V2, −0.40 years (95% CI −2.32 to 1.53; p = 0.675). For the PC-derived clocks, estimates were likewise non-significant: PC-PhenoAge, −1.29 years (95% CI −3.10 to 0.52; p = 0.155); PC-GrimAge, −0.84 years (95% CI −1.99 to 0.31; p = 0.145); PC-Hannum, −0.74 years (95% CI −1.94 to 0.44; p = 0.209); and PC-Horvath, −0.27 years (95% CI −1.53 to 0.98; p = 0.658). DunedinPACE showed an adjusted difference of −0.008 (95% CI −0.058 to 0.042; p = 0.739). Across all nine epigenetic clocks analysed, the point estimates favoured metformin but no significant differences were seen between groups (Fig. 2A and B). For telomere-related measures, the adjusted differences were 0.039 (95% CI −0.033 to 0.112; p = 0.270) for DNAmTL and 0.023 (95% CI −0.026 to 0.073; p = 0.346) for PC-DNAmTL (Fig. 2C). We also examined associations between changes in GDF-15 and changes in epigenetic biomarkers; no correlations were observed at week 96 (Supplementary Table S1).

Intragroup analyses from baseline to week 96 revealed no statistically significant changes in EAA or DNAmTL in the metformin group (Supplementary Table S2). A similar pattern was observed in the placebo group, except for a statistically significant decrease in DNAmTL (p = 0.038). This trend was not observed in the PC-based version of DNAmTL. Across both groups, variability in EAA measures was high, and no consistent within-group changes were observed across first-, second-, or third-generation clocks.

No significant differences in frailty status were observed at baseline between groups (Supplementary Table S3). According to the Fried phenotype, the proportion of robust individuals decreased from 94.1% at baseline to 58.8% at week 96 in the metformin group, and from 88.9% to 77.8% in the placebo group, but these changes were not statistically significant (p = 0.355). No differences were observed in FRAIL status, and all participants remained classified as “fit” according to the Frail-VIG index at week 96. SPPB scores remained stable with a median of 12 in both groups.

A total of 48 adverse events were reported in 16 (84%) of 19 participants in the metformin group, and 48 events occurred in 19 (90%) of 21 participants in the placebo group (Table 2). No deaths, hospitalisations, or cases of lactic acidosis were reported. One grade 3 adverse event occurred in the metformin group—an episode of acute kidney injury deemed unrelated to the study drug—which required temporary dose reduction. In the placebo group, one participant discontinued treatment due to drug-related flatulence, and one unrelated serious adverse event was reported. In the metformin group, no serious adverse events were attributed to the medication.

**Table 2:** Adverse events.

	Metformin group (n = 19)	Placebo group (n = 21)
Participants with at least one adverse event	16	19
Total number of adverse events	48	48
Any grade ≥3 adverse events	1	0
Drug-related	0	0
Any adverse event leading to withdrawal	0	1
Drug-related	0	1
Serious adverse events	0	1
Drug-related	0	0
Most common drug-related adverse events		
Diarrhoea	4	0
Flatulence	1	2
Gastroesophageal reflux	3	1
Number of hospital admissions	0	0
Number of deaths	0	0

Data are presented as n (%). Shown are all adverse events that occurred during the 96-week treatment period. Adverse events were classified by investigators as possibly, probably, or definitely related to study treatment, and graded according to the Division of AIDS Table for Grading the Severity of Adult and Paediatric Adverse Events (version 2.1).

Drug-related adverse events were generally mild and self-limited. Diarrhoea was the most common event in the metformin group, affecting four participants (21%), and typically resolved within two–three days. One participant required dose adjustment to 850 mg once daily due to persistent symptoms. Other reported drug-related events included gastroesophageal reflux and flatulence (Table 2). Tolerability was comparable in participants receiving dolutegravir (metformin 850 mg once daily) and those not receiving dolutegravir (850 mg twice daily): diarrhoea 2/11 vs. 2/8, flatulence 1/11 vs. 0/8, gastroesophageal reflux 2/11 vs. 1/8; there were no drug-related serious adverse events or withdrawals, and a single dose reduction occurred in the twice-daily subgroup. A full listing of adverse events is provided in the appendix (Supplementary Table S4).

No significant differences were observed between groups in laboratory safety parameters at week 96. Mean (SD) change in CD4^+^ T-cell count was −58 (140.7) cells/mm^3^ in the metformin group and 14 (142.6) cells/mm^3^ in the placebo group (p = 0.144). All participants maintained virological suppression throughout the study. Mean (SD) change in serum creatinine was 0.013 (0.086) mg/mL in the metformin group and 0.021 (0.057) mg/mL in the placebo group (p = 0.759). Changes in liver enzymes were comparable between groups: ALT, −2.06 (7.54) IU/L in the metformin group vs. −1.11 (6.77) IU/L in the placebo group (p = 0.698); and AST, −14.53 (19.26) IU/L vs. −10.33 (7.99) IU/L (p = 0.401). There were also no differences in the mean (SD) change in fasting glucose: −0.88 (4.87) mg/dL in the metformin group vs. −3.89 (16.49) mg/dL in the placebo group (p = 0.475).

## Discussion

In this randomised, double-blind, placebo-controlled pilot trial, 96 weeks of metformin was safe and well tolerated in non-diabetic, virologically suppressed adults with HIV aged ≥50 years. Although none of the biomarkers of biological ageing showed statistically significant between-group differences, the direction of change across all biomarkers favoured metformin. Taken together, these exploratory findings and the reassuring safety profile warrant a larger, adequately powered trial to determine whether metformin confers a gerotherapeutic benefit in this population.

We assessed 11 epigenetic biomarkers: four principal component (PC)-derived epigenetic clocks (PC-Horvath, PC-Hannum, PC-PhenoAge and PC-GrimAge); their original counterparts; the DunedinPACE; and the DNAmTL and PC-DNAmTL estimators. Effect sizes were small, with wide confidence intervals, and no between-group differences reached statistical significance over 96 weeks of follow-up. Among these measures PC-GrimAge and PC-Phenoage showed the largest effect favouring metformin. This is interesting given their strong associations with morbidity and mortality in PLWH, as we have previously reported,^26^ and may be suitable endpoints for future geroscience trials.

Recent data in older, non-diabetic PLWH reported metformin-associated reductions in epigenetic age within monocytes (≈1.8 and 3.5 years at 24 weeks for PC-GrimAge and PC-PhenoAge, respectively).^15^ Our whole-blood analyses did not show effects of similar magnitude, possibly reflecting dilution of cell-specific signals in mixed leukocyte populations. Additionally, a small cross-sectional study in people without HIV with type 2 diabetes found lower peripheral-blood EAA among long-term metformin users vs. non-users by Horvath and Hannum clocks, although its non-randomised design limits inference.^12^

We did not observe statistically significant between-group differences in any frailty scale, including Fried phenotype, FRAIL scale, SPPB, or FRAIL-VIG index. This may reflect the relatively low prevalence of frailty at baseline, the limited sample size, and the relatively short follow-up period for detecting meaningful changes in physical function or frailty trajectories. In elderly non-HIV adults, the MET-PREVENT randomised trial likewise found no improvement in four meters walk speed with metformin vs. placebo at four months (adjusted mean difference 0.001 m/s; 95% CI −0.06 to 0.06; p = 0.96), with secondary functional outcomes also null.^27^

Demonstrating the safety of metformin in non-diabetic populations is a critical prerequisite for its potential repurposing as a gerotherapeutic agent. To our knowledge, this is the first randomised, double-blind, placebo-controlled trial to evaluate the long-term safety of metformin in non-diabetic, virologically suppressed PLWH. Despite the preence of age-related comorbidities and polypharmacy, metformin was well tolerated over the 96-week intervention period, with a safety profile comparable to placebo. These findings are consistent with earlier studies in younger PLWH, including the LILAC trial, where metformin was administered for 12 weeks without major safety concerns.^14^ A meta-analysis of randomised trials in PLWH also concluded that metformin is generally well tolerated, although gastrointestinal symptoms such as diarrhoea and nausea were more common than with comparators.^28^ Similarly, the MAVMET trial, which enrolled 29 non-diabetic adults with HIV and confirmed or suspected metabolic dysfunction–associated fatty liver disease, reported an acceptable safety profile for metformin.^29^

In contrast, the MET-PREVENT trial, which investigated metformin in frail, non-diabetic older adults without HIV (mean age 80.4 years) as a geroprotective intervention, reported poor tolerability, including a 38% discontinuation rate and increased hospital admissions in the metformin group.^27^ Compared to that population, our trial included younger participants (median age 56 years) and demonstrated that metformin was well tolerated over nearly two years of treatment, with no drug-related serious adverse events or treatment discontinuations. Diarrhoea was observed in four participants, but in all except one case it was self-limited. Importantly, no episodes of lactic acidosis occurred—unlike in MET-PREVENT, where this was the main serious adverse event reported with metformin.^27^

Adherence to the study medication was high, with most participants completing the 96-week intervention without interruptions. This supports the good tolerability of metformin in this population. The greater increase in GDF15 levels in the metformin group, a known biomarker of metformin exposure, further confirms adherence.

This study has several limitations. The modest sample precludes detecting rare or long-term adverse events and limits power to identify modest between-group differences in epigenetic ageing biomarkers, so efficacy findings should be viewed as exploratory. Generalisability is constrained by the single-country setting, predominance of men, and the predominantly White, self-reported ethnic composition of the sample; findings may not be generalisable to other ethnic groups or settings. We did not collect validated quality-of-life measures. Blinding was operational (identical tablets) but not formally assessed; low-grade gastrointestinal symptoms may have led to partial functional unblinding. Furthermore, despite randomisation, baseline heterogeneity was present between groups, including differences in the prevalence of dyslipidaemia. Although analyses were adjusted for statin use, all efficacy analyses should therefore be considered hypothesis-generating. Epigenetic measurements were derived from whole blood, which may obscure cell-type-specific signals; future work will therefore employ cell-type-specific approaches, particularly in monocytes. The clinical significance of short-term intervention-induced changes in methylation-based biomarkers also remains uncertain. Epigenetic ageing biomarkers have demonstrated independent prognostic value in people with HIV, including in our own cohort,^26^ where higher epigenetic age acceleration was associated with adverse clinical outcomes; however, whether pharmacologically induced changes translate into clinical benefit remains unknown. Establishing whether epigenetic clocks function as true surrogate endpoints—rather than merely prognostic markers—will require trials with hard clinical outcome measures.

In summary, this randomised pilot trial provides reassuring long-term safety data for metformin in non-diabetic, virologically suppressed adults with HIV, along with preliminary signals that could indicate a potential slowing of biological ageing. While exploratory, the consistency of these trends across multiple validated biomarkers supports the rationale for a larger, fully powered clinical trial to precisely assess the gerotherapeutic potential of metformin in this population.

## Contributors

CMC, AEC, BR, RM, JRC, RMC, NGPV, and JRA designed the study. CMC, RM, AGG, JIB, LMC, RMB and JRA recruited participants. CMC, RM, and JRA were responsible for data and sample acquisition. Laboratory sample analyses were performed by AEC, BR, JRC, LGG, and JCG, and JRA, FJ, AEC, and CMC carried out the data analysis. All authors contributed to drafting the manuscript and to its critical review and revision. All authors read and approved the final version of the manuscript. CMC and JRA had full access to all the data in the study and take responsibility for the integrity of the data and the accuracy of the data analysis. The corresponding author (JRA) had the final responsibility for the decision to submit for publication.

## Data sharing statement

Deidentified individual participant data used in the study can be made available for investigators upon request to the corresponding author. Additional trial documents (information sheet, consent form, study protocol, statistical analysis plan) will be also available upon request to the corresponding author.

## Declaration of interests

JRA reports that his institution has received grants from the Instituto de Salud Carlos III, including support for the present manuscript. AEC has received payment or honoraria for presentations from Gilead Sciences and Janssen Cilag. RM has received payment or honoraria for presentations and for attending meetings from Gilead Sciences and ViiV Healthcare, and has participated on advisory boards. AGG has received support for attending meetings from Gilead Sciences and Johnson & Johnson. PMM has received support for attending meetings from Gilead Sciences. RMB has received a grant from Gilead Sciences, has received payment or honoraria for presentations from Gilead Sciences and ViiV Healthcare, has participated on an advisory board for ViiV Healthcare, and has received support for attending meetings from Gilead Sciences and ViiV Healthcare. JIB has received consulting fees and payment or honoraria for presentations from Gilead Sciences, Merck Sharp & Dohme, and ViiV Healthcare. LMC has received consulting fees, support for attending meetings and have participated on advisory board from Gilead Sciences, Janssen and ViiV Healthcare. BR has received support for attending meetings from GlaxoSmithKline and Grupo de Estudio de SIDA. LGG is supported by a PFIS fellowship from Instituto de Salud Carlos III (FI23/00061). All other authors declare no competing interests.

## Appendix A Supplementary data

Supplementary Fig. S1 and Tables S1–S4

Sutdy_Protocol

Translated abstract Spanish
