# Supplementary material for: Metformin and epigenetic age in non-diabetic older people with HIV in Madrid (METFORAGING): a double-blind, randomised, placebo-controlled, pilot trial
Source: eClinicalMedicine. 2026 Apr 18;95:103874. doi: 10.1016/j.eclinm.2026.103874 (PMC13098334; doi:10.1016/j.eclinm.2026.103874)
Supplement: Supplementary Fig. S1 and Tables S1–S4 [file mmc1.docx]

**Supplementary Material**

**Table of Contents**

1. Supplementary Table S1 – Correlation between changes from baseline to week 96 in serum GDF-15 levels and changes in epigenetic ageing biomarkers

2. Supplementary Table S2 – Intragroup differences in epigenetic ageing biomarkers from baseline to week 96

3. Supplementary Table S3 – Frailty status at baseline and week 96 by intervention group

4. Supplementary Table S4 – Adverse events grouped by system organ class and treatment arm

5. Supplementary Figure S1 – Change in serum GDF15 levels after study followup

**Supplementary table S1: Correlation between changes from baseline to week 96 in serum GDF-15 levels and changes in epigenetic ageing biomarkers.**

| Epigenetic ageing biomarker | Rho | p-value |
| --- | --- | --- |
| Horvath | -0·16 | 0·350 |
| PC-Horvath | -0·29 | 0·089 |
| Hannum | -0·08 | 0·664 |
| PC-Hannum | -0·33 | 0·054 |
| PhenoAge | -0·06 | 0·739 |
| PC-PhenoAge | -0·14 | 0·419 |
| GrimAge V2 | -0·10 | 0·570 |
| PC-GrimAge | -0·26 | 0·137 |
| DunedinPACE | -0·22 | 0·205 |
| DNAmTL | -0·02 | 0·919 |
| PC-DNAmTL | 0·28 | 0·100 |

*Spearman correlations were calculated between changes (W96 − baseline) in serum GDF-15 and each epigenetic ageing biomarker. Analyses were performed in the per-protocol population (n = 35).*

**Supplementary table S2:** I**ntragroup differences in epigenetic ageing biomarkers from baseline to week 96.**

|  | **Treatment group** | | | | | |
| --- | --- | --- | --- | --- | --- | --- |
| **Epigenetic biomarker** | **Metformin (n=17)** | | | **Placebo (n=18)** | | |
|  | **Baseline** | **W96** | ***p**** | **Baseline** | **W96** | ***p**** |
| **First-generation clocks:**  Horvath EAA, median (IQR)  Hannum EAA, median (IQR) | -0·37 (-1·91―3·19)  1·39 (-1·06―2·13) | 0·14 (-2·73―2·83)  1·10 (-0·86―2·11) | 0·22  0·58 | -0·27 (-2·24―2·40)  -1·25 (-4·14―2·04) | -0·17 (-2·06―2·51)  -1·10 (-2·24―1·21) | 0·64  0·44 |
| **Second-generation clocks:**  PhenoAge EAA, median (IQR)  GrimAge EAA, median (IQR) | 1·88 (-2·14―3·77)  -0·95 (-3·83―3·68) | -1·19 (-6·23―3·49)  -0·75 (-2·92―1·65) | 0·33  0·96 | -0·69 (-2·82―2·49)  0·22 (-2·92―2·31) | -1·13 (-3·13―4·15)  -0·46 (-2·95―3·70) | 0·90  0·83 |
| **Third-generation clock:**  DunedinPACE, median (IQR) | 1·07 (1·02―1·17) | 1·07 (1·03―1·13) | 0·89 | 1·08 (1·05―1·13) | 1·10 (1·05―1·14) | 0·83 |
| **PC-components clocks:**  PC-Horvath EAA, median (IQR)  PC-Hannum EAA, median (IQR)  PC-PhenoAge EAA, median (IQR)  PC-GrimAge EAA, median (IQR) | 0·19 (-2·50―3·96)  0·92 (-2·00―3·48)  0·62 (-3·67―2·23)  -1·13 (-2·46―1·14) | -0·04 (-3·24―4·22)  0·08 (-2·06―2·88)  0·15 (-4·17―1·77)  -1·35 (-2·57― -0·45) | 0·75  0·40  0·22  0·82 | -0·43 (-2·20―1·98)  -0·43 (-2·98―1·47)  -0·44 (-4·05―2·41)  -0·39 (-1·98―2·26) | 0·41 (-2·18―1·97)  0·42 (-3·18―2·51)  -0·39 (-4·30―2·12)  -0·43 (-1·25―3·33) | 0·32  0·37  0·90  0·067 |
| **DNAmTL**  DNAmTL AA, median (IQR)  PC-DNAmTL AA, median (IQR) | 0·03 (-0·03―0·07)  -0·02 (-0·07―0·05) | 0·03 (-0·04―0·06)  -0·02 (-0·08―0·05) | 0·82  0·71 | 0·04 (-0·09―0·09)  0·02 (-0·11―0·08) | 0·02 (-0·17―0·05)  -0·01 (-0·10―0·09) | **0·038**  0·61 |

**p*-values correspond to the Wilcoxon signed-rank test.

EAA: epigenetic age acceleration; AA: age adjusted; IQR: interquartile range

# **Supplementary table S3. Frailty status at baseline and week 96 by intervention group**

|  | Timepoint | Metformin (n=17) | Placebo (n=18) | p-value |
| --- | --- | --- | --- | --- |
| Fried phenotype |  |  |  | 0·694 |
| Robust | Baseline | 16 (94·1%) | 16 (88·9%) |  |
|  | Week 96 | 10 (58·8%) | 14 (77·8%) |  |
| Pre-frail | Baseline | 1 (5·9%) | 2 (11·1%) |  |
|  | Week 96 | 6 (35·3%) | 4 (22·2%) |  |
| Frail | Baseline | 0 | 0 |  |
|  | Week 96 | 1 (5·9%) | 0 |  |
| FRAIL scale |  |  |  | 1 |
| Robust | Baseline | 14 (82·4%) | 14 (77·8%) |  |
|  | Week 96 | 13 (76·5%) | 13 (72·2%) |  |
| Pre-frail | Baseline | 3 (17·6%) | 4 (22·2%) |  |
|  | Week 96 | 4 (23·5%) | 5 (27·8%) |  |
| Frail | Baseline | 0 | 0 |  |
|  | Week 96 | 0 | 0 |  |
| Frail-VIG index |  |  |  | 1 |
| Fit | Baseline | 17 (100%) | 18 (100%) |  |
|  | Week 96 | 17 (100%) | 18 (100%) |  |
| Mildly frail | Baseline | 0 | 0 |  |
|  | Week 96 | 0 | 0 |  |
| Moderately frail | Baseline | 0 | 0 |  |
|  | Week 96 | 0 | 0 |  |
| Severely frail | Baseline | 0 | 0 |  |
|  | Week 96 | 0 | 0 |  |
| SPPB (mean) | Baseline | 12 (11-12) | 12 (11-12) |  |
|  | Week 96 | 12 (10-12) | 12 (11-12) |  |

Frailty was assessed using the Fried phenotype, FRAIL scale, Frail-VIG index, and Short Physical Performance Battery (SPPB). Data are presented as number and percentage of participants in each category at baseline and week 96, by treatment group, compared between groups using χ² tests. SPPB scores were analysed as continuous variables and are reported as medians with ranges.

**Supplementary table S4: Adverse events grouped by system organ class and treatment arm.**

| **System organ class** | **Disease** | **Metformin**  **(n=48 events)** | **Placebo**  **(n=48 events)** | **Total**  **(n=96 events)** |
| --- | --- | --- | --- | --- |
| Cardiovascular disorders | Hypertension | 1 | 2 | 3 |
| Ear disorders | Peripheral vertigo | 0 | 1 | 1 |
| Endocrine and metabolic disorders | Hemochromatosis | 1 | 0 | 1 |
|  | Hepatic steatosis | 0 | 1 | 1 |
|  | Vitamin D deficiency | 3 | 4 | 7 |
| Gastrointestinal disorders | Diarrhoea | 4 | 0 | 4 |
|  | Flatulence | 1 | 2 | 3 |
|  | Gastroesophageal reflux | 3 | 1 | 4 |
|  | Anal fissure | 0 | 1 | 1 |
|  | Haemorrhoids | 0 | 1 | 1 |
|  | Inguinal hernia | 2 | 0 | 2 |
| Hepatobiliary disorders | Biliary colic | 0 | 1 | 1 |
| Infections | Acute gastroenteritis | 1 | 2 | 3 |
|  | COVID-19 | 4 | 2 | 6 |
|  | Herpes simplex | 0 | 1 | 1 |
|  | Gastrointestinal infections | 1 | 0 | 1 |
|  | Scabies | 1 | 0 | 1 |
|  | STI | 1 | 6 | 7 |
|  | Upper respiratory tract infection | 7 | 3 | 10 |
|  | Urinary tract infection | 3 | 0 | 3 |
| Injury and procedural complications | Allergic reaction to insect bite | 0 | 1 | 1 |
|  | Bone fracture | 0 | 2 | 2 |
| Musculoskeletal and connective disorders | Back pain | 3 | 3 | 6 |
|  | Extremity pain | 2 | 5 | 7 |
| Neoplasms benign, malignant and unspecified | High-grade squamous intraepithelial lesion | 0 | 1 | 1 |
|  | Prostate adenocarcinoma | 0 | 1 | 1 |
| Surgical medical procedures | ERCP | 0 | 1 | 1 |
|  | Knee replacement surgery | 1 | 0 | 1 |
|  | Root canal treatment | 0 | 3 | 3 |
| Nervous system disorders | Headache | 1 | 0 | 1 |
| Renal and urinary disorders | Benign prostatic hyperplasia | 0 | 1 | 1 |
|  | Urinary incontinence | 1 | 0 | 1 |
|  | Acute kidney failure | 2 | 0 | 2 |
|  | Chronic prostatitis | 1 | 0 | 1 |
| Skin and subcutaneous tissue disorders | Psoriasis | 1 | 0 | 1 |
|  | Tinea corporis | 0 | 1 | 1 |
|  | Skin lesion | 2 | 0 | 2 |
| Vascular disorders | Superficial vein thrombosis | 1 | 0 | 1 |
|  | Venous insufficiency | 0 | 1 | 1 |

Data show the number of events per category. One participant may have experienced more than one adverse event. STI= sexually transmitted infection. ERCP: endoscopic retrograde cholangiopancreatography


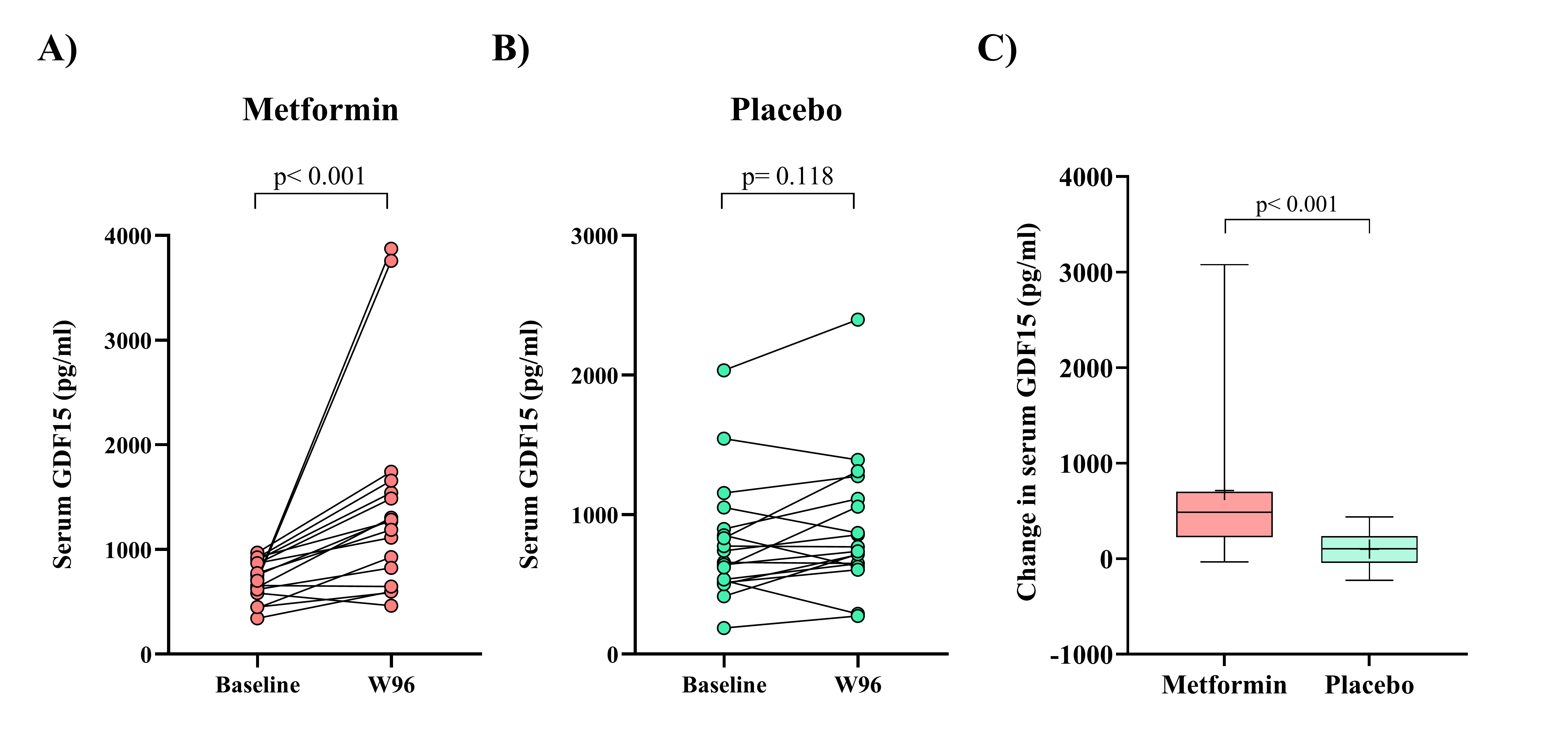


**Supplementary Figure S1. Change in serum GDF-15 levels over 96 weeks.**

(A–B) Serum GDF-15 concentrations (pg/mL) at baseline and week 96 in the metformin and placebo groups; lines connect paired measurements for each participant. (C) Distribution of the change from baseline to week 96 by group. GDF-15 was measured by ELISA. Boxes represent the median and interquartile range (IQR); whiskers indicate the 95% confidence intervals; the “+” symbol denotes the mean.
