# Supplementary material for: Metformin and epigenetic age in non-diabetic older people with HIV in Madrid (METFORAGING): a double-blind, randomised, placebo-controlled, pilot trial
Source: eClinicalMedicine. 2026 Apr 18;95:103874. doi: 10.1016/j.eclinm.2026.103874 (PMC13098334; doi:10.1016/j.eclinm.2026.103874)
Supplement: Sutdy_Protocol [file mmc2.pdf]

Title Page

Protocol Title:

A double blinded, phase II, placebo controlled, single centre randomized clinical trial to evaluate Metformin compared with placebo for reversal of accelerated biological aging in persons living with HIV 50 years or older and with suppressed virologic replication.

Protocol Number and Date: 2.0 / July 3, 202

Compound: Metformin

Brief Title: Metformin vs placebo for reversal of accelerated biological aging in persons living with HIV 50 years or older and with suppressed virologic replication

Study Phase: Phase II

Acronym: METFORAGING

Sponsor Name: FibHULP

Regulatory Agency Identifier Number(s)

EudraCT: 2021-003299-15

© 2015, 2019 TransCelerate BioPharma

Sponsor Signatory:

**[Name]**	**Date**
**[Title]**	

Medical Monitor Name and Contact Information:

Protocol Amendment Summary of Changes Table

DOCUMENT HISTORY	
**Document**	**Date**
Original	31 Mayo 2021
Non-substantial modification number 1	06 December 2021
Non-substantial modification number 2	22 February 2022
Non-substantial modification number 3	06 April 2022
Substantial modification number 1	03 July 2023

Substantial Amendment

Overall Rationale for the Substantial Amendment:

Section # and Name	Description of Change	Brief Rationale

Table of Contents

1. Protocol Summary 7
1.1. Synopsis 7
1.2. Schema 10
1.3. Schedule of Activities (SoA) 11
2. Introduction 14
2.1. Study Rationale – 14
2.2. Background 15
3. Objectives and Endpoints 17
4. Study Design 19
4.1. Overall Design 19
4.2. Scientific Rationale for Study Design 19
4.2.1. Participant Input into Design 19
4.2.2. Rationale for endpoints 19
4.2.3. Rationale for the Use of Comparator/Placebo 19
4.3. Justification for Dose 19
4.4. End of Study Definition 20
5. Study Population 21
5.1. Inclusion Criteria 21
5.2. Exclusion Criteria 21
5.3. Lifestyle Considerations 22
5.3.1. Meals and Dietary Restrictions 23
5.3.2. Caffeine, Alcohol, and Tobacco 23
5.4. Screen Failures 23
5.5. Participant Replacement Strategy 23
6. Study Intervention(s) and Concomitant Therapy 24
6.1. Study Intervention(s) Administered 24
6.2. Preparation/Handling/Storage/Accountability 24
6.3. Measures to Minimize Bias: Randomization and Blinding 25
6.4. Study Intervention Compliance 26
6.5. Dose Modification 27
6.6. Treatment of Overdose 27
6.7. Concomitant Therapy 28
7. Discontinuation of Study Intervention and Participant Discontinuation/Withdrawal - 29
7.1. Discontinuation of Study Intervention 29
7.1.1. Permanent Discontinuation 29
7.1.2. Temporary Discontinuation 29
7.1.3. Rechallenge 30
7.2. Participant Discontinuation/Withdrawal from the Study 30
7.3. Lost to Follow up 31
8. Study Assessments and Procedures 33
8.1. Primary and secondary Assessments 33
8.1.1. Visit Assesments 33
8.2. Safety Assessments 35
8.2.1. Physical Examinations 35

© 2015, 2019 TransCelerate BioPharma

8.2.2. Vital Signs 35
8.2.3. Clinical Safety Laboratory Assessments 35
8.3. Other study laboratory assessments 36
8.4. Frailty Phenotype assessments 399
8.5. Adverse Events (AEs), Serious Adverse Events (SAEs), and Other Safety Reporting 40
8.5.1. Time Period and Frequency for Collecting AE and SAE Information 40
8.5.2. Method of Detecting AEs and SAEs 41
8.5.3. Follow-up of AEs and SAEs 41
8.5.4. Regulatory Reporting Requirements for SAEs 41
9. Statistical Considerations 42
9.1. Statistical Hypotheses 42
9.2. Sample Size Determination 42
9.3. Analysis Sets 42
9.4. Statistical Analyses 43
9.4.1. General Considerations 43
9.4.2. Primary Endpoint(s) 43
9.4.3. Secondary Endpoint(s) 43
9.4.4. Safety Analysis 43
10. Supporting Documentation and Operational Considerations 45
10.1. Appendix 1: Regulatory, Ethical, and Study Oversight Considerations 45
10.1.1. Regulatory and Ethical Considerations 45
10.1.2. Financial Disclosure 45
10.1.3. Informed Consent Process 46
10.1.4. Data Protection 46
10.1.5. Committees Structure 47
10.1.6. Dissemination of Clinical Study Data 47
10.1.7. Data Quality Assurance 47
10.1.8. Source Documents 48
10.1.9. Study and Site Start and Closure 49
10.1.10. Publication Policy 49
10.2. Appendix 2: Clinical Laboratory Tests 51
10.3. Appendix 3: Frailty Phenotype Assessment 55
10.3.1. Fried frailty phenotype criteria 55
10.3.2. Short Physical Performance Battery 62
10.3.3. Gait speed 63
10.3.4. Hand-grip strength 63
10.4. Appendix 4: AEs and SAEs: Definitions and Procedures for Recording, Evaluating, Follow-up, and Reporting 66
10.4.1. Definition of AE 66
10.4.2. Definition of SAE 67
10.4.3. Recording and Follow-Up of AE and/or SAE 68
10.4.4. Reporting of SAEs 70
10.5. Appendix 5: Contraceptive and Barrier Guidance 71

© 2015, 2019 TransCelerate BioPharma

10.5.1. Definitions....71
10.6. Appendix 6: Genetics....73
10.7. Appendix 7: Prohibited concomitant medications....74
10.8. Appendix 8: Lifestyle recommendations....83
10.9. Appendix 9: Information about reasons for temporary or definitive discontinuation of the study drug....86
10.10. Appendix 10: Country-specific Requirements....88
10.11. Appendix 11: Abbreviations....89
10.12. Appendix 12: Protocol Amendment History....91
11. References....92

1. Protocol Summary

1.1. Synopsis

Protocol Title:

A double blinded, phase II, placebo controlled, single center randomized clinical trial to evaluate metformin compared with placebo for reversal of accelerated biological aging in persons living with HIV 50 years or older and with suppressed virologic replication.

Brief Title: Metformin vs placebo for reversal of accelerated biological aging in persons living with HIV 50 years or older and with suppressed virologic replication

Rationale:

Metformin favorably influences metabolic and cellular processes closely associated with the development of age-related conditions, such as inflammation (1), autophagy (2), and cellular senescence (3).

Retrospective data from patients prescribed metformin have concluded that its use is associated with reductions in cardiovascular disease (CVD) incidence and mortality (4), cancer rates (5), depression and frailty-related diseases (6). These data suggest it might be interesting to study the potential anti-aging effects of metformin in humans. There is already one large clinical trial, The Targeting Ageing with Metformin (TAME), an initiative to study the effects on ageing of metformin on 3,000 non-diabetic people, aged 65 -79 years, at multiple centers in the United States (7,8).

Metformin could modulate different mechanisms involved in the ageing process such as genomic instability, cell senescence, stem cell exhaustion, altered intercellular communication or mitochondrial dysfunction.(9) It has been reported an effect of metformin reducing ceramide harmful effects and inhibiting genes coding for multiple inflammatory cytokines.(3) Moreover, metformin could promote health effects through epigenetic changes linked with gut microbiota modulation. (10). Besides, metformin modify cell metabolism suppressing HIV-1 replication in human CD4 T-cells and humanized mice. Notably, clinical data showed that individuals with both HIV-1 and type 2 diabetes mellitus comorbidity exhibited an average 1.33-fold lower HIV-1 viral load than patients with HIV without diabetes among the early cART-treated cohort (6months).

The effects of metformin are to be examined on multiple health age-related markers, including CVD, cancer, dementia and mortality, under the premise that a drug that extends the health span would prevent the onset of many distinct age-related conditions (11). A small, short-term intervention in healthy adults showed that metformin triggers both metabolic and non-metabolic pathways linked to ageing in nondiabetic individuals of average age 70 years (12).

© 2015, 2019 TransCelerate BioPharma

Objectives and Endpoints

Objectives	Endpoints
Primary	
• To evaluate the anti-ageing effect of metformin compared to placebo as assessed by difference in epigenetic age acceleration (EAA) by Phenoage at week 96	• EAA difference by Phenoage epigenetic clock
Secondary	
• To evaluate the anti-ageing effect of metformin compared to placebo as assessed by difference in EAA by four epigenetic clocks at week 48, 96 and 144	• EAA difference by Horvath´s clock, Hannum´s clock, GrimAge and PhenoAge
• To evaluate the effect of metformin compared to placebo as assessed by the immune profile recovery at week 48, 96 and 144	• Immune profile: % and absolute number of $CD4^+$ T cells, $CD8^+$ T cells, $CD4^+/CD8^+$ ratio, haematopoietic progenitors, $CD4^+$ and $CD8^+$ T-cell subsets (recent thymic emigrants, naïve, central and effector memory, TEMRA, activated, exhausted and senescent), T-reg, B-cell subsets (naïve, class-switched memory, non-class switched memory), NK subsets (CD56dim CD16hi, CD56hi CD16-/low) and monocytes (classic, non-classic and intermediate)
• To evaluate the effect of metformin compared to placebo as assessed by the inflammatory biomarkers' changes at week 48, 96 and 144	• Changes in the inflammatory markers: IL-6, CRP, D-Dimer
• To evaluate the effect of metformin compared to placebo as assessed by the leucocyte telomere length changes at week 48, 96 and 144	• Changes in Telomere length in PBMC
• To evaluate the effect of metformin compared to placebo as assessed by the different aging biomarkers changes at week 48, 96 and 144	Changes in the following aging biomarkers:<br>• TAME biomarkers: IL-6, TNFR II, hsCRP, GDF15, IGF-1, fasting insulin, cystatin C; NT-proBNP, haemoglobin A1c.<br>• Oxidative stress and DNA damage: reactive oxygen species (ROS), catalase

	expression, superoxide dismutase 1 - 2 levels, γH2AX histone levels.<br>• Other inflammatory and pro-coagulant biomarkers: IL-1 beta, TNF-alfa, D-dimer
• To evaluate the effect of metformin compared to placebo as assessed by the frailty phenotype improvement at week 48, 96 and 144	• Changes in the following Frailty battery: Fried frailty index, grip strength, walking speed and short physical performance battery
• To evaluate the security of metformin compared to placebo as assessed by lab parameters at week 24, 48, 72, 96, 120 and 144	• Changes in creatinine
• To evaluate the effect of metformin compared to placebo as assessed by the viral reservoir parameters´ changes at week 48	• Changes in integrated and total (Gag) HIV-DNA<br>• Changes in CA US HIV-RNA<br>• Changes in intact proviral DNA

Overall Design: A double blinded, phase II, placebo controlled, single centre randomized clinical trial. Follow up: 144 weeks

Brief Summary:

The purpose of this study is to evaluate the efficacy of metformin compared to placebo as assessed by the difference in epigenetic age acceleration in participants with HIV infection.

Study Duration: 144 weeks

Treatment Duration: 96 weeks

Visit Frequency: 0, 4 weeks, 8 weeks, 24 weeks and every 24 weeks after week 24 of follow-up.

Number of Participants:

Approximately 80 participants will be screened to achieve 30 randomly assigned to study intervention for an estimated total of 30 evaluable participants per intervention group.

A maximum of 40 participants will be randomly assigned to study intervention such that approximately 30 evaluable participants complete the study per intervention group.

Note: “Enrolled” means a participant’s, or their legally acceptable representative’s, agreement to participate in a clinical study following completion of the informed consent process. Potential participants who are screened for the purpose of determining eligibility for the study, but do not participate in the study, are not considered enrolled, unless otherwise specified by the protocol.

Intervention Groups and Duration:

Metformin (Metformin 850 mg film-coated tablet):

Route of administration: oral.

Dose: Metformin 850 mg/day.

Dosage: one 850 mg film coated-tablet once daily during the first 4 weeks followed by one 850 mg film coated-tablet twice daily 92 weeks, except in persons receiving dolutegravir who would maintain the 850 mg dose once daily due to the pharmacological interaction

Intervention: starting Metformin 850mg treatment.

1.2. Schema

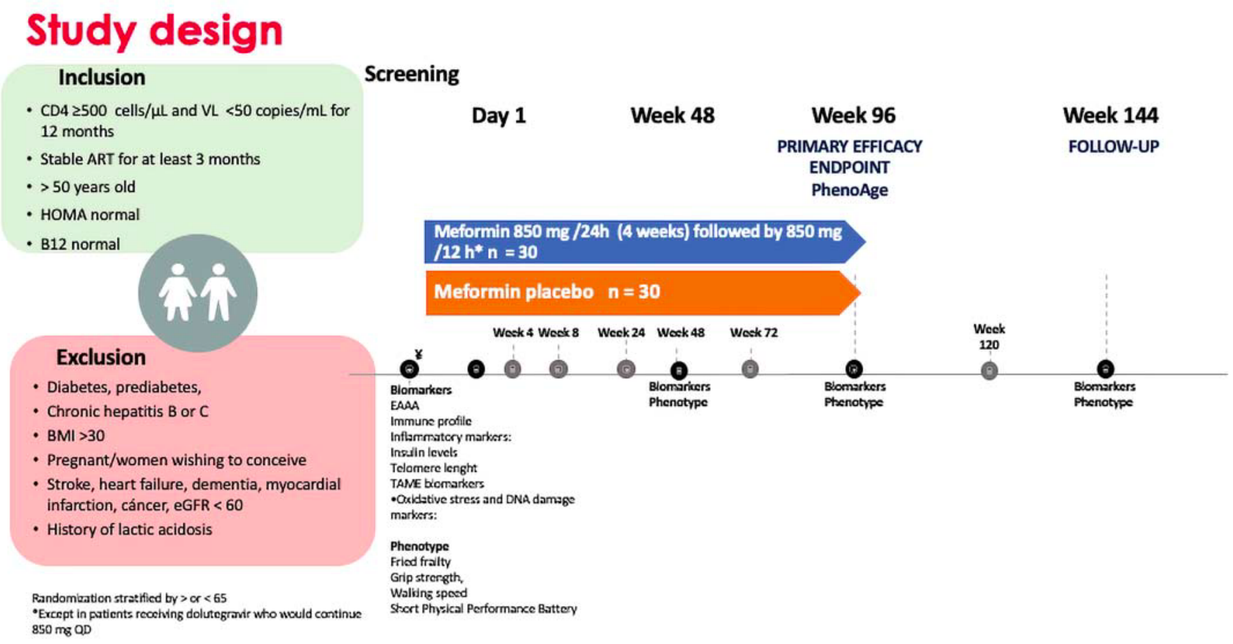

1.3. Schedule of Activities (SoA)

Procedure	Screening (up to 30 days before Day 0)	Intervention Period, Visit number [Week]									E/D[1]
		1 [0]	2 [4]	3[17] [8]	4 [24]	5 [48]	6 [72]	7 [96]	8 [120]	9 [144]	
Informed consent	X										
Inclusion and exclusion criteria	X	X									
Randomization		X									
Demography and Medical history (include substance usage, medical conditions, HIV history, and family history of premature cardiovascular disease)	X										
Past and current medical conditions	X										
AE and SAE review		X	X	X	X	X	X	X	X	X	X
Concomitant medication review and interactions[2]	X	X	X	X	X	X	X	X	X	X	X
Vital signs[3] and complete physical exam including anthropometric measurements[4]	X					X		X		X	X
Vital signs and symptom guided physical exam[5]		X	X	X	X	X	X	X	X	X	X
FSH levels and menopause history[6]	X										
Full safety laboratory panel[7] (include hemogram, fasting glucose, kidney and liver profile, fasting lipids, proteins, albumin, vitamin D and urine)	X	X			X	X	X	X	X	X	X
Limited safety laboratory panel[7] (include hemogram, fasting glucose, kidney and liver profile, and urine)			X	X							
B12 vitamin levels	X										
CMV, Hepatitis B and C screening[8]	X										
HIV-1 Viral load	X				X	X	X	X	X	X	X
CD4 cell count					X		X		X		

**Procedure**	**Screening (up to 30 days before Day 0)**	**Intervention Period, Visit number [Week]**									**E/D[1]**
		**1 [0]**	**2 [4]**	**3[17] [8]**	**4 [24]**	**5 [48]**	**6 [72]**	**7 [96]**	**8 [120]**	**9 [144]**	
CD4 and CD8 cell count	X	X				X		X		X	X
Glycated hemoglobin (A1C), and HOMA-IR	X	X				X		X		X	X
Laboratory test: C-Reactive Protein, D-Dimer, IGF-1, fasting insulin, cystatin C; NT-proBNP		X			X	X	X	X	X		
Laboratory tests for epigenetic study, complete immune profile[9], blood leucocyte telomere length, oxidative stress levels and DNA damage markers, TAME trial biomarkers[10]		X				X		X		X	
Frailty assessments and geriatric scale		X				X		X		X	
Dispense of study medication		X	X	X	X	X	X				
Return of medication and treatment compliance[12]			X	X	X	X	X	X			
Study drug titration to twice a day[13]			X								
Contraceptive and Barrier Guidance[14]	X										
Advice for healthy lifestyle and physical exercise[15]		X				X		X			
Inform about reasons for temporary or definitive discontinuation of the study drug[16]		X				X		X			

Procedure	Screening (up to 30 days before Day 0)	Intervention Period, Visit number [Week]									E/D[1]
		1 [0]	2 [4]	3[17] [8]	4 [24]	5 [48]	6 [72]	7 [96]	8 [120]	9 [144]	

[1] E/D = Early Discontinuation.

[2] See appendix 7

[3] Blood pressure, heart rate.

[4] Weight, height, body mass index, waist circumference, blood pressure, and heart rate.

[5] Symptom guided physical examination will include, at a minimum, assessments of the skin, lungs, cardiovascular system, and abdomen (liver and spleen). Blood pressure and heart rate will also be measured and recorded.

[6] FSH level for female participants without period in the last year only. See Appendix 5.

[7] Hemogram, fasting Glucose, Creatinine, Sodium, Potassium, Estimated GFR, ALT,AST, Alkaline phosphatase, GGT, Total Bilirubin, Fasting lipids, Urinalysis.

[8] Hepatitis B virus surface antigen and Hepatitis C virus antibody test or RNA test (in the case of a previous positive antibody test).

[9] Flow cytometry and inflammatory markers (Il-1β, TNFα, Il-6, C-reactive protein, D-dimer).

[10] IL-6, TNFα receptor, C-Reactive Protein, GDF15, IGF-1, fasting insulin, cystatin C, NT-proBNP, haemoglobin A1c

[11] Fried frailty index, grip strength, walking speed, Short Physical Performance Battery. Geriatric scale: mini nutritional assessment and rockwood scale (section 10.3.5).

[12] Includes active questioning and pill count

[13] Except in patients receiving dolutegravir, who would maintain the 850 mg dose (or placebo) once daily due pharmacological interaction.

[14] See appendix 5

[15] See appendix 8

[16] See appendix 9

[17] This visit will be performed only in patients in whom the dose of metformin has been increased to 850mg dose twice daily in week 4.

© 2015, 2019 TransCelerate BioPharma

2. Introduction

Among the pharmacologic agents investigated for preventing aging, metformin has demonstrated antiaging effects based on preclinical experiments, animal models, and numerous retrospective data on beneficial health outcomes for type 2 diabetics.

2.1. Study Rationale –

Metformin is a biguanide drug derived from French lilac, widely prescribed for type 2 diabetes (13) that interacts with several known longevity pathways. It reduces diabetic hyperglycemia by suppressing hepatic gluconeogenesis inducing glycolysis and increasing insulin sensitivity (14), reduces lipolysis and lowers levels of circulating free fatty acids.

Specifically, for aging, metformin leads to decreased insulin levels, decreased IGF-1 signaling (15), inhibition of mTOR (16),inhibition of mitochondrial complex 1 in the electron transport chain and reduction of endogenous production of reactive oxygen species (ROS) (17), activation of AMP-activated kinase (AMPK) (18), and reduction in DNA damage (19). Metformin favorably influences metabolic and cellular processes closely associated with the development of age-related conditions, such as inflammation (1), autophagy (2), and cellular senescence (3).

Retrospectively, observational analyses of data from patients prescribed metformin have concluded that its use is associated with reductions in cardiovascular disease (CVD) incidence and mortality (4), cancer rates (5), depression and frailty-related diseases (6). These data have led to perform clinical trials to assess the anti-aging effects of metformin in humans. One is The Targeting Ageing with Metformin (TAME), an initiative to study the effects of metformin on 3,000 non-diabetic people, aged 65 -79 years, at multiple centers in the United States (7,8). The effects of metformin are to be examined on multiple markers of age-related health, including CVD, cancer, dementia and mortality, under the premise that a drug that extends the health span would prevent the onset of many distinct age-related conditions (11). A small, short-term intervention in healthy adults has also been performed, showing that metformin triggers both metabolic and non-metabolic pathways linked to ageing in nondiabetic individuals of average age 70 years (12). Other clinical trials evaluating metformin are now in progress (33).

The TRIIM (Thymus Regeneration, Immunorestoration, and Insulin Mitigation) clinical trial showed that the administration of metformin combined with recombinant human growth hormone (rhGH) and dehydroepiandrosterone (DHEA) in ten healthy men was associated with an improvement of epigenetic aging and thymic and bone marrow regenerative responses (20). To our knowledge, the TRIIM study was the first clinical trial considering epigenetic clocks as primary endpoints to measure aging. This pilot study has recently been extended to 85 healthy volunteers to confirm these findings.

In the context of HIV, the Lilac study is a pilot trial in progress on 22 antiretroviral therapy-treated participants living with HIV focusing on immune activation and HIV reservoirs. They propose that metformin may enhance T-cell effector function to decrease the HIV reservoir and

suppress inflammatory mediators and circulating cytokines given metformin immunomodulatory properties (21). Another study showed that metformin reduces circulating levels of exhausted CD4 T lymphocytes in HIV-infected adults on suppressive antiretroviral therapy (22). Taking into account the aforementioned anti-aging effects of metformin, this drug may exhibit senolytic activity in the context of chronic HIV infection.

2.2. Background

Aging refers to the changes that occur during an organisms' life-span, though the rate at which these take place varies widely (23). Consequently, such definition comprises the progressive deterioration of bodily functions over time. Normal human aging has been associated with a loss of complexity in a wide range of physiological processes and anatomic structures (24), leading to impaired function and increased morbidity and mortality. Metabolic, cardiovascular, neoplastic and neurodegenerative disorders as well as geriatric symptoms like frailty and immobility are a consequence of this physiological deterioration (9). Although it is evident that many elderly people reach advanced ages in healthy conditions, the prevalence of age-dependent disease and the average age of patients who enter the clinic is increasing. Millions of people die every year from these medical conditions associated to advancing age and the medical cost trying to mitigate them is staggering (25).Yet the risk of suffering age-related conditions in later life can be modified for most people by intervening in biological systems, through caloric restriction diet, exercise, and therapeutic interventions (26).

Certain disorders and diseases have some of the same effects as aging. HIV infection is one of these examples. It remains uncertain if HIV infection causes accelerated aging (age associated diseases occurring earlier than in the HIV negative population) or accentuated aging (age associated diseases occurring at the same age than in the HIV negative population but with increased frequency). However, what it is beyond doubt is that untreated HIV infection causes an accelerated aging of the human immune system, an alteration also known as immunosenescence. Indeed, HIV infection is considered a model of accelerated immunosenescence (27).

Antiretroviral therapy (ART) has transformed the natural course of HIV infection into a chronic and manageable disease in which life expectancy among people living with HIV (PLWH) approaches that of the general population (28) This prolonged survival in PLWH has been linked with both an increased burden and a premature risk of suffering age-related comorbidities such as cardiovascular disease, cancer, cognitive impairment, osteoporosis and frailty (29,30). Mortality rates in HIV-infected populations range from 1.7- to 7.0-times those of HIV-uninfected populations (31). Thus, persons with HIV on ART have far less life-threatening acute illnesses but must confront issues related to the aging process.

Scientific efforts are underway to understand and intervene in the underlying processes of aging. From the cellular perspective, there are several mechanisms that are considered to affect the primary aging process, which include damage to the genetic material, epigenetic changes, telomere attrition, cellular senescence, proteostasis loss, mitochondrial dysfunction, deregulated nutrient sensing, altered intercellular communication and stem cell exhaustion (9). These hallmarks and their interconnectivity have led to a search for biomarkers of aging, biological

parameters that capture age-related changes and the susceptibility to disease and loss of function. Therefore, clinical interventions targeting the aging process should result in changes in biomarkers that will eventually delay the incidence, accumulation, clinical evolution and functional consequences of age-related diseases. Several biomarkers of aging have been postulated, as telomere length, epigenetic changes, levels of certain serum proteins, immunological markers, systemic inflammation parameters or metabolic and oxidative stress markers (32). However, the multi-factorial nature of aging makes it difficult to find a single measurement useful to determine biological age, so a combination of different parameters seems be appropriate.

In the last years, epigenetic clocks have emerged as promising biomarkers of biological age. They are mathematical algorithms that predict epigenetic age as a surrogate of biological age based on the DNA methylation levels of different sets of CpG dinucleotides sites in the genome that are known to change with aging. Horvath and Hannum´s epigenetic clocks and specially the PhenoAge and the GrimAge, the last clocks to be developed, are attractive tools because they have a strong correlation with chronological age and consistently predict risk of mortality and age-related clinical outcomes (33–35).However, currently there are almost no data about the evolution of these biomarkers after successful treatment of diseases that shorten lifespan or after interventions aimed at preventing aging.

Several interventions are currently tested to directly tackle the aging mechanisms and repair the cell and physiological damage. In recent years, numerous molecules have emerged as potential geroprotective agents, not only to prevent frailty and disease but also to reverse aging. Some of these molecules are still in the preclinical and initial experimental phases but many others have reached clinical testing. The first compounds to formally enter aging clinical trials were senolytic drugs capable of destroying senescent cells. These drugs are used in small studies focused to attenuate aging in specific conditions such as osteoarthritis, Alzheimer's disease or chronic kidney disease(36).

3. Objectives and Endpoints

Objectives	Endpoints
Primary	
• To evaluate the anti-aging effect of metformin compared to placebo as assessed by difference in epigenetic age acceleration (EAA) by Phenoage at week 96<br><br>Hypothesis (H1): Metformin is superior to placebo as assessed by changes in the EAA by Phenoage	• EAA difference by Phenoage epigenetic clock
Secondary	
• To evaluate the anti-aging effect of metformin compared to placebo as assessed by difference in EAA by four epigenetic clocks at week 48, 96 and 144	• EAA difference by Horvath´s clock, Hannum´s clock, GrimAge and PhenoAge
• To evaluate the effect of metformin compared to placebo as assessed by the increase in immune profile recovery at week 48, 96 and 144	• Immune profile: % and absolute number of $CD4^+$ T cells, $CD8^+$ T cells, $CD4^+$/$CD8^+$ ratio, haematopoietic progenitors, $CD4^+$ and $CD8^+$ T-cell subsets (recent thymic emigrants, naïve, central and effector memory, TEMRA, activated, exhausted and senescent), T-reg, B-cell subsets (naïve, class-switched memory, non-class switched memory), NK subsets (CD56dim CD16hi, CD56hi CD16-/low) and monocytes (classic, non-classic and intermediate)
• To evaluate the effect of metformin compared to placebo as assessed by the reduction of inflammatory biomarkers' changes at week 48, 96 and 144	• Changes in the inflammatory markers: IL-6, CRP, D-Dimer
• To evaluate the effect of metformin compared to placebo as assessed by the leucocyte telomere length changes at week 48, 96 and 144	• Changes in Telomere length in PBMC
• To evaluate the effect of metformin compared to placebo as assessed by the different aging biomarkers changes at week 48, 96 and 144	Changes in the following aging biomarkers:<br><br>• TAME biomarkers: IL-6, TNFR II, hsCRP, GDF15, IGF-1, fasting insulin, cystatin C; NT-proBNP, haemoglobin A1c.<br><br>• Oxidative stress and DNA damage: reactive oxygen species (ROS), catalase

	expression, superoxide dismutase 1 - 2 levels, γH2AX histone levels.<br>• Other inflammatory and pro-coagulant biomarkers: IL-1 beta, TNF-alfa, D-dimer
• To evaluate the effect of metformin compared to placebo as assessed by the frailty phenotype improvement at week 48, 96 and 144	• Changes in the following frailty battery: Fried frailty index, grip strength, walking speed and short physical performance battery
• To evaluate the security of metformin compared to placebo as assessed by lab parameters at week 24, 48, 72, 96, 120 and 144	• Changes in creatinine
• To evaluate the effect of metformin compared to placebo as assessed by the viral reservoir parameters´ changes at week 48	• Changes in integrated and total (Gag) HIV-DNA<br>• Changes in CA US HIV-RNA<br>• Changes in intact proviral DNA

4. Study Design

4.1. Overall Design

A double blinded, phase II, placebo controlled, single center randomized clinical trial.

Follow up: 144 weeks

4.2. Scientific Rationale for Study Design

Randomized, placebo-controlled, double-blind superiority design.

4.2.1. Participant Input into Design

The development of the research question and outcome measures were based on the experience of the clinical team treating this profile of patients. Patients were not involved in the design, recruitment or conduct of this study. The patients or their families will be notified of the study results in writing and verbally, and we will invite them to help us develop our dissemination strategy.

4.2.2. Rationale for endpoints

The primary endpoint selected in this study, differences in epigenetic age acceleration, is intended to demonstrate the efficacy of metformin relative to placebo.
This is a phase II, double blinded, placebo controlled, single centre randomized clinical trial to gain preliminary evidence about the efficacy and safety of metformin for reversal of accelerated epigenetic aging in persons ≥ 50 years living with HIV and with suppressed virological replication. We have chosen ≥ 50 years because this is the consensus cut-off that defines to define medically advanced age in HIV-infected people (AIDS Research and Human Retroviruses 2012; 28:1000–1006). This is the first time that the efficacy of an intervention against accelerated/accentuated aging is going to be measured by epigenetic clocks in people living with HIV

4.2.3. Rationale for the Use of Comparator/Placebo

This study will be placebo-controlled in order to avoid bias in the collection/evaluation of data during study conduct and to assess whether any observed effects are related to the drug in the experimental arm.

4.3. Justification for Dose

The recommended starting dose of Metformin hydrochloride tablets for glucose control are 500 mg orally twice a day or 850 mg once a day, given with meals. Diabetes Guidelines recommend dose increments of 500 mg weekly or 850 mg every 2 weeks on the basis of glycaemic control

and tolerability, up to a maximum dose of 2550 mg per day, given in divided doses. Maximum recommended dose is 3 g a day preferably 3 times a day for better tolerability.

The dose used in other antiaging studies range from 1500 to 2000 mg a day. Based on our experience in people with diabetes, we have chosen to start with 850 g once daily and scale up to 850 mg BID in 4 weeks, as this is the standard and best tolerated dose in terms of gastrointestinal symptoms.

Exceptions will be made in persons receiving dolutegravir who would maintain the 850 mg dose QD due to the pharmacological interaction. Dolutegravir is a known inhibitor of OCT2. Consistent with inhibition of OCT2, co-administration of dolutegravir increased the exposure and decreased the clearance of metformin, an effect that was dose dependent. The magnitude to which dolutegravir increased the exposure to metformin was higher than anticipated (5%) based on the IC50 values for OCT2 (1.9 μM), dolutegravir unbound Cmax (twice daily Cmax = 13 mM; free fraction, ≥ 1.1%), and the fraction of metformin clearance mediated by active tubular secretion (0.67).(37) Metformin dose will also be adjusted to glomerular filtration rate (See Table 1 Section 6.5)

4.4. End of Study Definition

The end of the study is defined as the date of the last visit of the last participant in the study or last scheduled procedure shown in the Schedule of Activities for the last participant in the trial globally.

A participant is considered to have completed the study if he/she has completed all phases of the study including the last visit or the last scheduled procedure shown in the Schedule of Activities.

5. Study Population

Male/female participants ≥50 years of age with HIV-1 infection will be enrolled in this study. Prospective approval of protocol deviations to recruitment and enrollment criteria, also known as protocol waivers or exemptions, are not permitted.

5.1. Inclusion Criteria

Participants are eligible to be included in the study only if all of the following criteria apply:

Age

1. Participant must be 50 years old or older, at the time of signing the informed consent.

Type of Participant and Disease Characteristics

2. Participants with HIV-1 infection and an uninterrupted ART regimen in the 3 months prior to study entry
    a. Only switch for tolerability/convenience/access reasons to generic drugs or switch from ritonavir to cobicistat would be allowed in the 3-month window and as long as the components of the regimen are unchanged.
3. HIV viral load (VL) <50 copies/mL at screening and in the year prior to study entry.
    a. A blip (50-200 copies/ml) would be allowed within 12 months prior to inclusion in the study, if preceded and followed by an undetectable VL determination.
4. CD4 count > 500 cel/µL at screening.
5. Participants with normal vitamin B12 levels at screening
6. Participants with normal HOMA-IR (≤ 2.6)

Weight

7. Body mass index (BMI) less than 30 $Kg/m^{2.}$

Sex and Contraceptive/Barrier Requirements

8. Female participants with suspected or documented menopause
    a. Peri- or post-menopausal, defined as having no menstrual periods for at least 12 months prior to study entry, or skipping at least one menstrual period in the 12 months prior to study entry. Peri- or post-menopausal status will be determined for candidates who have had the uterus removed by an assessment of blood follicle stimulating hormone (FSH). Women with an FSH level higher than 30 mlU/mL will be eligible for the study.

Informed Consent

9. Capable of giving signed informed consent as described in Appendix 1 which includes compliance with the requirements and restrictions listed in the informed consent form (ICF) and in this protocol.

5.2. Exclusion Criteria

Participants are excluded from the study if any of the following criteria apply:

Medical Conditions

1. Participants previously diagnosed with diabetes mellitus, prediabetes and body mass index > 30 kg/m^2.
2. Participants with chronic hepatitis B and/or active hepatitis C or concurrent active or progressive liver disease.
3. Participants with history of any of the following comorbidities: stroke, heart failure, dementia, myocardial infarction and cancer or history of lactic acidosis.
4. Participants with decreased tissue perfusion or hemodynamic instability due to infection or other causes
5. Participants with active alcohol abuse:
   a. For men, heavy drinking is typically defined as consuming 15 drinks or more per week.
   b. For women, heavy drinking is typically defined as consuming 8 drinks or more per week.
6. Participants unable to swallow study medication tablets during the treatment period.

Prior/Concomitant Therapy

7. Participants receiving other medications that according to study drug label are contraindicated with metformin.
8. Participants with hypersensitivity or intolerance to any of the components of the study interventions as determined by the investigator.

Prior/Concurrent Clinical Study Experience

9. Participants that are unwilling to abstain from participating in another interventional clinical trial during the study follow up.

Diagnostic assessments

10. Impaired renal function (estimated glomerular filtration rate <60 mL/min).
11. Liver laboratory abnormalities: alanine aminotransferase (ALT) over 5 times the upper limit of normal (ULN) or ALT over 3xULN and bilirubin over 1.5xULN or any verified Grade 4 laboratory abnormality that to the investigators criteria would affect the safety of the participant if included in the study.

Other Exclusions

12. Pregnant or breastfeeding women, women wishing to conceive or unwilling to commit to contraceptive methods.
13. Any comorbidities or treatment with experimental drugs that according to the investigator could bias study results or entail additional risks for the participant.

5.3. Lifestyle Considerations

All participants will receive advice for healthy lifestyle and physical exercise. A specific lifestyle information sheet (see Appendix 7) will be dispensed at baseline visit, week 48 and week 96.

5.3.1. Meals and Dietary Restrictions

There are no meals and dietary restrictions.

In pharmacokinetics studies, food slightly reduces and delays metformin absorption, but its clinical relevance is unknown.

Taking metformin during or after meals improves tolerability and reduces gastrointestinal symptoms so it is strongly recommended.

Participant will receive dietary recommendations in lifestyle information sheet. (See Appendix 8)

5.3.2. Caffeine, Alcohol, and Tobacco

There are no caffeine, and tobacco restrictions. However, participants with alcohol abuse define in section 5.2 will be excluded. A specific lifestyle information sheet (see Appendix 8) will be dispensed at baseline, visit week 48 y week 96.

5.3.3. Activity

All participants will receive advice for healthy lifestyle and physical exercise. A specific lifestyle information sheet (see Appendix 8) will be dispensed at baseline, visit week 48 y week 96.

5.4. Screen Failures

Screen failures are defined as participants who consent to participate in the clinical study but are not subsequently randomly assigned to study intervention/entered in the study. A minimal set of screen failure information is required to ensure transparent reporting of screen failure participants to meet the Consolidated Standards of Reporting Trials (CONSORT) publishing requirements and to respond to queries from regulatory authorities. Minimal information includes demography, screen failure details, eligibility criteria, and any serious adverse event (SAE).

Individuals who do not meet the criteria for participation in this study (screen failure) may be rescreened. Rescreened participants should be assigned a new participant number for every screening/rescreening event.

5.5. Participant Replacement Strategy

Participants who withdraw from the study before receiving their first dose of study treatment or during the first 8 weeks of study medication due to AEs or intolerance to investigational product will be replaced, whereas participants who withdraw from the study after 8 weeks of the study medication will not be replaced.

6. Study Intervention(s) and Concomitant Therapy

Study intervention is defined as any investigational intervention(s), marketed product(s), placebo, or medical device(s) intended to be administered to a study participant according to the study protocol.

6.1. Study Intervention(s) Administered

**ARM Name**	Intervention	Control
**Intervention Name**	Metformin	Placebo comparator
**Type**	Drug	Drug
**Dose Formulation**	Film-coated tablet	Film-coated tablet
**Unit Dose Strength(s)**	850 mg	N/A
**Dosage Level(s)**	One tablet once daily for 4 weeks, followed by one table twice daily	One tablet once daily for 4 weeks, followed by one table twice daily
**Route of Administration**	Oral	Oral
**Use**	Experimental	Placebo
**IMP and NIMP**	IMP	IMP
**Sourcing**	Provided centrally by the trial site	Provided centrally by the trial site
**Packaging and Labeling**	Study Intervention will be provided in boxes. Each box will be labeled as required per country requirement.	Study Intervention will be provided in boxes. Each box will be labeled as required per country requirement.

6.2. Preparation/Handling/Storage/Accountability

1. The investigator or designee must confirm appropriate temperature conditions have been maintained during transit for all study intervention received and any discrepancies are reported and resolved before use of the study intervention.
2. Only participants enrolled in the study may receive study intervention and only authorized site staff may supply or administer study intervention. All study intervention

© 2015, 2019 TransCelerate BioPharma

must be stored in a secure, environmentally controlled, and monitored (manual or automated) area in accordance with the labeled storage conditions with access limited to the investigator and authorized site staff.

3. The investigator, institution, or the head of the medical institution (where applicable) is responsible for study intervention accountability, reconciliation, and record maintenance (i.e., receipt, reconciliation, and final disposition records).
4. For all study sites, the local country Sponsor personnel or designee will provide appropriate documentation that must be completed for drug accountability and return, or local discard and destruction if appropriate. Where local discard and destruction is appropriate, the investigator is responsible for ensuring that a local discard/destruction procedure is documented.
5. The study site is responsible for recording the lot number, manufacturer, and expiry date for any locally purchased product (if applicable) as per local guidelines unless otherwise instructed by the Sponsor.

6.3. Measures to Minimize Bias: Randomization and Blinding

**Randomization**	All participants will be centrally assigned to randomized study intervention using REDCap program. Before the study is initiated, the log in information and training the REDCap will be provided to investigators.<br><br>Study intervention will be dispensed at the study visits summarized in the SoA.<br><br>Returned study intervention should not be re-dispensed to the participants.<br><br>The randomization list will be generated using software SAS 9 for Windows. The randomization list will be imported into the REDCap program so the researchers can randomize candidate subjects using an easier-to-use interface. Subjects that meet the selection criteria will be randomized in a 1:1 ratio between the two treatment groups (Intervention arm: Metformin vs Control arm: placebo), stratifying by age in order to achieve balanced randomization in the two treatment groups. The assignment of treatment to each subject will be centralized, keeping the sequence hidden.

**Blind Break**	In case of an emergency, the investigator has the sole responsibility for determining if unblinding of a participants' intervention assignment is warranted. Participant safety must always be the first consideration in making such a determination. If the investigator decides that unblinding is warranted, the investigator should make every effort to contact the sponsor prior to unblinding a participant's intervention assignment unless this could delay emergency treatment of the participant. If a participant's intervention assignment is unblinded, the sponsor must be notified within 24 hours after breaking the blind. The date and reason that the blind was broken must be recorded in the source documentation.
**Blinded study with unblinded site pharmacist who is dispensing intervention**	Participants will be randomly assigned in a [1:1] ratio to receive study intervention. Investigators will remain blinded to each participant's assigned study intervention throughout the course of the study. To maintain this blind, an otherwise uninvolved 3rd party will be responsible for the dispensation of all study intervention and will endeavor to ensure that there are no differences in time taken to dispense following randomization.<br><br>This 3rd party will instruct the participant or legally authorized representative to avoid discussing the taste, dosing frequency, or packaging of the study intervention with the investigator.<br><br>In the event of a Quality Assurance audit, the auditor(s) will be allowed access to unblinded study intervention records at the site(s) to verify that randomization/dispensing has been done accurately.

A double-blinding technique with in-house blinding will be used. Metformin and placebo will be packaged identically so the blind is maintained. The participant, the investigator, and Sponsor personnel or delegate(s) who are involved in the study intervention administration or clinical evaluation of the participants are unaware of the intervention assignments.

Sponsor safety staff may unblind the intervention assignment for any participant with an SAE. If the SAE requires that an expedited regulatory report be sent to one or more regulatory agencies, a copy of the report, identifying the participant's intervention assignment, may be sent to investigators in accordance with local regulations and/or sponsor policy.

6.4. Study Intervention Compliance

When participants self-administer study intervention(s) at home, compliance with study intervention will be assessed at each visit. Direct questioning will assess compliance and counting returned tablets during the site visits and documented in the source documents and relevant form. Deviation(s) from the prescribed dosage regimen should be recorded.

A record of the number of tablets of metformin or placebo dispensed to and administered by each participant must be maintained and reconciled with study intervention and compliance records.

Intervention start and stop dates, including dates for intervention delays and/or dose reductions will also be recorded.

6.5. Dose Modification

This protocol allows some alteration from the currently outlined dosing schedule, but the maximum daily dose will not exceed.

If there are changes in the antirretroviral treatment, such as starting or stoping dolutegravir, the dose of metformin should be modified accordingly. If dolutegravir is initiated the dose of metformin should be reduced to 850 mg once a day. If dolutegravir is stopped, metformin dosing should be modified to 850 mg dose twice daily.

In those participants that experienced grade 2 gastrointestinal adverse events after metformin dose increase, dose of metformin will be reduced dose to 850 mg once daily.

In case a dose reduction is necessary due to renal impairment, the study intervention will be administered as follows:

Table 1. Metformin dosages adjustment in patients that develop with renal impairment.

CKD stage 2-3a<br>eGFR ≥60 mL/min	CKD stage 3b<br>eGFR ≥45 mL/min	CKD stage 4<br>eGFR <30 mL/min
No dose adjustment is needed.	Reduce dose to 850mg once daily	Stop

6.6. Treatment of Overdose

For this study, any dose of metformin greater than three times daily within a 24-hour time period will be considered an overdose.

Summary of product characteristics does not recommend specific treatment for an overdose unless lactic acidosis is developed. A large overdose or concomitant risks of metformin, such as dehydration or acute renal failure, can lead to lactic acidosis. Lactic acidosis is a medical emergency and must be treated in a hospital. The most effective method of removing lactates and metformin is by hemodialysis.

In the event of an overdose, the investigator should:

- Contact the Medical Monitor immediately.
- Evaluate the participant to determine, in consultation with the Medical Monitor, whether study intervention should be interrupted or whether the dose should be reduced.
- Closely monitor the participant for any AE/SAE and laboratory abnormalities until study intervention can no longer be detected systemically (at least 7 days).

- Document the quantity of the excess dose as well as the duration of the overdose.

6.7. Concomitant Therapy

Any medication or vaccine (including over the counter or prescription medicines, recreational drugs, vitamins, and/or herbal supplements or other specific categories of interest] that the participant is receiving at the time of enrollment or receives during the study must be recorded along with:

- Reason for use
- Dates of administration including start and end dates
- Dosage information including dose and frequency

The Medical Monitor should be contacted if there are any questions regarding concomitant or prior therapy.

Participants must abstain from taking prescription or nonprescription drugs (including vitamins recreational drugs, and dietary or herbal supplements) within 7 days (or 14 days if the drug is a potential enzyme inducer) or 5 half-lives (whichever is longer) before the start of study intervention until completion of the follow-up visit, unless, in the opinion of the investigator and sponsor, the medication will not interfere with the study.

Metformin is a substrate for the OCT1 and OCT2 transporters. Co-administration of metformin with other drug that are inducers or inhibitors of these transporters should be monitored closely.

Following prior and concomitant therapies should be used cautiously and patient should be closely observed: thiazides and other diuretics, corticosteroids, phenothiazines, thyroid products, estrogens, oral contraceptives, phenytoin, nicotinic acid, sympathomimetics, calcium channel blockers, and isoniazid.

Prior and concomitant therapies listed as prohibited in Table 6 are not permitted for the specific time frames listed. (See Appendix 7)

Intravenous Contrast

The European Society of Urogenital Radiology advocates stopping metformin 48 hours before CT for patients with an eGFR of less than 45 mL/min.

Patients with an eGFR above 60 mL/min who receive a larger amount of intravenous contrast (> 100 mL, e.g., CT of the abdomen or pelvis, CT angiography of the aorta or lower extremities) should restart metformin no earlier than 48 hours after the procedure.

7. Discontinuation of Study Intervention and Participant Discontinuation/Withdrawal

7.1. Discontinuation of Study Intervention

In some instances, it may be necessary for a participant to permanently discontinue study intervention. If study intervention is permanently discontinued, every effort should be made to keep the participant in the study for safety follow-up. See the SoA for data to be collected at the time of discontinuation of study intervention and follow-up and for any further evaluations that need to be completed.

At the baseline visit, patients will be given a list of precautions and reasons for temporarily or permanently discontinuing study medication, as well as a form of contact with the investigator.

7.1.1. Permanent Discontinuation

Study medication must be permanently discontinued in the following circumstances:

- Diabetes onset
- Hypersensitivity to the active substance or to any of the excipients.
- Unrecovered severe renal failure (eGFR <45 ml/min).
- Any acute metabolic acidosis event (such as lactic acidosis, diabetic ketoacidosis).
- Adverse events or intolerances related to the study medication, at the investigator's discretion.
- Diagnosis of B or C hepatitis infection or any other progressive liver disease.
- Diagnosis of chronic heart failure, or cancer.
- Alcohol abuse:
    - For men, heavy drinking is typically defined as consuming 15 drinks or more per week.
    - For women, heavy drinking is typically defined as consuming 8 drinks or more per week.
- Participants starting other maintenance medications that according to study drug label are contraindicated with metformin. (Appendix 7)
- Pregnancy.
- Any new events, comorbidities or medications that according to the investigator could entail additional risks for the participant.

7.1.2. Temporary Discontinuation

Study medication must be temporary discontinued in the following circumstances:

- Liver laboratory abnormalities: ALT over 5 times the upper limit of normal (ULN) or ALT over 3xULN and bilirubin over 1.5xULN or any verified Grade 4 laboratory abnormality that to the investigators criteria would affect the safety of the participant.

- Acute disorders that involve a risk of impaired kidney function such as dehydration, severe infection, or shock.
- Acute or chronic disease capable of causing tissue hypoxia, such as heart or respiratory failure, recent myocardial infarction, shock, or stroke.
- Hepatic failure, acute alcohol intoxication, alcoholism.
- Study medication should be stopped at the time of surgical intervention with general, spinal, or epidural anesthesia. Treatment can be restarted 48 hours after the intervention surgical procedure or after resumption of oral feeding, provided renal function has been reassessed and proven to be stable.
- Study medication should be stopped prior to or at the time of intravenous iodinated contrast test and should not be restarted until at least 48 hours, provided kidney function has been reassessed.
- Participants unable to swallow study medication tablets during the treatment period.
- Participants starting other temporary medications that according to study drug label are contraindicated with metformin. (Appendix 7)
- Any new events, comorbidities or medications that according to the investigator could entail additional risks for the participant.

7.1.3. Rechallenge

Patients with a permanent or temporary discontinuation will be evaluated by the investigator as soon as possible for safety evaluation and to assess the possibility of re-inclusion.

Rechallenge with the study medication after a temporary discontinuation may be considered at the discretion of the investigator, as long as the event has completely resolved, is not related to the study medication, and the patient agrees to continue the intervention.

In the event of transitory impaired renal function, study medication may be restarted after recovery of an estimated GFR > 45 mL/min.

If the cumulative duration of the interruptions is greater than two months, re-inclusion will not be considered, but study visits will continue as scheduled.

7.2. Participant Discontinuation/Withdrawal from the Study

- In accordance with the current revision of the Declaration of Helsinki (Fortaleza, Brazil October 2013) (World Medical Association. Declaration of Helsinki- Ethical principles for medical research involving human subjects. *World Med Assoc*. 2013) a participant may withdraw from the study at any time at his/her own request without prejudice to the medical care provided by his doctor or referral centre in the future; or may be withdrawn at any time at the discretion of the investigator for safety, behavioral, or compliance reasons. This is expected to be uncommon. Specific reasons for withdrawal:
    - Participant withdraws consent.

  - The participant is not willing to comply with the procedures required in the protocol.
  - For safety reasons, especially the occurrence of an event that requires permanent discontinuation of the study medication (see above).
  - Intolerance to the study medication.
  - Pregnancy.
  - Subject is incorrectly included in the study based due to a screening failure.
  - At the discretion of the investigator for any other safety, behavioral, or compliance reasons based on good clinical practice guidelines and clinical jugdment.
  - Lost to follow-up.
  - Transfer to another hospital does not necessarily imply withdrawal if the patient wishes to continue in the study and comply with the protocol.
  - A participant may be withdrawn from the study if any AE, SAE, or laboratory safety result occurs, regardless of its relationship to the study medication, that in the opinion of the investigator requires withdrawal because further treatment would jeopardize the safety of the participant.
- The reasons for participants not completing the study will be recorded in the case report form.
- At the time of discontinuing from the study, if possible, an early discontinuation visit should be conducted, as shown in the SoA. See SoA for data to be collected at the time of study discontinuation and follow-up and for any further evaluations that need to be completed.
- The participant will be permanently discontinued both from the study intervention and from the study at that time.
- If the participant withdraws consent for disclosure of future information, the sponsor may retain and continue to use any data collected before such a withdrawal of consent.
- If a participant withdraws from the study, he/she may request destruction of any samples taken and not tested, and the investigator must document this in the site study records.

7.3. Lost to Follow up

A participant will be considered lost to follow-up if he or she repeatedly fails to return for scheduled visits and is unable to be contacted by the study site.

The following actions must be taken if a participant fails to return to the clinic for a required study visit:

- The site must attempt to contact the participant and reschedule the missed visit as soon as possible and counsel the participant on the importance of maintaining the assigned visit

schedule and ascertain whether or not the participant wishes to and/or should continue in the study.

- Before a participant is deemed lost to follow up, the investigator or designee must make every effort to regain contact with the participant (where possible, telephone calls and, if necessary, a certified letter to the participant's last known mailing address or local equivalent methods). These contact attempts should be documented in the participant's medical record.
- Should the participant continue to be unreachable, he/she will be considered to have withdrawn from the study.

© 2015, 2019 TransCelerate BioPharma

8. Study Assessments and Procedures

- Study procedures and their timing are summarized in the SoA. Protocol waivers or exemptions are not allowed.
- Immediate safety concerns should be discussed with the sponsor immediately upon occurrence or awareness to determine if the participant should continue or discontinue study intervention.
- Adherence to the study design requirements, including those specified in the SoA, is essential and required for study conduct.
- All screening evaluations must be completed and reviewed to confirm that potential participants meet all eligibility criteria. The investigator will maintain a screening log to record details of all participants screened and to confirm eligibility or record reasons for screening failure, as applicable.
- Procedures conducted as part of the participant's routine clinical management (e.g., blood count) and obtained before signing of the ICF may be utilized for screening or baseline purposes provided the procedures met the protocol-specified criteria and were performed within the time frame defined in the SoA.
- Safety or laboratory results that could unblind the study will not be reported to blinded personnel until the study has been unblinded.
- Repeat or unscheduled samples may be taken for safety reasons or for technical issues with the samples.

8.1. Assessments

Planned time points for all primary and secondary assessments are provided in the SoA.

8.1.1. Visit Assessments

Screening Visit

Participants will receive the information relative to proceedings and study visits. If they agree to participate, written informed consent would be signed before any procedure. Screening assessments include: thorough medical history and complete physical exam, review of inclusion and exclusion criteria and laboratory assessment (Hematology, creatinine, eGFR, ALT, AST, fasting lipids, fasting glucose, HOMA-IR, Glycated hemoglobin (Hb A1c), $CD4^+$ $CD8^+$ cell count, HIV-1 RNA, FSH).

Visit 1 (Baseline)

Review of inclusion and exclusion criteria, concomitant medication, physical exam including anthropometric measurements (weight, height, waist circumference, blood pressure, and heart

rate), dispense study drug (metformin 850 mg once daily), dispense healthy lifestyle information, concomitant medication review, laboratory assessments (HOMA-IR , whole blood sample for epigenetic age acceleration measurements and leucocyte telomere length, lymphocyte and monocyte subpopulations, Inflammatory and procoagulant biomarkers, Blood TAME markers, oxidative stress and DNA damage markers), Frailty assessments (Fried index, grip strength, walking speed, short physical performance battery [SPPB]) and geriatric scales (mini-nutritional assessment and modified Rockwood Clinical Frailty Scale).

Visit 2 (Week 4 ± 2 days)

Concomitant medication, adverse events, adherence, symptom guided physical exam, limited safety laboratory panel (Hematology, renal and liver function, fasting glucose, urine), study drug titration (metformin placebo 850 mg twice daily) except those on dolutegravir based regimen.

Visit 3 (Week 8 ± 2 days)

This visit will be performed only in patients in whom the dose of metformin has been increased to 850mg dose twice daily in week 4.

Concomitant medication, adverse events, adherence, symptom guided physical exam, limited safety laboratory panel (Hematology, renal and liver function, fasting glucose, urine.

Visits 4, 6, 8 (Week 24 ± 7 days, week 72 ± 7 days, and week 120 ± 7 days)

Concomitant medication, adverse events, adherence, symptom guided physical exam, full safety laboratory panel (Hematology, renal and liver function, fasting glucose, fasting lipids, proteins, albumin, vitamin D, $CD4^+$ cell count, HIV-1 RNA, urine).

Visits 5, 7, 9 (Week 48 ± 7 days, week 96 ± 7 days, and week 144 ± 7 days)

Concomitant medication, adverse events, adherence, symptom guided physical exam, anthropometric measurements (weight, height, waist circumference, blood pressure, and heart rate), laboratory assessments (full safety laboratory panel, Hematology, renal and liver function, fasting glucose, fasting lipids, proteins, albumin, vitamin D, CD4+ and CD8+ cell count, HIV-1 RNA, urine, HOMA and HbA1c, whole blood sample for epigenetic age acceleration measurements and leucocyte telomere length, immune profile, inflammatory and pro-coagulant biomarkers, blood TAME markers, oxidative stress and DNA damage markers). Frailty testing: Fried index, grip strength, walking speed, short physical performance battery (SPPB) and geriatric scales (mini-nutritional assessment and modified Rockwood Clinical Frailty Scale).

Early discontinuation visit

At the time of discontinuing from the study, if possible, an early discontinuation visit should be conducted, as shown in the SoA. See SoA for data to be collected at the time of study discontinuation and follow-up and for any further evaluations that need to be completed (at least detail the participant's evolution, date of treatment discontinuation, and new prescribed therapy).

© 2015, 2019 TransCelerate BioPharma

8.2. Safety Assessments

Planned time points for all safety assessments are provided in the SoA.

8.2.1. Physical Examinations

- A complete physical examination will include, at a minimum, assessments of the Cardiovascular, Respiratory, Gastrointestinal and Neurological systems. Height and weight will also be measured and recorded.
- A brief physical examination will include, at a minimum, assessments of the skin, lungs, cardiovascular system, and abdomen (liver and spleen).
- Investigators should pay special attention to clinical signs related to previous serious illnesses.

8.2.2. Vital Signs

- Pulse rate, respiratory rate, blood pressure, height and weight will be assessed.
- Blood pressure and pulse measurements will be assessed with a completely automated device. Manual techniques will be used only if an automated device is not available.

Blood pressure and pulse measurements should be preceded by at least 5 minutes of rest for the participant in a quiet setting without distractions (e.g., television, cell phones) and before blood collection for laboratory tests.

8.2.3. Clinical Safety Laboratory Assessments

- See Appendix 2 for the list of clinical laboratory tests to be performed and to the SoA (Section 1.3) for the timing and frequency.
- The investigator must review the laboratory report, document this review, and record any clinically significant changes occurring during the study as an AE. The laboratory reports must be filed with the source documents.
- Abnormal laboratory findings associated with the underlying disease are not considered clinically significant unless judged by the investigator to be more severe than expected for the participant’s condition.
- All laboratory tests with values considered clinically significantly abnormal during participation in the study should be repeated until the values return to normal or baseline or are no longer considered clinically significant by the investigator or medical monitor.

© 2015, 2019 TransCelerate BioPharma

- If clinically significant values do not return to normal/baseline within a period of time judged reasonable by the investigator, the etiology should be identified, and the sponsor notified.
- All protocol-required laboratory tests, as defined in Appendix 2, must be conducted in accordance with the laboratory manual and the SoA (Section 1.3).
- If laboratory values from non-protocol specified laboratory tests performed at the institution's local laboratory require a change in participant management or are considered clinically significant by the investigator (e.g., SAE or AE or dose modification), then the results must be recorded.

8.3. Other study laboratory assessments

- Collection of biological samples for biomarker research is also part of this study. Blood samples for biomarker research are required and will be collected from all participants in this study as specified in the SoA.
- Samples will be tested for protocol-specific endpoints to evaluate their association with the study intervention.
- These blood laboratory assessments will include (See SoA and Appendix 2 for further information).
  - Laboratory tests for epigenetic age acceleration by DNA methylation arrays: Determination of epigenetic age acceleration according to four epigenetic clocks (Horvath´s clock, Hannum´s clock, PhenoAge and GrimAge).
  - Telomere length in PBMC. Genomic DNA from frozen PBMCs would be performed using the QIAamp DNA Blood Kit Mini Kit (Qiagen, Germany). Relative telomere length, expressed as ratio of telomere (T) to single-copy gene (S), would be determined by monochrome quantitative multiplex polymerase chain reaction (qPCR) assay, with minor modifications as described in our prior study(38) . Baseline and follow-up samples would be assayed in triplicate on the same PCR plate and those with a coefficient of variation (CV) greater than 10% would be reanalyzed.
  - Inmmunophenotype by flow cytometry. Fresh PBMCs appropriately stained would be acquired with a Beckman Coulter Navios cytometer and/or with a Celesta BD cytometer to quantify different lymphocyte subsets. Analysis of collected data would be performed with Flowlogic software. Flow Cytometry in fresh blood samples will analyse haematopoietic progenitors (CD34), Lymphocyte T ($CD3^+$) $CD4^+$ and $CD8^+$ [RTE (CD45RA, CD31), naïve ($CD27^+$, $CD45RA^+$), central memory ($CD27^+$, $CD45RA^-$), efector memory ($CD27^-CD45RA^-$), TEMRA ($CD27^-$, $CD45RA^+$), Activated ($CD38^+$, HLA DR^+), senescent cells ($CD28^-CD57^+$), exhaust ($PD1^+$)], Lymphocyte B ($CD19^+$) [(naïve ($CD27^-$, IgD^+), class-switched memory ($CD27^+$, IgD^-), non class-switched memory ($CD27^+$, IgD^+)], Natural killer (CD56dim CD16hi, CD56hi CD16-/low), T-regs ($CD25^+$, $FoxP3^+$), monocyte subpopulations (classic $CD14^{++}$ $CD16^-$, non-classic $CD14^{++}$ $CD16^{++}$, intermediate $CD14^+$ $CD16^+$).

- Blood TAME biomarkers (39) and other inflammatory and pro-coagulant biomarkers: IL-1 beta, TNF-alfa, D-dimer, IL-6, TNFα receptor, C-Reactive Protein, GDF15, IGF-1 C-Reactive Protein, Fasting insulin, Cystatin C, NT-proBNP. Cytokines and soluble markers would be determined in serum by solid phase ELISA quantikine human immunoassay (R&D system, Minnesota, USA). Baseline and follow-up samples would be assayed in duplicate on the same plate.
- DNA damage analysis by H2AX histone levels measurement: Nuclear protein extraction from frozen PBMCs would be performed using the CellLytic™ NuCLEAR™ Extraction Kit (Merck, Darmsdat, Germany). γH2AX levels would be determined by Western blot using Phospho-Histone H2A.X-P-Ser139 antibody 1:1000 (Cell Signaling Technology, Danvers, MA, USA) Blots would be washed and re-incubated with an Actin antibody 1:200 (Sigma-Aldrich, St. Louis, MI, USA), as loading control. Anti-rabbit IgG-HRP 1:2000 (Santa Cruz Biotechnology, Dallas, TX, USA) would be used as secondary antibody. Blots would be incubated with the Western Blotting Luminol Reagent (Santa Cruz Biotechnology Dallas, TX, USA). Protein levels would be analyzed with ImageJ. Baseline and follow-up samples would be assayed in duplicate on the same blot and repeated twice.
- Catalase, SOD1 and SOD2 levels: Total RNA would be obtained from frozen PBMCs. cDNAs would be generated from 2 µg of RNA and qPCR would be performed by triplicate using specific Taqman probes. Actin would be amplified as loading control. Relative gene expression quantification would be calculated according to the comparative threshold cycle method ($2^{-\Delta\Delta Ct}$) and normalized to the amplification signal obtained for PBMCs for each mRNA. Baseline and follow-up samples would be assayed on the same plate.
- ROS levels: One million of thawed PBMCs, previously cryopreserved, would be exposed to TBHP (tert-butyl hydroperoxide) as positive control or to N-acetilcystein as negative control or in absence (proper ROS levels). ROS levels would be determined using CellROX Green reagents (ThermoFisher, Massachussets, USA) and analyzed by flow cytometry. Baseline and follow-up samples would be assayed twice.
- Viral reservoir in PBMC and CD4 T-cell. Genomic DNA from frozen PBMCs and CD4 T-cells ($1x10^6$ cells and $5x10^6$ cells) would be performed using the QIAamp DNA Blood Kit Mini Kit (Qiagen, Germany).

  To measure total and integrated (Gag) HIV-1 DNA, nucleic acid would be quantified by nested real time PCR. All measurements were performed in triplicate. The first PCR would be performed by using primers to the human Alu and HIV-gag regions. The second PCR, real-time PCR, detects HIV-specific products by using primers to the R and U5 regions within the HIV long terminal repeat (LTR) and a specific fluorescent probe. In the case of integrated HIV DNA, the Alu primer serves as an anchor in the human genome and the gag primer serves as an anchor in the HIV genome. Binding sites for these two primers would be present in the DNA target template only when HIV has integrated into the human genome. Furthermore, when the primer binding sites are close enough and aligned correctly, the region between them can be amplified exponentially. Quantification would be achieved by

comparing the resultant signals to those obtained with an integration standard: ACH2 cells carrying one copy of integrated HIV-DNA per cell (NIH AIDS Reagent Program). To measure the background signal or the signal expected from unintegrated DNA, a control reaction using only the gag primer would be included in the first PCR. The signals from Alu-gag and gag-only would be compared to make sure the Alu-gag signal is stronger, and thus that the sample is positive for integration. Then a correlation would be developed that relates the PCR signals to proviral level, which is used to quantify the level of integration in the samples. To normalize the number of cells per sample, we would use a specific couple of primers and a fluorescent probe in a real-time PCR for CD3 amplification.

To measure CA US HIV-RNA, total RNA would be isolate from total CD4 + T-cells using RNeasy Mini Kit with on-column DNase treatment (Qiagen), according to the manufacturer´s instructions. The quality (260nm/280nm ratio) and quantity of RNA collected were evaluated by spectrophotometry on a Nanodrop instrument. Extracted cellular RNA would be treated with DNase (DNA-free kit; Thermo Fisher Scientific, Waltham, MA) to remove DNA that could interfere with quantitation and reverse transcribed using random primers and SuperScript III reverse transcriptase (Thermo Fisher Scientific). CA HIV-1 US RNA would be measured using seminested quantitative PCR (qPCR)-based assays. Retrotranscription would be performed with random hexamers as primers and SuperCript III (Invitrogen, Waltham, MA) according to the manufacturer´s instructions. Quantification of LTR-Gag HIV-RNA would be performed by using specific external primers for the first amplification and internal primers for the second amplification; and a fluorescent probe. US LTR-gag HIV-RNA standards were generated from plasmids by in vitro transcription (MEGAscript™ T7 Transcription Kit, ThermoFisher). The number of copies of each transcript was normalized to the levels of the reference gene beta glucuronidase (GUSB). All measurements were performed in triplicate.

To measure Intact proviral DNA assay (IPDA) genomic DNA would be extracted using the QIAamp DNA Mini Kit (Qiagen). DNA concentrations would be determined using a Nanodrop instrument. Quantification of intact, 5' deleted, and 3' deleted and/or hypermutated proviruses would be carried out using specific primer/probe combinations optimized for subtype B HIV-1 using the technique described by Bruner K et al (26) using droplet digital PCR. All measurements would be performed in triplicate.

- Peripheral Blood Mononuclear Cells (PBMCs) would be isolated by differential density gradient centrifugation from whole blood by exploiting differences in cell density. Granulocytes and erythrocytes have a higher density that mononuclear cells and therefore sediment during centrifugation. The PBMCs would be used freshly to determine the immunophenotype. The PBMC destined to telomere length measurement, DNA damage evaluation and catalase, SOD1, SOD2 mRNA expression levels would be frozen. To determine reactive oxygen species PBMCs would be cryopreserved.

- CD4 T-cell isolation from fresh PBMCs using the CD4 T cell isolation Kit, Human (Miltenyi, Alemania) by negative selection. Purity of CD4 T-cell isolation would be evaluate by flow cytometry. Samples would be stored at -80ºC.

8.4. Frailty Phenotype assessments and geriatric scale

Frailty Phenotype assessments:

- Frailty phenotype assessment is also part of this study and will be evaluated from all participants as specified in the SoA.
- Frailty phenotype will be assessed for protocol-specific endpoints to evaluate its association with the study intervention.
- Frailty phenotype assessment will include (See SoA and Appendix 5 for further information):
  - Fried frailty phenotype criteria (40):
    - Unintentional weight loss (5% or 4.5 Kg or more during the last year).
    - Exhaustion Using the responses (YES/NO) to two statements of the depression scale CES-D.
    - Physical activity assessed by the short version of the Minnesota Leisure Time Activity questionnaire
    - Gait speed on a 4.6 m (15 feet distance) adjusted for gender and height.
    - Dominant hand grip strength in Kg adjusted for gender and BMI.

    Patients will be classified as frail if they meet at least three of these criteria using the reference values of the Spanish population. Non-fragile patients will include pre-frail patients (those who meet 1 or 2 criteria) and fit patients (those without any of the criteria).
- Short Physical Performance Battery: The SPPB is based on three timed tasks: standing balance, walking speed, and chair stand tests. The timed results of each subtest are rescaled according to predefined cut-points for obtaining a score ranging from 0 (worst performance) to 12 (best performance).
- Hand-grip strength in the dominant hand, according to the protocol published by Mohd Hairi F et al. (41), and using the Jamar hydraulic hand dynamometer (Sammons Preston, Bolingbrook, IL, USA).

Geriatric scale:

- Mini-Nutritional Assessment. This includes a short questionnaire to screen for undernutrition in geriatric practice (44). Each answer provides a score and the sum of all

of them gives the screening score, ranging from 14 to 0 points, where 12-14 points represents a healthy nutritional state and 0-7 points represents malnutrition.

- Modified Rockwood Clinical Frailty Scale. This scale allows to determine the state of health deficit associated to ageing and in this study we used a modified version with 8 different frailty levels, ranging from a Fit state to a Very Severe Frailty State (45).
- Short Geriatric Depression Scale (Yesavage test).

8.5. Adverse Events (AEs), Serious Adverse Events (SAEs), and Other Safety Reporting

The definitions of adverse events (AEs) and serious adverse events (SAEs) can be found in Appendix 4.

AEs will be reported by the participant (or, when appropriate, by a caregiver, surrogate, or the participant's legally authorized representative).

The investigator and any qualified designees are responsible for detecting, documenting, and recording events that meet the definition of an AE or SAE and remain responsible for following up all Aes OR Aes that are serious, considered related to the study intervention or study procedures, or that caused the participant to discontinue the study intervention (see Section 7).

The method of recording, evaluating, and assessing causality of Aes and SAEs and the procedures for completing and transmitting SAE reports are provided in Appendix 4.

SAEs will be reported to the trial's pharmacovigilance manager: Irene García García, Head of Pharmacovigilance, Unidad de Ensayos Clínicos Hospital La Paz. Irene.ucicec@gmail.com. 917277558)

8.5.1. Time Period and Frequency for Collecting AE and SAE Information

All SAEs will be collected from the signing of the informed consent form until the last follow-up visit at the time points specified in the SoA (Section 1.3).

All AEs will be collected from the signing of the informed consent until the last follow-up visit at the time points specified in the SoA (Section 1.3).

Medical occurrences that begin before the start of study intervention but after obtaining informed consent will be recorded as Medical History/Current Medical Conditions, not as AEs.

All SAEs will be recorded and reported to the sponsor or designee immediately and under no circumstance should this exceed 24 hours, as indicated in Appendix 4. As indicated in Appendix 4, those disease/disorder being studied or expected progression, signs, or symptoms of the disease/disorder being studied, don´t meet the AE Definition, and therefore will not be considered SAEs if severe criteria are met, also not expedited reporting is needed. The investigator will submit any updated SAE data to the sponsor within 24 hours of it being available.

Investigators are not obligated to actively seek information on AEs or SAEs after conclusion of the study participation. However, if the investigator learns of any SAE, including a death, at any time after a participant has been discharged from the study, and he/she considers the event to be reasonably related to the study intervention or study participation, the investigator must promptly notify the sponsor.

8.5.2. Method of Detecting AEs and SAEs

Care will be taken not to introduce bias when detecting AEs and/or SAEs. Open-ended and non-leading verbal questioning of the participant is the preferred method to inquire about AE occurrences.

8.5.3. Follow-up of AEs and SAEs

After the initial AE/SAE report, the investigator is required to proactively follow each participant at subsequent visits/contacts. All SAEs will be followed until resolution, stabilization, the event is otherwise explained, or the participant is lost to follow-up. Further information on follow-up procedures is provided in Appendix 4.

8.5.4. Regulatory Reporting Requirements for SAEs

- Prompt notification by the investigator to the sponsor of an SAE is essential so that legal obligations and ethical responsibilities towards the safety of participants and the safety of a study intervention under clinical investigation are met.
- The sponsor has a legal responsibility to notify both the local regulatory authority and other regulatory agencies about the safety of a study intervention under clinical investigation. The sponsor will comply with country-specific regulatory requirements relating to safety reporting to the regulatory authority, Institutional Review Boards (IRB)/Independent Ethics Committees (IEC), and investigators.
- An investigator who receives an investigator safety report describing an SUSAR or other specific safety information (e.g., summary or listing of SAEs) from the sponsor will review and then file it along with the summary of product characteristics and will notify the IRB/IEC, if appropriate according to local requirements.

© 2015, 2019 TransCelerate BioPharma

9. Statistical Considerations

9.1. Statistical Hypotheses

This study is designed to show that the antiaging effect of starting metformin superior to placebo at week 96 in HIV-1 infected ART-experienced subjects.

9.2. Sample Size Determination

Approximately 80 participants will be screened to achieve 30 randomly assigned to study intervention for an estimated total of 30 evaluable participants per intervention group.

A maximum of 40 participants will be randomly assigned to study intervention such that approximately 30 evaluable participants complete the study.

Note: “Enrolled” means a participant’s, or their legally acceptable representative’s, agreement to participate in a clinical study following completion of the informed consent process. Potential participants who are screened for the purpose of determining eligibility for the study, but do not participate in the study, are not considered enrolled, unless otherwise specified by the protocol.

We do not have prior information to estimate the sample size accurately. With 30 subjects per group, a confidence level of 95% we would be able to detect with a power of 80% an effect size greater than 0.8.

Note: “Enrolled” means a participant’s, or their legally acceptable representative’s, agreement to participate in a clinical study following completion of the informed consent process and screening. Potential participants who are screened for the purpose of determining eligibility for the study, but do not participate in the study, are not considered enrolled, unless otherwise specified by the protocol.

9.3. Analysis Sets

For the purposes of analysis, the following analysis sets are defined:

Participant Analysis Set	Description
Randomized	Participants who were assigned to one group of study intervention
Evaluable	Participants who received at least 1 dose of study intervention
Safety	All randomized participants who are exposed to study intervention. Participants will be analyzed according to the intervention they actually received.

Defined Analysis Data Sets	Description
Analysis set for primary estimand: Intention-to-treat-exposed	All randomized participants who are exposed to study intervention. For participants who discontinue study intervention and/or receive rescue

	therapy, all post discontinuation or post rescue observations will be included in the analysis set.
Per protocol	All randomized participants. For participants who discontinue study intervention and/or receive rescue therapy, post discontinuation or post rescue observations will not be included

9.4. Statistical Analyses

The statistical analysis plan will be finalized prior to database lock and it will include a more technical and detailed description of the statistical analyses described in this section. This section is a summary of the planned statistical analyses of the most important endpoints including primary and key secondary endpoints.

9.4.1. General Considerations

Efficacy results that will be deemed to be statistically significant after consideration of the type I error control. To determine the treatment effect between study intervention groups, the estimate of the between-group treatment effect (with a nominal 95% CI) for the primary endpoint will be estimated and plotted within each group.

At least one laboratory value or vital sign obtained subsequent to at least one dose of study treatment is required for inclusion in the analysis of the respective safety parameter. To assess change from baseline, a baseline measurement is also required.

Two study periods are defined: intervention period (96 weeks since randomization) and follow-up phase (from week 96 to week 144)

9.4.2. Primary Endpoint(s)

Mixed models for repeated measures will be used to assess treatment differences of the change EAA measured by Phenoage at every visit. The repeated measures analysis will assume that the treatment difference can vary between visits (ie. treatment*visit interaction will be included in the model) and separates estimates and 95% confidence intervals will be produced at each visit. The model will also assume that the effect of baseline value for the endpoint can vary between visits (ie. baseline value*visit interaction will be included in the model) and will adjust for other potential confounders.

9.4.3. Secondary Endpoint(s)

Mixed models for repeated measures will be used to assess treatment differences of the secondary endpoint previously described.

9.4.4. Analysis

Safety and tolerability will be assessed by clinical review of all relevant parameters including AEs, events of clinical interest, laboratory values and vital signs.

For continuous measures such as changes from baseline in laboratory and vital signs parameters, summary statistics for baseline, on-treatment, and change from baseline values will be provided

by treatment group in table format. In addition, summary statistics for the difference between treatment groups will also be provided. Incidence and severity of adverse events and laboratory abnormalities will be compared by treatment group using χ-squared test.

© 2015, 2019 TransCelerate BioPharma

10. Supporting Documentation and Operational Considerations

10.1. Appendix 1: Regulatory, Ethical, and Study Oversight Considerations

10.1.1. Regulatory and Ethical Considerations

- This study will be conducted in accordance with the protocol and with the following:
  - Consensus ethical principles derived from international guidelines including the Declaration of Helsinki and Council for International Organizations of Medical Sciences (CIOMS) International Ethical Guidelines
  - Applicable ICH Good Clinical Practice (GCP) Guidelines
  - Applicable laws and regulations
- The protocol, protocol amendments, ICF, Investigator Brochure and other relevant documents (e.g., advertisements) must be submitted to an IRB/IEC by the investigator and reviewed and approved by the IRB/IEC before the study is initiated.
- Any amendments to the protocol will require IRB/IEC approval before implementation of changes made to the study design, except for changes necessary to eliminate an immediate hazard to study participants.
- Protocols and any substantial amendments to the protocol will require health authority approval prior to initiation except for changes necessary to eliminate an immediate hazard to study participants.
- The investigator will be responsible for the following:
  - Providing written summaries of the status of the study to the IRB/IEC annually or more frequently in accordance with the requirements, policies, and procedures established by the IRB/IEC
  - Notifying the IRB/IEC of SAEs or other significant safety findings as required by IRB/IEC procedures
  - Providing oversight of the conduct of the study at the site and adherence to requirements of 21 CFR, ICH guidelines, the IRB/IEC, European regulation 536/2014 for clinical studies (if applicable), European Medical Device Regulation 2017/745 for clinical device research (if applicable), and all other applicable local regulations

10.1.2. Financial Disclosure

Investigators and sub-investigators will provide the sponsor with sufficient, accurate financial information as requested to allow the sponsor to submit complete and accurate financial certification or disclosure statements to the appropriate regulatory authorities.

© 2015, 2019 TransCelerate BioPharma

10.1.3. Informed Consent Process

- The investigator or his/her representative will explain the nature of the study to the participant and answer all questions regarding the study.
- Participants must be informed that their participation is voluntary. Participants will be required to sign a statement of informed consent that meets the requirements of local regulations, ICH guidelines, Health Insurance Portability and Accountability Act (HIPAA) requirements, where applicable, and the IRB/IEC or study center.
- The medical record must include a statement that written informed consent was obtained before the participant was enrolled in the study and the date the written consent was obtained. The authorized person obtaining the informed consent must also sign the ICF.
- Participants must be re-consented to the most current version of the ICF(s) during their participation in the study.
- A copy of the ICF(s) must be provided to the participant
- A participant who is rescreened is not required to sign another ICF if the rescreening occurs within (30) days from the previous ICF signature date.

The ICF will contain a separate section that addresses the use of remaining mandatory samples for optional exploratory research. The investigator or authorized designee will explain to each participant the objectives of the exploratory research. Participants will be told that they are free to refuse to participate and may withdraw their consent at any time and for any reason during the storage period. A separate signature will be required to document a participant's agreement to allow any remaining specimens to be used for exploratory research. Participants who decline to participate in this optional research will not provide this separate signature.

10.1.4. Data Protection

The study sponsor and investigators will guarantee the confidentiality of the subjects' data and ensure compliance at all times with current legislation (General Regulation of Data Protection – Reglamento General de Protección de datos (RGPD) 679/2016).

- Participants will be assigned a unique identifier by the sponsor. Any participant records or datasets that are transferred to the sponsor will contain the identifier only; participant names or any information which would make the participant identifiable will not be transferred.
- The participant must be informed that his/her personal study-related data will be used by the sponsor in accordance with local data protection law. The level of disclosure must also be explained to the participant who will be required to give consent for their data to be used as described in the informed consent

- The participant must be informed that his/her medical records may be examined by Clinical Quality Assurance auditors or other authorized personnel appointed by the sponsor, by appropriate IRB/IEC members, and by inspectors from regulatory authorities.

10.1.5. Committees Structure

10.1.5.1 Early Safety Data Committee

- Participant safety will be continuously monitored by the Sponsor's internal committee, which includes safety signal detection at any time during the study
- In addition, an early aggregated safety data review will be performed, the goal of which is to allow for a cautious, stepwise approach to drug administration. An initial safety review for this study is planned for the first 30 participants (50% of participants) who are dosed and have provided safety data for 28 days after administration of Dose 1
- All safety data collected will be summarized and reviewed by the Sponsor's internal committee for agreement of next steps.
- In particular, data will be reviewed by the Sponsor for identification of the following events that would potentially contribute to a requirement to stop the study.
  - Any deaths, regardless of causality
  - Grade 3 AEs reported in more than 2 participants (see table in Appendix 4)
- Enrollment will be paused during the review. If a stopping] rule is met, a decision will be made, based on the review, as to whether enrollment in the study will be allowed to resume.
- Case unblinding may be performed for above reviews if necessary.

10.1.6. Dissemination of Clinical Study Data

Study information from this protocol will be posted on publicly available clinical trial registers before enrollment of subjects begins.

10.1.7. Data Quality Assurance

- All participant data relating to the study will be recorded on printed or electronic CRF unless transmitted to the sponsor or designee electronically (e.g., laboratory data). The investigator is responsible for verifying that data entries are accurate and correct by physically or electronically signing the CRF.
- Guidance on completion of CRFs will be provided in the Trial Master File.
- The investigator must permit study-related monitoring, audits, IRB/IEC review, and regulatory agency inspections and provide direct access to source data documents.

- Monitoring details describing strategy, including definition of study critical data items and processes (eg, risk-based initiatives in operations and quality such as risk management and mitigation strategies and analytical risk-based monitoring), methods, responsibilities, and requirements, including handling of noncompliance issues and monitoring techniques (central, remote, or on-site monitoring) are provided in the monitoring plan.
- Monitoring details describing strategy (e.g., risk-based initiatives in operations and quality such as Risk Management and Mitigation Strategies and Analytical Risk-Based Monitoring), methods, responsibilities and requirements, including handling of noncompliance issues and monitoring techniques (central, remote, or on-site monitoring) are provided in the Monitoring Plan.
- The sponsor or designee is responsible for the data management of this study including quality checking of the data.
- The sponsor assumes accountability for actions delegated to other individuals (e.g., Contract Research Organizations).
- Records and documents, including signed ICFs, pertaining to the conduct of this study must be retained by the investigator for 10 years after study completion unless local regulations or institutional policies require a longer retention period. No records may be destroyed during the retention period without the written approval of the sponsor. No records may be transferred to another location or party without written notification to the sponsor.

10.1.8. Source Documents

- Source documents provide evidence for the existence of the participant and substantiate the integrity of the data collected. Source documents are filed at the investigator’s site.
- Data reported on the CRF or entered in the eCRF that are transcribed from source documents must be consistent with the source documents or the discrepancies must be explained. The investigator may need to request previous medical records or transfer records, depending on the study. Also, current medical records must be available.
- The investigator must maintain accurate documentation (source data) that supports the information entered in the CRF.
- Study monitors will perform ongoing source data verification to confirm that data entered into the CRF by authorized site personnel are accurate, complete, and verifiable from source documents; that the safety and rights of participants are being protected; and that the study is being conducted in accordance with the currently approved protocol and any other study agreements, ICH GCP, and all applicable regulatory requirements.

10.1.9. Study and Site Start and Closure

First Act of Recruitment

The study start date is the date on which the clinical study will be open for recruitment of participants.

Study/Site Termination

The sponsor or designee reserves the right to close the study site or terminate the study at any time for any reason at the sole discretion of the sponsor. Study sites will be closed upon study completion. A study site is considered closed when all required documents and study supplies have been collected and a study-site closure visit has been performed.

The investigator may initiate study-site closure at any time, provided there is reasonable cause and sufficient notice is given in advance of the intended termination.

Reasons for the early closure of a study site by the sponsor or investigator may include but are not limited to:

For study termination:

- Discontinuation of further study intervention development

For site termination:

- Failure of the investigator to comply with the protocol, the requirements of the IRB/IEC or local health authorities, the sponsor's procedures, or GCP guidelines
- Inadequate or no recruitment (evaluated after a reasonable amount of time) of participants by the investigator
- Total number of participants included earlier than expected

If the study is prematurely terminated or suspended, the sponsor shall promptly inform the investigators, the IECs/IRBs, the regulatory authorities, and any contract research organization(s) used in the study of the reason for termination or suspension, as specified by the applicable regulatory requirements. The investigator shall promptly inform the participant and should assure appropriate participant therapy and/or follow-up

10.1.10. Publication Policy

- The results of this study may be published or presented at scientific meetings. If this is foreseen, the investigator agrees to submit all manuscripts or abstracts to the sponsor before submission. This allows the sponsor to protect proprietary information and to provide comments.
- The sponsor will comply with the requirements for publication of study results. In accordance with standard editorial and ethical practice, the sponsor will generally support

publication of multicenter studies only in their entirety and not as individual site data. In this case, a coordinating investigator will be designated by mutual agreement.

- Authorship will be determined by mutual agreement and in line with International Committee of Medical Journal Editors authorship requirements.

10.2. Appendix 2: Clinical Laboratory Tests

- The tests detailed in Table 2 will be performed by the local laboratory at La Paz University Hospital.
- The tests detailed in Table 3 will be performed by the local laboratory at IdiPaz.
- Table 4 specifies the collection tubes for each visit.
- Protocol-specific requirements for inclusion or exclusion of participants are detailed in Section 5 of the protocol.
- Investigators must document their review of each laboratory safety report.
- Additional tests may be performed at any time during the study as determined necessary by the investigator or required by local regulations.

Table 2: Protocol-Required Safety Laboratory Tests

<table>
<tr><th>Laboratory Tests</th><th colspan="4">Parameters</th></tr>
<tr><td rowspan="5">Hematology</td><td>Platelet Count</td><td rowspan="5" colspan="2">RBC Indices:
MCV
MCH
% Reticulocytes</td><td rowspan="5">White blood cell (WBC) count with Differential:
Neutrophils
Lymphocytes
Monocytes
Eosinophils
Basophils</td></tr>
<tr><td>Red blood cell (RBC) Count</td></tr>
<tr><td>Hemoglobin</td></tr>
<tr><td>Hematocrit</td></tr>
<tr><td>$CD4^+$ and $CD8^+$ cell count</td></tr>
<tr><td rowspan="3">Clinical Chemistry[1]</td><td>Blood urea nitrogen (BUN)</td><td>Potassium</td><td>Aspartate Aminotransferase (AST)</td><td>Total and direct bilirubin</td></tr>
<tr><td>Creatinine</td><td>Sodium</td><td>Alanine Aminotransferase (ALT)</td><td>Total Protein
Albumine</td></tr>
<tr><td>Fasting Glucose</td><td>Calcium</td><td>Alkaline phosphatase[2]</td><td>Vitamin D</td></tr>
<tr><td>Routine Urinalysis</td><td colspan="4">• Specific gravity
• pH, glucose, protein, blood, ketones, bilirubin, urobilinogen, nitrite, leukocyte esterase by dipstick
• Microscopic examination (if blood or protein is abnormal)</td></tr>
<tr><td>Other Screening Tests</td><td colspan="4">• Serology: hepatitis B surface antigen (HBsAg), and hepatitis C virus antibody (or RNA test in the case of a previous positive antibody test)
• HIV-1 Viral load.</td></tr>
</table>

© 2015, 2019 TransCelerate BioPharma

	• B12 vitamin levels.<br>• Glycated hemoglobin (A1C).<br>• IGF-1<br>• Fasting insulin<br>• HOMA-IR<br>• D-dimer<br>• Cystatin C<br>• NT-proBNP
NOTES:<br>[1] Details of liver chemistry stopping criteria and required actions and follow-up are given in Section [7.1.1 Liver Chemistry Stopping Criteria] and Appendix [6: Liver Safety: Suggested Actions and Follow-up Assessments [and Study Intervention Rechallenge Guidelines]]. All events of ALT [or AST] ≥3 × upper limit of normal (ULN) and total bilirubin ≥2 × ULN (>35% direct bilirubin) or ALT [or AST] ≥3 × ULN and international normalized ratio (INR) >1.5, (if INR measured) which may indicate severe liver injury (possible Hy's Law), must be reported to [sponsor] in an expedited manner (excluding studies of hepatic impairment or cirrhosis).<br>[2] If alkaline phosphatase is elevated, consider fractionating.<br>[3] Local urine testing will be standard for the protocol unless serum testing is required by local regulation or IRB/IEC.	

Table 3: Protocol-Required Laboratory Tests at IdiPaz laboratory.

Laboratory Tests	Parameters	
Laboratory tests for epigenetic age acceleration	Infinium methylation EPIC arrays (Illumina): Determination of epigenetic age acceleration according to four epigenetic clocks (Horvath´s clock, Hannum´s clock, PhenoAge and GrimAge).	
Immune profile	Lymphocyte and monocyte subpopulations	Fresh PBMCs appropriately stained would be acquired with a Beckman Coulter Navios cytometer and/or with a Celesta BD cytometer to quantify different lymphocyte subsets. Analysis of collected data would be performed with Flowlogic software. Flow Cytometry in fresh blood samples will analyse haematopoietic progenitors ($CD34^{+}$), Lymphocyte T ($CD3^{+}$) $CD4^{+}$ and $CD8^{+}$ [RTE ($CD45RA^{+}$, $CD31^{+}$), naïve ($CD27^{+}$, $CD45RA^{+}$), central memory ($CD27^{+}$, $CD45RA^{-}$), efector memory ($CD27^{-}CD45RA^{-}$), TEMRA ($CD27^{-}$, $CD45RA^{+}$), Activated ($CD38^{+}$, $HLA\text{-}DR^{+}$), senescent cells ($CD28\text{-}CD57^{+}$), exhaust ($PD1^{+}$)], Lymphocyte B ($CD19^{+}$) [(naïve ($CD27^{-}$, IgD^{+}), class-switched memory ($CD27^{+}$, IgD^{-}), non class-switched memory ($CD27^{+}$, IgD^{+})], Natural killer (CD56dim CD16hi, CD56hi CD16-/low), T-regs ($CD25^{+}$, $FoxP3^{+}$), monocyte subpopulations (classic $CD14^{++}$ CD16-, non-classic $CD14^{++}$ $CD16^{++}$, intermediate $CD14^{+}$ $CD16^{+}$).
	Other inflammatory and pro-coagulant biomarkers[2]	IL-1 beta, TNF-alfa.

	Blood TAME biomarkers[2]	IL-6, TNFα receptor, GDF15.<br>*Other TAME biomarkers (Fasting insulin, cystatin C, NT-proBNP, haemoglobin A1c, IGF-1) will be measured in the laboratory of La Paz University Hospital.
Oxidative stress and DNA damage markers	DNA damage analysis by H2AX histone levels measurement[3], Reactive oxygen species (ROS) [4], catalase expression, superoxide dismutase 1- 2 levels[5].	
Leucocyte telomere length measurement by qPCR	Genomic DNA from frozen PBMCs would be performed using the QIAamp DNA Blood Kit Mini Kit (250) (Qiagen, Germany). Relative telomere length, expressed as ratio of telomere (T) to single-copy gene (S), would be determined by monochrome quantitative multiplex polymerase chain reaction (PCR) assay, with minor modifications as described in our prior study Baseline and follow-up samples would be assayed in triplicate on the same PCR plate and those with a coefficient of variation (CV) greater than 10% would be reanalyzed. (Montejano R, Stella-Ascariz N, Monge S, et al. Impact of antiretroviral treatment containing tenofovir difumarate on the telomere length of aviremic HIV-infected patients. J Acquir Immune Defic Syndr 2017; 76:102–9)	
Viral reservoir	Genomic DNA and RNA from frozen PBMCs and CD4 T-cell would be isolated and quantified by qPCR and droplet-PCR.	

[1]Peripheral Blood Mononuclear Cells (PBMCs) would be isolated by differential density gradient centrifugation from whole blood by exploiting differences in cell density. Granulocytes and erythrocytes have a higher density that mononuclear cells and therefore sediment during centrifugation. The PBMCs would be used freshly to determine the immunophenotype. The PBMC destined to telomere length measurement, DNA damage evaluation and catalase, SOD1, SOD2 mRNA expression levels would be frozen. To determine reactive oxygen species PBMCs would be cryopreserved.

2 Cytokines and soluble markers would be determined in serum by solid phase ELISA quantikine human immunoassay (R&D system, Minnesota, USA). Baseline and follow-up samples would be assayed in duplicate on the same plate.

3 Nuclear protein extraction from frozen PBMCs would be performed using the CellLytic™ NuCLEAR™ Extraction Kit (Merck, Darmsdat, Germany). γH2AX levels would be determined by Western blot using Phospho-Histone H2A.X-P-Ser139 antibody 1:1000 (Cell Signaling Technology, Danvers, MA, USA) Blots would be washed and re-incubated with an Actin antibody 1:200 (Sigma-Aldrich, St. Louis, MI, USA), as loading control. Anti-rabbit IgG-HRP 1:2000 (Santa Cruz Biotechnology, Dallas, TX, USA) would be used as secondary antibody. Blots would be incubated with the Western Blotting Luminol Reagent (Santa Cruz Biotechnology Dallas, TX, USA). Protein levels would be analyzed with ImageJ. Baseline and follow-up samples would be assayed in duplicate on the same blot and repeated twice.

4 One million of thawed PBMCs, previously cryopreserved, would be exposed to TBHP (tert-butyl hydroperoxide) as positive control or to N-acetilcystein as negative control or in absence (proper ROS levels). ROS levels would be determined using CellROX Green reagents (ThermoFisher, Massachussets, USA) and analyzed by flow cytometry. Baseline and follow-up samples would be assayed twice.

5 Catalase, SOD1 and SOD2 levels: Total RNA would be obtained from frozen PBMCs. cDNAs would be generated from 2 µg of RNA and qPCR would be performed by triplicate using specific Taqman probes. Actin would be amplified as loading control. Relative gene expression quantification would be calculated according to the comparative threshold cycle method ($2^{-\Delta\Delta Ct}$) and normalized to the amplification signal obtained for PBMCs for each mRNA. Baseline and follow-up samples would be assayed on the same plate.

Table 4. Collection flow chart.

Collection tubes		Screening (up to 30 days before Day 0)	Intervention Period, Visit number [Week]									E/D
			1 [0]	2 [4]	3 [8]	4 [24]	5 [48]	6 [72]	7 [96]	8 [120]	9 [144]	
1 EDTA 3 ml tube. (ref.454020) 1 serum 5 ml tube. (ref. 456018) (La Paz University Hospital laboratory)	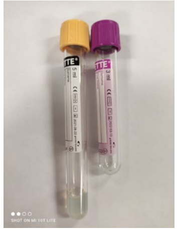	X	X	X	X	X	X	X	X	X	X	X
1 coagulation 3 ml tube. (ref. 454334) (La Paz University Hospital laboratory)	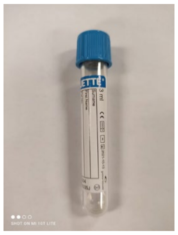	X	X			X	X	X	X	X	X	
1 EDTA 3 ml tube. (ref. 454020) 1 EDTA 5 ml tube. (ref.456058) (La Paz University Hospital laboratory)	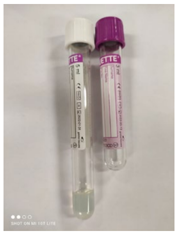	X	X			X	X	X	X	X	X	X
1 serum 8 ml tube. (ref.455071) (La Paz University Hospital laboratory)	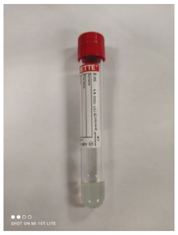	X				X	X	X	X	X	X	
Urinalysis (La Paz University Hospital laboratory)	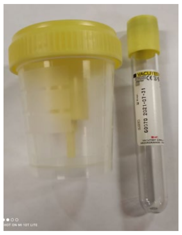	X	X	X	X	X	X	X	X	X	X	X

Collection tubes		Screening (up to 30 days before Day 0)	Intervention Period, Visit number [Week]									E/D
			1 [0]	2 [4]	3 [8]	4 [24]	5 [48]	6 [72]	7 [96]	8 [120]	9 [144]	
4 EDTA 9 ml tubes (ref. 455036)<br>1 serum 8 ml tube (ref.455071)<br>(IdiPaz laboratory)	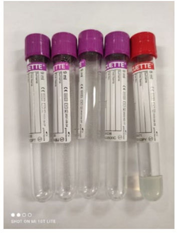		X				X		X		X	

10.3. Appendix 3: Frailty Phenotype Assessment and geriatric scale

10.3.1. Fried frailty phenotype criteria

Patients will be classified as frail if they meet at least three of these criteria using the reference values of the Spanish population. Non-fragile patients will include pre-frail patients (those who meet 1 or 2 criteria) and fit patients (those without any of the criteria).(40)

- Unintentional weight loss (5% or 4.5 Kg or more during the last year).
- Exhaustion Using the responses (YES/NO) to two statements of the depression scale CES-D.
- Physical activity assessed by the short version of the Minnesota Leisure Time Activity questionnaire.
- Gait speed on a 4.6 m (15 feet distance) adjusted for gender and height. See section 10.3.3
- Dominant hand grip strength in Kg adjusted for gender and BMI. See section 10.3.4

Tabla 5. Fried frailty phenotype criteria.

**1. Pérdida de peso**
Pérdida de peso no intencionada en el último año mayor de 10 libras (4,5 kg) o mayor del 5% del peso previo en el último año.
**2. Baja energía y resistencia**
Respuesta afirmativa a cualquiera de la dos preguntas de la escala de depresión CES-D: "¿Sentía que todo lo que hacía suponía un esfuerzo en la última semana?" o "¿Sentía que no podía ponerse en marcha la última semana?".<br>Se considera criterio de fragilidad si a una de ellas se responde: "moderada cantidad de tiempo (3-4 días) o la mayor parte del tiempo".
**3. Bajo nivel de actividad física**
Kilocalorías gastadas por semana usando el *Minnesota Leisure Time Activity Questionnaire* (MLTAQ), estratificado por género.<br>Se considera fragilidad cuando se encuentra en el quintil inferior:<br>Hombres < 383 kcal/semana<br>Mujeres < 270 kcal/semana
**4. Velocidad de la marcha**
Tiempo que se tarda en andar 15 pasos (4,6 m), estratificado por altura y género.<br>Se considera criterio de fragilidad cuando los sujetos están en el quintil inferior:<br>Hombres: altura ≤ 173 cm ≥ 7 segundos/altura > 173 cm ≥ 6 segundos<br>Mujeres: altura ≤ 159 cm ≥ 7 segundos/altura > 159 cm ≥ 6 segundos
**5. Fuerza prensora**
Medida en kilogramos y estratificada por género e índice de masa corporal.<br>Se considera criterio de fragilidad cuando los sujetos están en el quintil inferior:

Hombres:		Mujeres:	
IMC ≤ 24	≤ 29	IMC ≤ 23	≤ 17
IMC 24,1-26	≤ 30	IMC 23,1-26	≤ 17,3
IMC 26,1-28	≤ 30	IMC 26,1-29	≤ 18
IMC > 28	≤ 32	IMC > 29	≤ 21

Se considera frágiles a los sujetos que cumplen tres o más criterios, prefrágiles a los que cumplen uno o dos y no frágiles o robustos a los que no cumplen ninguno.
Recientemente se han validado valores normativos de velocidad de la marcha y fuerza prensora en población española.

Source: Guía de Buena práctica clínica en Geriatría. Fragilidad y nutrición en el anciano. Available on the internet: https://www.segg.es/media/descargas/GBPCG_Fragilidad_y_nutricion_en_el_anciano.pdf

Tabla 6. Minnesota Leisure Time Activity Questionnaire (MLTAQ):

LISTA DE ACTIVIDADES FISICAS

(Marque con una cruz la casilla correspondiente a las actividades físicas que haya realizado durante el último año)

Andar - Bailar - Subir escaleras

|__| 1 Pasear
|__| 2 Andar de casa al trabajo y del trabajo a casa o durante el periodo de descanso del trabajo
|__| 3 Andar (llevando carrito de la compra)
|__| 4 Andar (llevando bolsas de la compra)
|__| 5 Subir escaleras
|__| 6 Andar campo a través
|__| 7 Excursiones con mochila
|__| 8 Escalar montañas
|__| 9 Ir en bicicleta al trabajo
|__|10 Bailar
|__|11 Aerobic o ballet
|__|12 Jugar con los niños (corriendo, saltando,..)

Ejercicios de mantenimiento general

|__|13 Hacer ejercicio en casa
|__|14 Hacer ejercicio en un gimnasio
|__|15 Caminar deprisa
|__|16 Trotar ("Jogging")
|__|17 Correr 8-11 km/h
|__|18 Correr 12-16 km/h
|__|19 Levantar pesas

Actividades acuáticas

|__|20 Esquí acuático
|__|21 Surf
|__|22 Navegar a vela
|__|23 Ir en canoa o remar (por distracción)
|__|24 Ir en canoa o remar (en competición)
|__|25 Hacer un viaje en canoa
|__|26 Nadar (más de 150 metros en piscina)
|__|27 Nadar en el mar
|__|28 Bucear

Deportes de invierno

|__|29 Esquiar
|__|30 Esqui de fondo
|__|31 Patinar (ruedas o hielo)

Otras actividades

|__|32 Montar a caballo
|__|33 Jugar a los bolos
|__|34 Balonvolea
|__|35 Tenis de mesa
|__|36 Tenis individual
|__|37 Tenis dobles
|__|38 Badminton
|__|39 Baloncesto (sin jugar partido)
|__|40 Baloncesto (jugando un partido)
|__|41 Baloncesto (actuando de árbitro)
|__|42 Squash
|__|43 Fútbol
|__|44 Golf (llevando el carrito)
|__|45 Golf (andando y llevando los palos)
|__|46 Balonmano
|__|47 Petanca
|__|48 Artes marciales
|__|49 Motociclismo
|__|50 Ciclismo de carretera o montaña

Actividades en el jardín

|__|51 Cortar el césped con máquina
|__|52 Cortar el césped manualmente
|__|53 Limpiar y arreglar el jardín
|__|54 Cavar el huerto
|__|55 Quitar nieve con pala

Trabajos y actividades caseras

|__|56 Trabajos de carpintería dentro de casa
|__|57 Trabajos de carpinteria (exterior)
|__|58 Pintar dentro de casa
|__|59 Pintar fuera de casa
|__|60 Limpiar la casa
|__|61 Mover muebles

Caza y pesca

|__|62 Tiro con pistola
|__|63 Tiro con arco
|__|64 Pescar en la orilla del mar
|__|65 Pescar con botas altas dentro del río
|__|66 Caza menor
|__|67 Caza mayor (ciervos, osos...)

Otras (Especificar)
|__|68 ....
|__|69 ....

LISTADO DE ACTIVIDADES FISICAS CON SU CODIGO DE INTENSIDAD

ACTIVIDAD FISICA	METS
1 Pasear	3.5
2 Andar de casa al trabajo y del trabajo a casa o durante el periodo de descanso en el trabajo	4.0
3 Andar (llevando el carrito de la compra)	3.5
4 Andar (llevando bolsas de la compra)	5.5
5 Subir escaleras	8.0
6 Andar campo a traves (excursiones)	6.0
7 Excursiones con mochila	7.0
8 Escalar montañas	8.0
9 Ir en bicicleta al trabajo o pasear	4.0
10 Bailar	4.5
11 Aerobic o ballet	6.0
12 Jugar con los niños (corriendo, saltando,...)	4.5
13 Hacer ejercicio en casa	4.5
14 Hacer ejercicio en un gimnasio	6.0
15 Caminar deprisa	4.5
16 Trotar ("Jogging")	6.0
17 Correr 8-11 km/h	10.0
18 Correr 12-16 km/h	15.0
19 Levantar pesas	6.0
20 Esquí acuático	6.0
21 Surf	6.0
22 Navegar a vela	3.0
23 Ir en canoa o remar (por distracción)	3.5
24 Ir en canoa o remar (en competición)	12.0
25 Hacer un viaje en canoa	4.0
26 Nadar (más de 150 metros en piscina)	6.0
27 Nadar en el mar	6.0
28 Bucear	5.0
29 Esquiar	7.0
30 Esqui de fondo	8.0
31 Patinar (ruedas o hielo)	7.0
32 Montar a caballo	5.0
33 Jugar a los bolos	3.0
34 Balonvolea	4.0
35 Tenis de mesa	4.0
36 Tenis individual	8.0
37 Tenis dobles	6.0
38 Badminton	7.0
39 Baloncesto (sin jugar partido)	6.0
40 Baloncesto (jugando un partido)	8.0
41 Baloncesto (actuando de árbitro)	7.0
42 Squash	12.0

43	Futbol	10.0
44	Golf (llevando el carrito)	3.5
45	Golf (andando y llevando los los palos)	5.5
46	Balonmano	10.0
47	Petanca	3.0
48	Artes marciales	10.0
49	Motociclismo	4.0
50	Ciclismo de carretera o montaña	9.0
51	Cortar el césped con máquina	4.5
52	Cortar el césped manualmente	6.0
53	Limpiar y arreglar el jardín	4.5
54	Cavar el huerto	5.0
55	Quitar nieve con pala	6.0
56	Trabajos de carpinteria dentro del taller	3.0
57	Trabajos de carpintería (exterior).	6.0
58	Pintar dentro de casa (incluye empapelar)	4.5
59	Pintar fuera de casa	5.0
60	Limpiar la casa	3.5
61	Mover muebles	6.0
62	Tiro con pistola	2.5
63	Tiro con arco	3.5
64	Pescar en la orilla del mar	3.5
65	Pescar con botas altas dentro del rio	6.0
66	Caza menor	5.0
67	Caza mayor	6.0

ENCUESTA DE ACTIVIDAD FISICA EN EL TIEMPO LIBRE DE MINNESOTA

Nombre |__|__|__|__|__|__|__|__|__|__|__|__|__|__|__|__|__|__|__|__|__|__|__|__|__|__|__|__|__|__|__|__|__|__|__|__|__|

Apellidos |__|__|__|__|__|__|__|__|__|__|__|__|__|__|__|__|__|__|__|__|__|__|__|__|__|__|__|__|__|__|__|__|__|__|__|__|__| Identificador |__|__|__|__|

ULTIMA SEMANA

Código de Actividad Física	Dias de práctica última semana	Minutos de práctica/día

ULTIMO MES

Código de Actividad Física	Dias de práctica último mes	Minutos de práctica/día

© 2015, 2019 TransCelerate BioPharma

ENCUESTA DE ACTIVIDAD FISICA EN EL TIEMPO LIBRE DE MINNESOTA

Nombre |__|__|__|__|__|__|__|__|__|__|__|__|__|__|__|__|__|__|__|__|__|__|__|__|__|__|__|__|__|__|__|__|__|__|__|__|__|

Apellidos |__|__|__|__|__|__|__|__|__|__|__|__|__|__|__|__|__|__|__|__|__|__|__|__|__|__|__|__|__|__|__|__|__|__|__|__|__| Identificador |__|__|__|__|

ULTIMO TRIMESTRE

Código de Actividad Física	Dias de práctica último trimestre	Minutos de práctica/día

ULTIMO AÑO

Código de Actividad Física	Dias de práctica último año	Minutos de práctica/día

10.3.2. Short Physical Performance Battery

It consists of timing three tests: standing balance (in three positions: feet together, semi-tandem and tandem), walking speed (about 2.4 or 4 meters) and getting up and sitting in a chair five times. It is important to respect the sequence of the tests, since if we start with the raised, the patient can become fatigued and give falsely low performances on the other two subtests.

The timed results of each subtest are rescaled according to predefined cut-points for obtaining a score ranging from 0 (worst performance) to 12 (best performance). The normative values for the Spanish population have been established in various studies of population cohorts and primary care. The score and assessment of the total result of the SPPB results from the sum of the three sub-tests, and ranges from 0 (worst) to 12; changes in 1 point have clinical significance. A score below 10 indicates frailty and a high risk of disability, as well as falls. The time to carry out this test with training is 6 to 10 minutes.

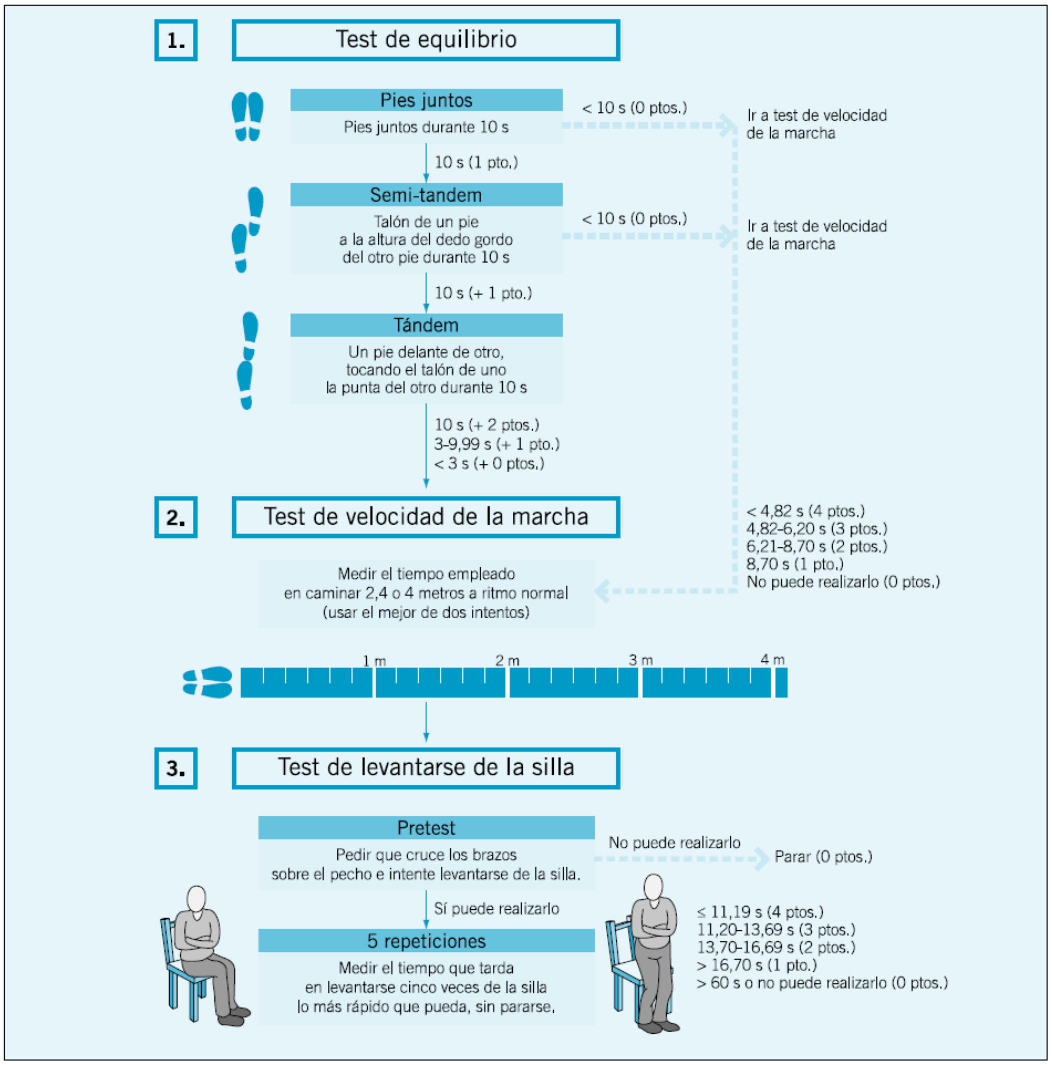

Source: Guía de Buena práctica clínica en Geriatría. Fragilidad y nutrición en el anciano. Available on the internet: https://www.segg.es/media/descargas/GBPCG_Fragilidad_y_nutricion_en_el_anciano.pdf

10.3.3. Gait speed

It consists of asking the person to walk 2.4; 4; 5; 6 or 10 m distance at his/her usual walking pace. The 4-meter test is the best validated in the literature. The most used threshold for frailty is less than 0.8 m/sec. In the case of speeds greater than 4 meters, there are interpretability data that indicate 0.05 m/s as the smallest significant change and 0.1 m/s as the substantial change. The time to perform the test varies from 2 to 3 minutes.

10.3.4. Hand-grip strength

Hand-grip strength in the dominant hand measured in kilograms using the Jamar hydraulic hand dynamometer (Sammons Preston, Bolingbrook, IL, USA) according to the modified Southampton protocol published by Roberts HC et al. (41)

The position in which the subject performs the test is a determining factor in the validity of the test. The participant must be seated, with a straight back, upper arm tight against the trunk, elbow flexed at 90º without support, and forearm and wrist in neutral rotation. The inner lever of the dynamometer must be adjusted to suit the hand. Participants will be instructed to squeeze as hard as possible for a few seconds.

Two measurements will be made, and the highest will be chosen. Measurements will be considered valid if the two measurements of one hand differ by less than 20 kg. Values of zero or those above 100 kg will be considered invalid. 23 kg and 13 kg cut-off value for male and female respectively, will be used according to previous studies performed in our sanitary area. (42)

10.3.5. Geriatric scale

MINI-NUTRITIONAL ASSESMENT

Have they lost their appetite? Has food intake declined due to loss of appetite, digestive problems, chewing or swallowing difficulties over the past three months?

- Severe loss of appetite
- Moderate loss of appetite
- No loss of appetite

Recent weight loss

- Weight loss > 3kg
- Does not know
- Weight loss between 1 and 3 kg
- No weight loss

Mobility

- Bed or chair bound
- Able to get by at their home
- Goes out

Has suffered acute disease or psychological stress in the past three months?

- Yes
- No

Neuropsychological problems

- o Severe dementia or depression
- o Mild dementia
- o No psychological problems

Body Mass Index (BMI) = weight in kg/ / height in m2

- o BMI < 19
- o 19 ≤ BMI < 20
- o 21 ≤ BMI < 23
- o BMI ≥ 23

Screening score (subtotal ma. 14 points):
12-14 points: normal nutrition state. 8-11 points: at risk of malnutrition. 0-7 points: malnutrition.

Adapted from Rubenstein et al., (2001). See bibliography (44).

MODIFIED ROCKWOOD CLINICAL FRAILTY SCALE

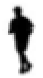	**1**	**Fit**	People who are robust, active, energetic and motivated. They tend to exercise regularly and are among the fittest for their age.
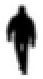	**2**	**Good Health Status**	People who have no serious disease nor chronic disease symptoms but exercise less than the former category. They play adequate sports occasionally, depending on the moment.
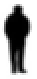	**3**	**Managing Well**	People whose medical problems are well controlled, but often are not regularly active beyond routine walking.
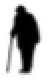	**4**	**Vulnerable**	They do not need other people's assistance in order to perform their daily basic tasks; however, their symptoms often limit their activities. A common complaint is being "slowed up" or being tired during the day.
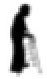	**5**	**Mild Frailty**	People who often have an evident slow pace and that require assistance to perform instrumental activities in their daily life (finances, transportation, heavy housework, drug intake). They progressively show more impairment walking outside alone, shopping, preparing meals and performing housework activities.
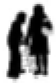	**6**	**Moderate Frailty**	People who need help with all outside activities and household chores. They often need help climbing stairs and taking a shower. They might need minimal assistance with dressing.
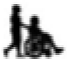	**7**	**Severe Frailty**	Completely dependent for personal care, due to either a physical or cognitive limitation. They seem stable and not at high risk of dying within 6 months.
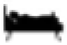	**8**	**Very Severe Frailty**	Completely dependent for personal care and approaching end of life. They could not recover even from a minor illness.

Adapted from https://www.dal.ca/sites/gmr/our-tools/clinical-frailty-scale.html and Rockwood K, et al. (202), see bibliography (45).

Short Geriatric Depression Scale

**1.**	Are you basically satisfied with your life?	yes	**no**
**2.**	Have you dropped many of your activities and interests?	**yes**	no
**3.**	Do you feel that your life is empty?	**yes**	no
**4.**	Do you often get bored?	**yes**	no
**5.**	Are you in good spirits most of the time?	yes	**no**
**6.**	Are you afraid that something bad is going to happen to you?	**yes**	no
**7.**	Do you feel happy most of the time?	yes	**no**
**8.**	Do you often feel helpless?	**yes**	no
**9.**	Do you prefer to stay at home, rather than going out and doing things?	**yes**	no
**10.**	Do you feel that you have more problems with memory than most?	**yes**	no
**11.**	Do you think it is wonderful to be alive now?	yes	**no**
**12.**	Do you feel worthless the way you are now?	**yes**	no
**13.**	Do you feel full of energy?	yes	**no**
**14.**	Do you feel that your situation is hopeless?	**yes**	no
**15.**	Do you think that most people are better off than you are?	**yes**	no

A score of ≥ 5 suggests depression **Total Score** ____________

Ref. Yes average: The use of Rating Depression Series in the Elderly, in Poon (ed.): Clinical Memory Assessment of Older Adults. American Psychological Association, 1986

2 Tools may be copied without permission

10.4. Appendix 4: AEs and SAEs: Definitions and Procedures for Recording, Evaluating, Follow-up, and Reporting

10.4.1. Definition of AE

AE Definition
• An AE is any untoward medical occurrence in a clinical study participant, temporally associated with the use of study intervention, whether or not considered related to the study intervention.<br>• NOTE: An AE can therefore be any unfavorable and unintended sign (including an abnormal laboratory finding), symptom, or disease (new or exacerbated) temporally associated with the use of study intervention.
**Definition of Unsolicited and Solicited AE**
• An unsolicited adverse event is an adverse event that was not solicited using a Participant Diary and that is communicated by a participant who has signed the informed consent. Unsolicited AEs include serious and non-serious AEs.<br>• Potential unsolicited AEs may be medically attended (i.e., symptoms or illnesses requiring a hospitalisation, or emergency room visit, or visit to/by a health care provider). The participant will be instructed to contact the site as soon as possible to report medically attended event(s), as well as any events that, though not medically attended, are of participant concern. Detailed information about reported unsolicited AEs will be collected by qualified site personnel and documented in the participant's records.<br>• Unsolicited AEs that are not medically attended nor perceived as a concern by participant will be collected during interview with the participant and by review of available medical records at the next visit.<br>• Solicited AEs are predefined local and systemic events for which the participant is specifically questioned, and which are noted by the participant in their diary.

Events Meeting the AE Definition
• Any abnormal laboratory test results (hematology, clinical chemistry, or urinalysis) or other safety assessments (e.g., ECG, radiological scans, vital signs measurements), including those that worsen from baseline, considered clinically significant in the medical and scientific judgment of the investigator (i.e., not related to progression of underlying disease).<br>• Exacerbation of a chronic or intermittent pre-existing condition including either an increase in frequency and/or intensity of the condition.

• New conditions detected or diagnosed after study intervention administration even though it may have been present before the start of the study.<br>• Signs, symptoms, or the clinical sequelae of a suspected intervention- intervention interaction.<br>• Signs, symptoms, or the clinical sequelae of a suspected overdose of either study intervention or a concomitant medication. Overdose per se will not be reported as an AE/SAE unless it is an intentional overdose taken with possible suicidal/self-harming intent. Such overdoses should be reported regardless of sequelae.<br>• The signs, symptoms, and/or clinical sequelae resulting from lack of efficacy will be reported as AE or SAE if they fulfill the definition of an AE or SAE. “Lack of efficacy” or “failure of expected pharmacological action” also constitutes an AE or SAE.

**Events NOT Meeting the AE Definition**
• Any clinically significant abnormal laboratory findings or other abnormal safety assessments which are associated with the underlying disease, unless judged by the investigator to be more severe than expected for the participant’s condition.<br>• The disease/disorder being studied or expected progression, signs, or symptoms of the disease/disorder being studied, unless more severe than expected for the participant’s condition.<br>• Medical or surgical procedure (e.g., endoscopy, appendectomy): the condition that leads to the procedure is the AE.<br>• Situations in which an untoward medical occurrence did not occur (social and/or convenience admission to a hospital).<br>• Anticipated day-to-day fluctuations of pre-existing disease(s) or condition(s) present or detected at the start of the study that do not worsen.

10.4.2. Definition of SAE

**An SAE is defined as any serious adverse event that, at any dose:**
**a. Results in death**
**b. Is life-threatening**<br>The term 'life-threatening' in the definition of 'serious' refers to an event in which the participant was at risk of death at the time of the event. It does not refer to an event, which hypothetically might have caused death, if it were more severe.
**c. Requires inpatient hospitalization or prolongation of existing hospitalization**

• In general, hospitalization signifies that the participant has been admitted (usually involving at least an overnight stay) at the hospital or emergency ward for observation and/or treatment that would not have been appropriate in the physician's office or outpatient setting. Complications that occur during hospitalization are AEs. If a complication prolongs hospitalization or fulfills any other serious criteria, the event is serious. When in doubt as to whether "hospitalization" occurred or was necessary, the AE should be considered serious.<br>• Hospitalization for elective treatment of a pre-existing condition that did not worsen from baseline is not considered an AE.
**d. Results in persistent or significant disability/incapacity**
• The term disability means a substantial disruption of a person's ability to conduct normal life functions.<br>• This definition is not intended to include experiences of relatively minor medical significance such as uncomplicated headache, nausea, vomiting, diarrhea, influenza, and accidental trauma (e.g., sprained ankle) which may interfere with or prevent everyday life functions but do not constitute a substantial disruption.
**e. Is a congenital anomaly/birth defect**
**f. Other situations:**
• Medical or scientific judgment should be exercised by the investigator in deciding whether SAE reporting is appropriate in other situations such as significant medical events that may jeopardize the participant or may require medical or surgical intervention to prevent one of the other outcomes listed in the above definition. These events should usually be considered serious.<br>o Examples of such events include invasive or malignant cancers, intensive treatment for allergic bronchospasm, blood dyscrasias, convulsions or development of intervention dependency or intervention abuse.

10.4.3. Recording and Follow-Up of AE and/or SAE

**AE and SAE Recording**
• When an AE/SAE occurs, it is the responsibility of the investigator to review all documentation (e.g., hospital progress notes, laboratory reports, and diagnostics reports) related to the event.<br>• The investigator will then record all relevant AE/SAE information.<br>• It is **not** acceptable for the investigator to send photocopies of the participant's medical records to the Sponsor in lieu of completion of the required form.<br>• There may be instances when copies of medical records for certain cases are requested by the Sponsor. In this case, all participant identifiers, with the exception of the

participant number, will be redacted on the copies of the medical records before submission to the Sponsor.

- The investigator will attempt to establish a diagnosis of the event based on signs, symptoms, and/or other clinical information. Whenever possible, the diagnosis (not the individual signs/symptoms) will be documented as the AE/SAE.

Assessment of Intensity

The investigator will make an assessment of intensity for each AE and SAE reported during the study

- The intensity of the AE will be graded following the DAIDS grading table for Severity of AEs.(43)
- AE severity grading scale ranging from grades 1 to 5 with descriptions for each AE based on the following general guidelines:
    - Grade 1 indicates a mild event (An event that is easily tolerated by the participant, causing minimal discomfort and not interfering with everyday activities)
    - Grade 2 indicates a moderate event (An event that causes sufficient discomfort to interfere with normal everyday activities.)
    - Grade 3 indicates a severe event (An event that prevents normal everyday activities. An AE that is assessed as severe should not be confused with an SAE. Severe is a category utilized for rating the intensity of an event; and both AEs and SAEs can be assessed as severe.

      An event is defined as ‘serious’ when it meets at least 1 of the predefined outcomes as described in the definition of an SAE, NOT when it is rated as severe.
    - Grade 4 indicates a potentially life-threatening event
    - Grade 5 indicates death
- If the severity of an AE could fall in either one of two grades (i.e., the severity of an AE could be either grade 2 or grade 3), sites should select the higher of the two grades.

Assessment of Causality

- The investigator is obligated to assess the relationship between study intervention and each occurrence of each AE/SAE.
- A “reasonable possibility” of a relationship conveys that there are facts, evidence, and/or arguments to suggest a causal relationship, rather than a relationship cannot be ruled out.

- The investigator will use clinical judgment to determine the relationship.
- Alternative causes, such as underlying disease(s), concomitant therapy, and other risk factors, as well as the temporal relationship of the event to study intervention administration will be considered and investigated.
- The investigator will also consult the Investigator's Brochure (IB) and/or Product Information, for marketed products, in his/her assessment.
- For each AE/SAE, the investigator **must** document in the medical notes that he/she has reviewed the AE/SAE and has provided an assessment of causality.
- There may be situations in which an SAE has occurred, and the investigator has minimal information to include in the initial report to medical monitor. However, it is very important that the investigator always make an assessment of causality for every event before the initial transmission of the SAE data to medical monitor.
- The investigator may change his/her opinion of causality in light of follow-up information and send an SAE follow-up report with the updated causality assessment.
- The causality assessment is one of the criteria used when determining regulatory reporting requirements.

Follow-up of AEs and SAEs
• The investigator is obligated to perform or arrange for the conduct of supplemental measurements and/or evaluations as medically indicated or as requested by the Sponsor to elucidate the nature and/or causality of the AE or SAE as fully as possible. This may include additional laboratory tests or investigations, histopathological examinations, or consultation with other health care professionals.<br>• New or updated information will be recorded in the originally submitted documents.<br>• The investigator will submit any updated SAE data to medical monitor within 24 hours of receipt of the information.

10.4.4. Reporting of SAEs

SAE Reporting via Paper Data Collection Tool
• The sponsor, or whoever assumes the tasks delegated by the sponsor, will notify the Spanish Agency of Medicines and Medical Devices (AEMPS), the ethics committees (CEIm) and the health authorities of the Autonomous Community of Madrid of all suspected SAEs in accordance with the current regulations on clinical trials within a maximum period of fifteen calendar days from the moment they become aware of the same. When the SAEs have caused the death or endangered the life of the patient, the notification will be made within a maximum period of seven calendar days from the

moment of knowledge of the same. Relevant information regarding subsequent events shall be supplemented within eight days.

- Facsimile transmission of the SAE paper data collection tool is the preferred method to transmit this information to the SAE coordinator.
- In rare circumstances and in the absence of facsimile equipment, notification by telephone is acceptable with a copy of the SAE data collection tool sent by overnight mail or courier service.
- Initial notification via telephone does not replace the need for the investigator to complete and sign the SAE data collection tool within the designated reporting time frames.
- Contacts for SAE reporting can be found in 8.3 section.

© 2015, 2019 TransCelerate BioPharma

10.5. Appendix 5: Contraceptive and Barrier Guidance

10.5.1. Definitions

Women of Childbearing Potential (WOCBP)

A woman is considered fertile following menarche and until becoming postmenopausal unless permanently sterile (see below):
If fertility is unclear (eg, amenorrhea in adolescents or athletes) and a menstrual cycle cannot be confirmed before first dose of study intervention, additional evaluation should be considered.
Women in the following categories are not considered WOCBP:

- Premenarchal
- Premenopausal female with 1 of the following:
    - Documented hysterectomy
    - Documented bilateral salpingectomy
    - Documented bilateral oophorectomy

For individuals with permanent infertility due to an alternate medical cause other than the above (eg, Mullerian agenesis, androgen insensitivity), investigator discretion should be applied to determining study entry.
Note: Documentation can come from the site personnel's review of the participant's medical records, medical examination, or medical history interview.

- Postmenopausal female

A postmenopausal state is defined as no menses for 12 months without an alternative medical cause. Peri- or post-menopausal status will be determined for candidates who have had the uterus removed by an assessment of blood follicle stimulating hormone (FSH). Women with an FSH level higher than 30 mlU/mL will be eligible for the study. See participant libraries for common text to include here.

10.6. Appendix 6: Genetics

Use/Analysis of DNA

- Genetic variation may impact a participant's response to study intervention, susceptibility to, and severity and progression of disease. Variable response to study intervention may be due to genetic determinants that impact intervention absorption, distribution, metabolism, and excretion; mechanism of action of the intervention; disease etiology; and/or molecular subtype of the disease being treated. Therefore, where local regulations and IRB/IEC allow, a blood sample will be collected for DNA analysis from consenting participants.
- DNA samples will be used for research related to epigenetic,telomere and viral reservoir measurement.
- DNA samples will be analyzed for epigenetic,telomere and viral reservoir measurement. Additional analyses may be conducted if it is hypothesized that this may help further understand the clinical data.
- The results of genetic analyses may be reported in the clinical study report (CSR) or in a separate study summary.
- The sponsor will store the DNA samples in a secure storage space with adequate measures to protect confidentiality.
- The samples will be retained while research on study intervention continues but no longer than 10 years or other period as per local requirements.

© 2015, 2019 TransCelerate BioPharma

10.7. Appendix 7: Prohibited concomitant medications

Table 6. Medications not allowed during the study.

Family of drugs	Drug	Interaction mechanism	Commentary
Androgens.	Fluoxymesterone, Mesterolone, Methyltestoterone, Nandrolone, Oxandrolone, Oxymetholone, Testosterone (Exception Danazol).	Changes in insulin sensitivity.	Androgens may enhance the hypoglycemic effect of Agents with Blood Glucose Lowering Effects, including metformin.
Antiadrenergic drugs.	Guanethidine.	Unclear.	Guanethidine product labeling states that because prolonged treatment with guanethidine may lower blood glucose concentrations, it may be necessary to adjust the dosage of insulin or oral anti-diabetic drugs when combined with guanethidine.
Antianginal therapy.	Ranolazine.	Inhibition of organic cation transporters (OCTs) responsible for metformin elimination.	Ranolazine may inhibit OCTs and increase the serum concentration of Metformin, potential for increased metformin concentrations and toxicity (including lactic acidosis).
Antiarrhythmic agents.	Dofetilide.	Inhibition of organic cation transporters (OCTs) responsible for metformin elimination.	Both dofetilide and metformin undergo cationic secretion, creating a theoretical possibility for competitive inhibition.
Anticholinergic Agents.	Glycopyrrolate.	Inhibit gastrointestinal motility and secretions.	The specific mechanism for this purported interaction is uncertain, but it may be related to the ability of glycopyrrolate to inhibit gastrointestinal motility and secretions, potential for increased metformin concentrations and toxicity (including lactic acidosis).

Family of drugs	Drug	Interaction mechanism	Commentary
Anticonvulsants.	Lamotrigine.	Inhibition of organic cation transporter 2 (OCT2)/SLC22A2 transporters responsible for metformin elimination.	Lamotrigine may inhibit OCTs, particularly OCT2, and increase the serum concentration of Metformin, potential for increased metformin concentrations and toxicity (including lactic acidosis).
	Topiramate.	Topiramate may induce acidosis.	Combination of metformin with topiramate may facilitate the development of lactic acidosis.
Antihistamines.	Cimetidine.	Inhibition of organic cation transporters (OCTs) responsible for metformin elimination.	Cimetidine may inhibit OCTs, particularly OCT2, and increase the serum concentration of Metformin, potential for increased metformin concentrations and toxicity (including lactic acidosis).
Antineoplastic Agents.	Erdafitinib, crizotinib, olaparib.	Inhibition of organic cation transporters (OCTs) responsible for metformin elimination.	These antineoplasic agents may inhibit OCTs, particularly OCT2, and increase the serum concentration of Metformin, potential for increased metformin concentrations and toxicity (including lactic acidosis).
	Abemaciclib, Vandetanib.	Inhibition of organic cation transporter 2 (OCT2) or multidrug and toxin extrusion (MATE) transporters responsible for metformin elimination.	These antineoplasic agents may inhibit OCT2, MATE1 and MATE2K, and increase the serum concentration of Metformin, potential for increased metformin concentrations and toxicity (including lactic acidosis).
	Tucatinib.	Inhibition of multidrug and toxin extrusion-1 (MATE-1) transporters responsible for metformin elimination.	These antineoplasic agents may inhibit MATE and increase the serum concentration of Metformin, potential for increased metformin concentrations and toxicity (including lactic acidosis).

© 2015, 2019 TransCelerate BioPharma

Family of drugs	Drug	Interaction mechanism	Commentary
Blocker of potassium channels that act on the nervous system.	Dalfampridine.	Inhibition of organic cation transporters (OCTs) responsible for metformin elimination.	Dalfampridine may inhibit OCTs and increase the serum concentration of Metformin, potential for increased metformin concentrations and toxicity (including lactic acidosis).
Carbonic Anhydrase Inhibitors.	Acetazolamide, Dichlorphenamide, Methazolamide, Sulthiame, Topiramate, Zonisamide<br>(Exceptions Brinzolamide, Dorzolamide).	Decrease serum bicarbonate and cause non-anion gap hypochloremic metabolic acidosis.	Carbonic anhydrase inhibitors may decrease serum bicarbonate and cause non-anion gap hypochloremic metabolic acidosis, thereby increasing the risk of development of lactic acidosis during metformin treatment.
Herbal medications.	Maitake.	Maitake may enhance the hypoglycemic effect of metformin.	The combination is not recommended during the study.
Monoamine Oxidase Inhibitors.	Isocarboxazid, Linezolid, Methylene Blue, Moclobemide, Phenelzine, Rasagiline, Safinamide, Selegiline, Tranylcypromine	Unclear, monoamine Oxidase Inhibitors may enhance the hypoglycemic effect of metformin.	The combination is not recommended during the study.
Nutritional supplements.	Alpha-Lipoic Acid.	Unclear.	Alpha-Lipoic Acid may enhance the hypoglycemic effect of Antidiabetic Agents, including metformin.
Potassium Binder Antidotes.	Patiromer.	Binding to metformin in the gastrointestinal tract and diminishing its absorption.	Patiromer may decrease the serum concentration of metformin.
Survival of Motor Neuron 2 (SMN2)-Directed RNA Splicing Modifier.	Risdiplam.	Inhibition of multidrug and toxin extrusion (MATE) 1 and MATE2-K transporters responsible for metformin elimination.	Risdiplam may inhibit MATE transporters and increase the serum concentration of Metformin, potential for increased metformin concentrations and toxicity (including lactic acidosis).

Table 7. Medications that require close monitoring of adverse events or dose adjustment during the study due to potential drug-interactions with metformin. Temporary discontinuation of metformin may be required.

Family of drugs	Drug	Interaction mechanism	Commentary
Angiotensin-Converting Enzyme Inhibitors.	Alacepril, Benazepril, Captopril, Cilazapril, Enalapril, Enalaprilat, Fosinopril, Imidapril, Lisinopril, Moexipril, Perindopril, Quinapril, Ramipril, Trandolapril, Zofenopril.	Uncertain.	Several studies have concluded that ACE inhibitors may improve insulin sensitivity, but others have reported no change in insulin sensitivity. The degree to which ACE inhibitors contribute to an increased risk of hypoglycemia or lactic acidosis is unclear. No additional precautions need to be taken during the study.
		Kidney failure.	Renal dysfunction that may be caused by Angiotensin-Converting Enzyme Inhibitors may lead to metformin-associated toxicities and lactic acidosis. Close laboratory and clinical monitoring (eg, renal function, signs, and symptoms of lactic acidosis) is indicated when administering metformin-containing medications with Angiotensin-Converting Enzyme Inhibitors at the investigator's clinical discretion.
Angiotensin II Receptor Blocker.	Azilsartan, Candesartan, Eprosartan, Fimasartan, Irbesartan, Losartan, Olmesartan, Telmisartan, Valsartan.	Kidney failure.	Renal dysfunction that may be caused by Angiotensin II Receptor Blocker may lead to metformin-associated toxicities and lactic acidosis. Close laboratory and clinical monitoring (eg, renal function, signs, and symptoms of lactic acidosis) is indicated when administering metformin-containing medications with Angiotensin II Receptor Blocker at the investigator's clinical discretion.
	Telmisartan.	Uncertain, telmisartan may inhibit the	Telmisartan may decrease the effect of metformin. No additional precautions need to be taken during the study.

Family of drugs	Drug	Interaction mechanism	Commentary
		absorption of metformin.	
Antidiabetic Agents.	Acarbose, Alogliptin, Bromocriptine, Canagliflozin, Dapagliflozin, Dulaglutide, Empagliflozin, Ertugliflozin, Evogliptin, Exenatide, Gemigliptin, Gliclazide, Glimepiride, Glipizide, Glyburide, Insulin Aspart, Insulin Degludec, Insulin Detemir, Insulin Glargine, Insulin Glulisine, Insulin Lispro, Insulin NPH, Insulin Regular, Ipragliflozin, Linagliptin, Liraglutide, Lixisenatide, Lobeglitazone, Miglitol, Mitiglinide, Nateglinide, Pioglitazone, Pramlintide, Repaglinide, Saxagliptin, Semaglutide, Sitagliptin, Teneligliptin, Tolazamide, Vildagliptin, Voglibose.	Antidiabetic Agents may enhance the hypoglycemic effect of metformin.	Monitor patients closely for hypoglycemic effects if these agents are combined at the investigator's clinical discretion.
Anticholinergic Agents.	Trospium.	Unclear, metformin may decrease the serum concentration of Trospium.	Monitor patients closely for evidence of reduced trospium effectiveness if used together with metformin.
Antiemetics.	Ondansetron.	Inhibition of organic cation transporters (OCTs) or multidrug and toxin extrusion (MATE) transporters	The mechanism of this potential interaction has not been fully investigated, but ondansetron inhibition of OCTs and/or MATE1, transporters involved in metformin elimination, likely contributes. Temporary

Family of drugs	Drug	Interaction mechanism	Commentary
		responsible for metformin elimination.	discontinuation of metformin is required until treatment with Ondansetron has been completed.
Antifungals.	Isavuconazole.	Inhibition of organic cation transporters (OCTs) or multidrug and toxin extrusion (MATE) transporters responsible for metformin elimination.	The mechanism of this potential interaction has not been fully investigated, but isavuconazole inhibition of OCT1, OCT2, and/or MATE1, transporters involved in metformin elimination, likely contributes. Temporary discontinuation of metformin is required until treatment with isavuconazole has been completed.
Antimalarials.	Tafenoquine.	Inhibition of organic cation transporter 2 (OCT2) or multidrug and toxin extrusion (MATE) transporters responsible for metformin elimination.	Tafenoquine may inhibit OCT2, MATE1 and MATE2K, and increase the serum concentration of Metformin, potential for increased metformin concentrations and toxicity (including lactic acidosis). Temporary discontinuation of metformin is required until treatment with tafenoquine has been completed.
Antimicrobials.	Cephalexin, Trimethoprim-sulfamethoxazole (co-trimoxazole).	Inhibition of organic cation transporter (OCT) transporters responsible for metformin elimination.	These antimicrobials may inhibit OCT and increase the serum concentration of Metformin, potential for increased metformin concentrations and toxicity (including lactic acidosis). Temporary discontinuation of metformin is required until treatment with these antimicrobials has been completed.
	Prothionamide	Prothionamide may enhance the hypoglycemic effect of metformin.	Monitor patients closely for hypoglycemic effects if these agents are combined at the investigator's clinical discretion.
	Quinolones: Ciprofloxacin (Systemic), Delafloxacin,	Quinolones may enhance the	Monitor patients closely for hypoglycemic effects if these agents are combined at the investigator's

Family of drugs	Drug	Interaction mechanism	Commentary
	Enoxacin, Gemifloxacin, Levofloxacin (Oral, Systemic), Lomefloxacin, Moxifloxacin (Systemic), Nalidixic Acid, Norfloxacin, Ofloxacin (Systemic), Pefloxacin, Pipemidic Acid, Sparfloxacin, Zabofloxacin.	hypoglycemic effect of metformin.	clinical discretion. Temporary discontinuation of metformin may be Required until treatment with quinolones has been completed.
	Rifampin.	Induction of organic cation transporter 1 (OCT1).	Rifampin may increase the absorption of metformin. Temporary discontinuation of metformin may be required until treatment with rifampin has been completed.
Diuretics.	Bumetanide, Ethacrynic Acid, Furosemide, Torsemide, Bendroflumethiazide, Chlorothiazide, Chlorthalidone, Cyclopenthiazide, Hydrochlorothiazide, Hydroflumethiazide, Indapamide, Methyclothiazide, Metolazone, Xipamide.	Kidney failure.	Renal dysfunction that may be caused by Diuretics may lead to metformin-associated toxicities and lactic acidosis. Close laboratory and clinical monitoring (eg, renal function, signs, and symptoms of lactic acidosis) is indicated when administering metformin-containing medications with Diuretics at the investigator's clinical discretion.
Growth Hormone Receptor Antagonists.	Pegvisomant.	Pegvisomant may enhance the hypoglycemic effect of metformin.	Monitor patients closely for hypoglycemic effects if these agents are combined at the investigator's clinical discretion.
HIV antiretrovirals.	Dolutegravir.	Inhibition of organic cation transporter 2 (OCT2) or multidrug and toxin extrusion (MATE) transporters	Dolutegravir may inhibit OCT2 and MATE1, and increase the serum concentration of Metformin, potential for increased metformin concentrations and toxicity (including lactic acidosis).

Family of drugs	Drug	Interaction mechanism	Commentary
		responsible for metformin elimination.	Metformin dose adjustment per protocol to once daily.
Iodinated Contrast Agents.	Iodinated Contrast Agents.	Kidney failure.	Renal dysfunction that may be caused by iodinated contrast agents may lead to metformin-associated toxicities and lactic acidosis. Temporary discontinuation of metformin is needed. Metformin Should be stopped prior to or at the time of intravenous iodinated contrast test and should not be restarted until at least 48 hours, provided kidney function has been reassessed.
Nonsteroidal Anti-Inflammatory Agents.	Aceclofenac, Acemetacin, Celecoxib, Dexibuprofen, Dexketoprofen, Diclofenac (Systemic), Diflunisal, Dipyrone, Etodolac, Etoricoxib, Fenoprofen, Flurbiprofen (Systemic), Ibuprofen, Indomethacin, Ketoprofen, Ketorolac (Nasal), Ketorolac (Systemic), Lornoxicam, Loxoprofen, Meclofenamate, Mefenamic Acid, Meloxicam, Morniflumate, Nabumetone, Naproxen, Nimesulide, Oxaprozin, Parecoxib, Pelubiprofen, Phenylbutazone, Piroxicam (Systemic), Propyphenazone, Sulindac, Talniflumate, Tenoxicam, Tiaprofenic	Kidney failure.	Renal dysfunction that may be caused by Nonsteroidal Anti-Inflammatory Agents may lead to metformin-associated toxicities and lactic acidosis. Close laboratory and clinical monitoring (eg, renal function, signs, and symptoms of lactic acidosis) is indicated when administering metformin-containing medications with NSAIDs at the investigator's clinical discretion, depending on the dose and duration of treatment with NSAIDs.

Family of drugs	Drug	Interaction mechanism	Commentary
	Acid, Tolfenamic Acid, Tolmetin, Zaltoprofen		
Selective Serotonin Reuptake Inhibitors.	Citalopram, Dapoxetine, Escitalopram, Fluoxetine, Fluvoxamine, Paroxetine, Sertraline, Vilazodone, Vortioxetine.	Unclear. Selective Serotonin Reuptake Inhibitors may enhance the hypoglycemic effect of metformin.	Monitor patients closely for hypoglycemic effects if these agents are combined at the investigator's clinical discretion.
Vitamin K antagonists.	Acenocoumarol, Phenindione, Phenprocoumon, Warfarin.	Unclear.	Metformin may diminish the anticoagulant effect of Vitamin K Antagonists. Monitor patients closely for anticoagulation effect if these Agents are combined at the investigator's clinical discretion. Vitamin K Antagonists may enhance the hypoglycemic effect of Metformin. Monitor patients closely for hypoglycemic effects if these agents are combined at the investigator's clinical discretion.

10.8. Appendix 8: Lifestyle recommendations

Hospital Universitario La Paz
Hospital de Cantoblanco
Hospital Carlos III
SaludMadrid
Comunidad de Madrid

Estudio METforAging

HOJA DE INFORMACIÓN ESTILO DE VIDA SALUDABLE Y EJERCICIO FÍSICO

¿POR QUÉ ES IMPORTANTE CUIDAR LA ALIMENTACIÓN?

La alimentación contribuye al buen funcionamiento del sistema inmunológico. Mantener unos hábitos alimentarios correctos es fundamental para nuestra salud en general y más aún si vivimos con el VIH, que puede deteriorar el sistema de defensa del organismo.

RECOMENDACIONES DIETÉTICAS [1]

Seguir un patrón de dieta mediterránea es muy recomendable ya que, además de proporcionarnos una dieta apetitosa y adaptada a nuestras costumbres, nos ayuda a mantenernos sanos y a prevenir el desarrollo de numerosas enfermedades crónicas típicas de nuestro tiempo (como la diabetes, la obesidad, enfermedades cardiovasculares o algunos tipos de cáncer).

- Procure tomar alimentos ricos en fibra, minerales y antioxidantes:

- Tome al menos tres piezas de fruta al día, preferentemente de temporada y un puñado de frutos secos crudos (nueces, almendras…) 3 a 7 veces por semana; evite los frutos secos procesados por que pueden perder sus propiedades y/o o les pueden añadir grasas no saludables.

- Incorpore diariamente a su dieta cereales y derivados preferentemente integrales (pan, arroz, pasta…).

- Coma legumbres entre dos y cuatro veces por semana.

- Tome verduras y hortalizas al menos dos veces al día. Una de las tomas que sean crudas (ensaladas generalmente).

• Use preferentemente aceite de oliva virgen tanto para cocinar como para condimentar ensaladas. Disminuya en su dieta el consumo de grasas animales (como mantequilla, tocino, bollería industrial, carnes grasas, embutidos, quesos, bacon, natas…).

• Coma más pescado que carne (a igualdad de peso tiene menos calorías que la carne y aportan grasas más saludables). Se recomiendan de dos a cuatro raciones de pescado a la semana.

• Reduzca las carnes rojas (como ternera, cerdo, caza) y embutidos. Tómelas como máximo 2 veces por semana. Dentro de las carnes elija las que menos grasa tengan, como la de ave sin piel o conejo (en total 2-3 veces semana).

© 2015, 2019 TransCelerate BioPharma

• Evite el consumo de alimentos elaborados con aceites vegetales hidrogenados o grasas vegetales (de palma o coco) y/o animales. Suelen estar presentes en margarinas comerciales y numerosos productos de bollería industrial como galletas, alimentos de comida rápida "comida basura", precocinados, aperitivos salados).

• Los huevos son un magnífico alimento. Tomar de tres a siete huevos enteros por semana no le acarreará ningún problema.

• Evite alimentos y bebidas a los que durante la fabricación y procesamiento se les ha añadido azúcares, especialmente refrescos.

• Se recomienda ingerir dos raciones diarias de lácteos (una ración equivale a un vaso de leche, dos yogures; 40-60 g de queso curado ó 80-125 g de queso fresco). En periodos de crecimiento y mujeres embarazadas, lactantes y menopáusicas es aconsejable tomar una ración más. Puede ser entera. No obstante, si está obeso o con sobrepeso, su colesterol es elevado o toma más de medio litro al día, se recomienda tomarla desnatada o semidesnatada. El consumo de yogures y/o leches fermentadas es una forma muy saludable de tomar lácteos.

• Utilice sal yodada en sus platos. Se recomienda echar poca sal a las comidas y evitar los productos ultraprocesados, enlatados, embutidos y precocinados por su alto contenido en sal. Para dar más sabor a sus platos, puede utilizar hierbas aromáticas tales como tomillo, perejil, orégano, estragón, pimentón, comino etc.

EJERCICIO FISICO [2]

Se recomienda la realización de ejercicio físico de forma regular, a ser posible diariamente, según las posibilidades y preferencias de cada persona, y siempre previa consulta con el médico es fundamental para mejorar el estado general.

El ejercicio continuado, adaptado a su condición física y edad, previene la aparición de enfermedades crónicas y mejora su salud mental.

REDUCIR EL SEDENTARISMO

- Reducir los periodos prolongados de inactividad de mas de 2 horas, realizando descansos activos con sesiones estiramientos o dando un breve paseo.
- Utilizar los sistemas públicos de transporte en vez del coche o utilizar transporte activo (andando, en bici, etc.).
- Limitar el tiempo de pantalla (televisión, tableta, teléfono u ordenador).
- Subir las escaleras, cortar el césped, las tareas domésticas, pasear al perro, bailar … o aficionarse a cualquier deporte, son algunas de las maneras de introducir el ejercicio en su vida cotidiana.

© 2015, 2019 TransCelerate BioPharma

Lo que produce mas beneficios en tu salud es practicar actividad física regularmente, es decir, distribuirla a lo largo de toda la semana. Por ejemplo, 30 minutos de actividad moderada cinco o mas veces a la semana. Cuantos más días, mejor.

Las recomendaciones de actividad física también se pueden alcanzar sumando periodos más cortos; estos periodos deben ser de al menos 10 minutos cada uno.

Si lo combina con ejercicios de fuerza el efecto es aún mejor para la salud (ver vídeos)

http://www.estilosdevidasaludable.mscbs.gob.es/actividadFisica/actividad/recomendaciones/videosEjercicios/adultos/home.htm

Se recomiendan además:

- Actividades para mejorar el equilibrio. Se recomienda iniciarlas poco a poco y que tengan una dificultad progresiva.
- Actividades de fortalecimiento muscular progresivas y adaptadas a la condición de la persona.

OTRAS RECOMENDACIONES DE ESTILO DE VIDA SALUDABLE

- NO TOMAR BEBIDAS ALCOHÓLICAS
- ABANDONAR EL HÁBITO TABAQUICO
- Evitar los alimentos ricos en “azúcares solubles“ o azúcares de absorción rápida. Es decir, los alimentos preparados con azúcar de mesa, mermeladas, miel, confituras, productos de pastelería, bollería, helados, chocolates,

BIBLIOGRAFIA

1. https://www.seen.es/ModulGEX/workspace/publico/modulos/web/docs/apartados/1070/140420_125519_1352296268.pdf
2. https://estilosdevidasaludable.sanidad.gob.es/actividadFisica/actividad/recomendaciones/videosEjercicios/adultos/home.htm

10.9. Appendix 9: Information about reasons for temporary or definitive discontinuation of the study drug

Hospital Universitario La Paz
Hospital de Cantoblanco
Hospital Carlos III
SaludMadrid
Comunidad de Madrid

Estudio METforAging

HOJA DE RECOMENDACIONES Y PRECAUCIONES PARA EL PACIENTE EN TRATAMIENTO CON METFORMINA (O PLACEBO).

Durante este estudio es posible que reciba tratamiento con metformina (o placebo).

La metformina es un tratamiento utilizado frecuentemente para el tratamiento de la Diabetes Mellitus tipo 2, generalmente bien tolerado, y sin efectos secundarios moderados/graves previsibles a las dosis utilizadas, y con los criterios de inclusión y exclusión establecidos durante el estudio.

No obstante, debe tener en cuenta ciertas precauciones mientras participa en el estudio:

- Si tiene algún evento importante de salud, debe comunicar al médico que le atienda que puede estar tomando metformina (o placebo).
- El efecto secundario más grave de la metformina es la acidosis láctica, que es una complicación muy rara pero grave, y que se produce con mayor frecuencia si existe un empeoramiento de la función renal. Como medida de precaución, debe suspender la medicación del estudio (metformina o placebo) en caso de:
    - Deshidratación (diarrea o vómitos intensos, fiebre o reducción de la ingesta de líquidos). Debe interrumpirla de forma temporal hasta contactar con el equipo médico que le realiza el seguimiento en el estudio, que le indicará como proceder.
    - Ingreso por cualquier trastorno agudo que implique un riesgo de alteración de la función renal, tales como deshidratación, infecciones, shock, insuficiencia cardiaca o respiratoria, o infarto de miocardio. Debe interrumpirla de forma temporal hasta contactar con el equipo médico que le realiza el seguimiento en el estudio, que le indicará como proceder.
    - Realización de pruebas radiológicas con contrastes yodados (TAC/scanner). Debe interrumpir la metformina en el momento de la realización de la prueba y reiniciarla 48 horas después. Si es posible, comunique con antelación al equipo médico que le realiza el seguimiento en el estudio si tiene prevista alguna cita con contrastes radiológicos yodados, que le indicará como proceder.
- La metformina tiene pocas interacciones con otros medicamentos. No obstante, si comienza cualquier otra medicación durante su participación en el estudio, debe comunicárselo al equipo médico que le realiza el seguimiento en el estudio tan pronto como pueda contactar (si no es posible, en la próxima visita). Esto es especialmente relevante con algunos medicamentos que pueden alterar la función renal,

© 2015, 2019 TransCelerate BioPharma

especialmente ciertos medicamentos de uso común como los medicamentos para control de la hipertensión arterial, o los antiinflamatorios no esteroideos (ej. ibuprofeno, diclofenaco, dexketoprofeno…) si se toman de forma continuada (el consumo puntual o menor de 48 horas no supone un problema).

Ante cualquier duda, debe consultar con el equipo investigador, que resolverá sus dudas y le explicará cómo proceder (teléfonos de consulta: 91 727-70-99 // 91-727-73-84).

10.10. Appendix 10: Country-specific Requirements

Not applicable

10.11. Appendix 11: Abbreviations

AE	Adverse Event
ALT	Alanine aminotransferase
AMPK	activation of AMP-activated kinase
ART	Antiretroviral therapy
AST	Aspartate aminotransferase
BMI	Body mass index
BUN	Blood urea nitrogen
CD	cluster of differentiation
CES-D	Center for Epidemiological Studies-Depression
CI	confidence interval
CKD	Chronic Kidney disease
CONSORT	Consolidated Standards of Reporting Trials
CRF	case report form
CRP	C-reactive protein
CSR	clinical study report
CT	Computerized tomography
CTFG	Clinical Trials Facilitation Group
CVD	cardiovascular disease
DHEA	dehydroepiandrosterone
DNA	deoxyribonucleic acid
E/D	Early Discontinuation
EAA	Epigenetic Age Acceleration
ECG	electrocardiogram
eCRF	electronic case report form
eGFR	Glomerular filtration rate
EOT	end of treatment
FDA	Food and Drug Administration
GCP	Good Clinical Practice
GDF15	Growth Differentiation Factor 15
GGT	Gamma-glutamyl transpeptidase
HBsAg	hepatitis B surface antigen
hCG	Serum human chorionic gonadotropin
HIV	human immunodeficiency virus
HOMA-IR	Homeostatic Model Assessment of Insulin Resistance
hsCRP	high-sensitivity C-reactive protein
IA	interim analysis
IB	investigator's brochure
ICF	informed consent form
IEC	Independent Ethics Committees
IGF-1	Insulin-like growth factor 1
IL	Interleukin
IMP	investigational medicinal products
IRB	Institutional Review Boards
IUD	intrauterine device
IUS	intrauterine system
MATE	multidrug and toxin extrusion
NT-proBNP	N-terminal pro b-type natriuretic peptide
OCT	organic cation transporters
PBMC	peripheral blood mononuclear cells

PLWH	people living with HIV
QD	quaque die, once a day
qPCR	Quantitative polymerase chain reaction
RBC	Red blood cell Count
rhGH	recombinant human growth hormone
RNA	ribonucleic acid
ROS	reactive oxygen species
RTE	Recent thymic emigrants
SAE	serious adverse event
SoA	Schedule of Activities
SPPB	short physical performance battery
TAME	Targeting Aging with Metformin
TEMRA	Terminally differentiated effector memory
TNF	Tumor necrosis factor
TRIIM	Thymus Regeneration, Immunorestoration, and Insulin Mitigation
ULN	upper limit of normal
WBC	White blood cell
WOCBP	Women of Childbearing Potential

10.12. Appendix 12: Protocol Amendment History

The Protocol Amendment Summary of Changes Table for the current amendment is located directly before the Table of Contents (TOC).

Amendment [amendment number]: 1

This amendment is considered to be substantial based on the criteria set forth in Article 10(a) of Directive 2001/20/EC of the European Parliament and the Council of the European Union.

Overall Rationale for the Amendment

**Change**	**Description of Change**	**Section # and Name**
[…] Besides, metformin modify cell metabolism suppressing HIV-1 replication in human CD4 T-cells and humanized mice. Notably, clinical data showed that individuals with both HIV-1 and type 2 diabetes mellitus comorbidity exhibited an average 1.33-fold lower HIV-1 viral load than patients with HIV without diabetes among the early cART-treated cohort (6months) […]	A rationale study has been added.	1.1. Synopsis<br>(page 7)
To evaluate the effect of metformin compared to placebo as assessed by the viral reservoir parameters´ changes at week 48.	A secondary objective has been added.	1.1. Synopsis. Table Objectives and Endpoints<br>(page 9)
Changes in integrated and total (Gag) HIV-DNA<br>Changes in CA US HIV-RNA<br>Changes in intact proviral DNA	Endpoints have been added.	1.2. Synopsis. Table Objectives and Endpoints<br>(page 9)
o Viral reservoir in PBMC and CD4 T-cell. Genomic DNA from frozen PBMCs and CD4 T-cells ($1x10^6$ cells and $5x10^6$ cells) would be performed using the QIAamp DNA Blood Kit Mini Kit (Qiagen, Germany).<br>• To measure total and integrated (Gag) HIV-1 DNA, nucleic acid would be quantified by nested real time PCR. All measurements were performed in triplicate. The first PCR would be performed by using primers to the human Alu and HIV-gag regions. The second PCR, real-time PCR, detects HIV-specific products by using primers to the R and U5 regions within the HIV long terminal repeat (LTR)	The methods to evaluate the viral reservoir have been added.	8.3. Other study laboratory assessments<br>(page 37)

and a specific fluorescent probe. In the case of integrated HIV DNA, the Alu primer serves as an anchor in the human genome and the gag primer serves as an anchor in the HIV genome. Binding sites for these two primers would be present in the DNA target template only when HIV has integrated into the human genome. Furthermore, when the primer binding sites are close enough and aligned correctly, the region between them can be amplified exponentially. Quantification would be achieved by comparing the resultant signals to those obtained with an integration standard: ACH2 cells carrying one copy of integrated HIV-DNA per cell (NIH AIDS Reagent Program). To measure the background signal or the signal expected from unintegrated DNA, a control reaction using only the gag primer would be included in the first PCR. The signals from Alu-gag and gag-only would be compared to make sure the Alu-gag signal is stronger, and thus that the sample is positive for integration. Then a correlation would be developed that relates the PCR signals to proviral level, which is used to quantify the level of integration in the samples. To normalize the number of cells per sample, we would use a specific couple of primers and a fluorescent probe in a real-time PCR for CD3 amplification.<br><br>• To measure CA US HIV-RNA, total RNA would be isolate from total CD4 + T-cells using RNeasy Mini Kit with on-column DNase treatment (Qiagen), according to the manufacturer´s instructions. The quality (260nm/280nm ratio) and quantity of RNA collected were evaluated by spectrophotometry on a Nanodrop instrument. Extracted cellular RNA would be treated with DNase (DNA-free kit; Thermo Fisher Scientific, Waltham, MA) to remove DNA that could interfere with quantitation and reverse transcribed using random primers and SuperScript III reverse transcriptase (Thermo Fisher Scientific). CA HIV-1 US RNA would be measured using seminested quantitative PCR (qPCR)-based assays. Retrotranscription would be performed with random hexamers as primers and SuperCript III (Invitrogen,		

© 2015, 2019 TransCelerate BioPharma

Waltham, MA) according to the manufacturer´s instructions. Quantification of LTR-Gag HIV-RNA would be performed by using specific external primers for the first amplification and internal primers for the second amplification; and a fluorescent probe. US LTR-gag HIV-RNA standards were generated from plasmids by in vitro transcription (MEGAscript™ T7 Transcription Kit, ThermoFisher). The number of copies of each transcript was normalized to the levels of the reference gene beta glucuronidase (GUSB). All measurements were performed in triplicate.<br>o To measure Intact proviral DNA assay (IPDA) genomic DNA would be extracted using the QIAamp DNA Mini Kit (Qiagen). DNA concentrations would be determined using a Nanodrop instrument. Quantification of intact, 5' deleted, and 3' deleted and/or hypermutated proviruses would be carried out using specific primer/probe combinations optimized for subtype B HIV-1 using the technique described by Bruner K et al (26) using droplet digital PCR. All measurements would be performed in triplicate.		
CD4 T-cell isolation from fresh PBMCs using the CD4 T cell isolation Kit, Human (Miltenyi, Alemania) by negative selection. Purity of CD4 T-cell isolation would be evaluate by flow cytometry. Samples would be stored at -80ºC.	The method to isolate CD4 T-cells has been added.	8.3. Other study laboratory assessments<br>(page 39)
Viral reservoir:<br>Genomic DNA and RNA from frozen PBMCs and CD4 T-cell would be isolated and quantified by qPCR and droplet-PCR.	The protocol-required test for viral reservoir has been added.	Table 3: Protocol-Required Laboratory Tests at IdiPaz laboratory<br>(page 52)
[…]epigenetic, telomere and viral reservoir	This information has been added to 10.6. Appendix 6: Genetic	10.6. Appendix 6: Genetics<br>(página 74)

11. References

1. Saisho Y. Metformin and Inflammation: Its Potential Beyond Glucose-lowering Effect. Endocrine, Metab Immune Disord Targets. 2015;15(3):196–205.
2. Song YM, Lee YH, Kim JW, Ham DS, Kang ES, Cha BS, et al. Metformin alleviates hepatosteatosis by restoring SIRT1-mediated autophagy induction via an AMP-activated protein kinase-independent pathway. Autophagy. 2015;11(1):46–59.
3. Moiseeva O, Deschênes-Simard X, St-Germain E, Igelmann S, Huot G, Cadar AE, et al. Metformin inhibits the senescence-associated secretory phenotype by interfering with IKK/NF-κB activation. Aging Cell. 2013;12(3):489–98.
4. Kooy A, De Jager J, Lehert P, Bets D, Wulffelé MG, Donker AJM, et al. Long-term effects of metformin on metabolism and microvascular and macrovascular disease in patients with type 2 diabetes mellitus. Arch Intern Med. 2009;169(6):616–25.
5. Wang CP, Lorenzo C, Habib SL, Jo B, Espinoza SE. Differential effects of metformin on age related comorbidities in older men with type 2 diabetes. J Diabetes Complications [Internet]. 2017;31(4):679–86. Available from: http://dx.doi.org/10.1016/j.jdiacomp.2017.01.013
6. Currie CJ, Poole CD, Gale EAM. The influence of glucose-lowering therapies on cancer risk in type 2 diabetes. Diabetologia. 2009;52(9):1766–77.
7. Check Hayden E. Anti-ageing pill pushed as bona fide drug. Nature. 2015;522(7556):265–6.
8. Justice JN, Niedernhofer L, Robbins PD, Aroda VR, Espeland MA, Kritchevsky SB, et al. Development of clinical trials to extend healthy lifespan. Cardiovasc Endocrinol Metab. 2018;7(4):80–3.
9. López-Otín C, Blasco MA, Partridge L, Serrano M, Kroemer G. The hallmarks of aging. Cell. 2013;153(6):1194.
10. Cabreiro F, Au C, Leung K-Y, Vergara-Irigaray N, Cochemé HM, Noori T, et al. Metformin Retards Aging in C. elegans by Altering Microbial Folate and Methionine Metabolism. Cell [Internet]. 2013 Mar 28 [cited 2021 Apr 28];153(1):228–39. Available from: http://dx.
11. Barzilai N, Crandall JP, Kritchevsky SB, Espeland MA. Metformin as a Tool to Target Aging. Cell Metab [Internet]. 2016;23(6):1060–5. Available from: http://dx.doi.org/10.1016/j.cmet.2016.05.011
12. Kulkarni AS, Brutsaert EF, Anghel V, Zhang K, Bloomgarden N, Pollak M, et al. Metformin regulates metabolic and nonmetabolic pathways in skeletal muscle and subcutaneous adipose tissues of older adults. Aging Cell. 2018;17(2):1–5.
13. Marshall SM. 60 Years of Metformin Use: a Glance At the Past and a Look To the Future. Diabetologia. 2017;60(9):1561–5.
14. He L, Sabet A, Djedjos S, Miller R, Sun X, Hussain MA, et al. Metformin and Insulin Suppress Hepatic Gluconeogenesis through Phosphorylation of CREB Binding Protein. Cell [Internet]. 2009;137(4):635–46. Available from: http://dx.doi.org/10.1016/j.cell.2009.03.016
15. Liu B, Fan Z, Edgerton SM, Yang X, Lind SE, Thor AD. Potent anti-proliferative effects of metformin on trastuzumab-resistant breast cancer cells via inhibition of ErbB2/IGF-1 receptor interactions. Cell Cycle. 2011;10(17):2959–66.

16. Kickstein E, Krauss S, Thornhill P, Rutschow D, Zeller R, Sharkey J, et al. Biguanide metformin acts on tau phosphorylation via mTOR/protein phosphatase 2A (PP2A) signaling. Proc Natl Acad Sci U S A. 2010;107(50):21830–5.
17. Bridges HR, Jones AJY, Pollak MN, Hirst J. Effects of metformin and other biguanides on oxidative phosphorylation in mitochondria. Biochem J. 2014;462(3):475–87.
18. Duca FA, Côté CD, Rasmussen BA, Zadeh-Tahmasebi M, Rutter GA, Filippi BM, et al. Metformin activates a duodenal Ampk-dependent pathway to lower hepatic glucose production in rats. Nat Med. 2015;21(5):506–11.
19. Algire C, Moiseeva O, Deschênes-Simard X, Amrein L, Petruccelli L, Birman E, et al. Metformin reduces endogenous reactive oxygen species and associated DNA damage. Cancer Prev Res. 2012;5(4):536–43.
20. Fahy GM, Brooke RT, Watson JP, Good Z, Vasanawala SS, Maecker H, et al. Reversal of epigenetic aging and immunosenescent trends in humans. Aging Cell. 2019;18(6):1–12.
21. Routy JP, Isnard S, Mehraj V, Ostrowski M, Chomont N, Ancuta P, et al. Effect of metformin on the size of the HIV reservoir in non-diabetic ART-treated individuals: Single-arm non-randomised Lilac pilot study protocol. BMJ Open. 2019;9(4):1–8.
22. Shikuma CM, Chew GM, Kohorn L, Souza SA, Chow D, Sahbandar IN, et al. Short Communication: Metformin Reduces CD4 T Cell Exhaustion in HIV-Infected Adults on Suppressive Antiretroviral Therapy. AIDS Res Hum Retroviruses. 2020;36(4):303–5.
23. Kirkwood TBL. Understanding the odd science of aging. Cell. 2005;120(4):437–47.
24. Goldberger AL, Peng CK, Lipsitz LA. What is physiologic complexity and how does it change with aging and disease? Neurobiol Aging. 2002;23(1):23–6.
25. Watts J. Report urges swift action on global ageing “crisis”. Lancet. 2001;358(9283):731.
26. McLean AJ, Le Couteur DG. Aging Biology and Geriatric Clinical Pharmacology. Pharmacol Rev [Internet]. 2004 Jun;56(2):163–84. Available from: http://pharmrev.aspetjournals.org/lookup/doi/10.1124/pr.56.2.4
27. Appay V, Kelleher AD. Immune activation and immune aging in HIV infection. Curr Opin HIV AIDS. 2016;11(2):242–9.
28. Julia L. Marcus, Wendy Leyden, Alexandra N. Anderson, Rulin Hechter, Michael A. Horberg, Haihong Hu, Jennifer O. Lam, William J. Towner, Qing Yuan MJS. Increased Overall Life Expectancy But Not Comorbidity- Free Years for People With Hiv. Conf Retrovir opportunistic Infect March 8 -11, 2020 Boston, Massachusetts Abstr #151. 2020;
29. Guaraldi G, Orlando G, Zona S, Menozzi M, Carli F, Garlassi E, et al. Premature age-related comorbidities among HIV-infected persons compared with the general population. Clin Infect Dis. 2011;53(11):1120–6.
30. Kaplan-Lewis E, Aberg JA, Lee M. Aging with HIV in the ART era. Semin Diagn Pathol [Internet]. 2017;34(4):384–97. Available from: http://dx.doi.org/10.1053/j.semdp.2017.04.002
31. Rodger AJ, Lodwick R, Schechter M, Deeks S, Amin J, Gilson R, et al. Mortality in well controlled HIV in the continuous antiretroviral therapy arms of the SMART and ESPRIT trials compared with the general population. Aids. 2013;27(6):973–9.
32. Bürkle A, Moreno-Villanueva M, Bernhard J, Blasco M, Zondag G, Hoeijmakers JHJ, et al. MARK-AGE biomarkers of ageing. Mech Ageing Dev [Internet]. 2015;151:2–12. Available from: http://dx.doi.org/10.1016/j.mad.2015.03.006
33. Horvath S, Raj K. DNA methylation-based biomarkers and the epigenetic clock theory of ageing. Nat Rev Genet. 2018;19(6):371–84.

34. Levine ME, Lu AT, Quach A, Chen BH, Assimes TL, Bandinelli S, et al. An epigenetic biomarker of aging for lifespan and healthspan. Aging (Albany NY). 2018;10(4):573–91.
35. Lu AT, Quach A, Wilson JG, Reiner AP, Aviv A, Raj K, et al. DNA methylation GrimAge strongly predicts lifespan and healthspan. Aging (Albany NY). 2019 Jan 21;11(2):303–27.
36. Clinicaltrials.gov (senolytic drugs). https://clinicaltrials.gov/ct2/results?recrs=&cond=SENOLYTIC&term=&cntry=&state=&city=&dist=.
37. Song IH, Zong J, Borland J, Jerva F, Wynne B, Zamek-Gliszczynski MJ, et al. The Effect of Dolutegravir on the Pharmacokinetics of Metformin in Healthy Subjects. JAIDS J Acquir Immune Defic Syndr. 2016 Aug 1;72(4):400–7.
38. Montejano R, Stella-Ascariz N, Monge S, Bernardino JI, Pérez-Valero I, Montes ML, et al. Impact of Antiretroviral Treatment Containing Tenofovir Difumarate on the Telomere Length of Aviremic HIV-Infected Patients. JAIDS J Acquir Immune Defic Syndr [Internet]. 2017 Sep;76(1):102–9. Available from: http://insights.ovid.com/crossref?an=00126334-201709010-00015
39. Justice JN, Ferrucci L, Newman AB, Aroda VR, Bahnson JL, Divers J, et al. A framework for selection of blood-based biomarkers for geroscience-guided clinical trials: report from the TAME Biomarkers Workgroup. GeroScience. 2018;40(5–6):419–36.
40. Fried LP, Tangen CM, Walston J, Newman AB, Hirsch C, Gottdiener J, et al. Frailty in Older Adults: Evidence for a Phenotype. Journals Gerontol Ser A Biol Sci Med Sci. 2001 Mar 1;56(3):M146–57.
41. Mohd Hairi F, Mackenbach JP, Andersen-Ranberg K, Avendano M. Does socio-economic status predict grip strength in older Europeans? Results from the SHARE study in non-institutionalised men and women aged 50+. J Epidemiol Community Heal. 2010 Sep 1;64(9):829–37.
42. Castell MV, Sánchez M, Julián R, Queipo R, Martín S, Otero Á. Frailty prevalence and slow walking speed in persons age 65 and older: Implications for primary care. BMC Fam Pract. 2013;14:1–10.
43. U.S. Department of Health and Human Services, National Institutes of Health, National Institute of Allergy and Infectious Diseases D of A. Division of AIDS (DAIDS) Table for Grading the Severity of Adult and Pediatric Adverse Events. Natl Inst Allergy Infect Dis. 2017;(August):1–21.
44. Rubenstein LZ, Harker JO, Salvà A, Guigoz Y, Vellas B. Screening for undernutrition in geriatric practice: developing the short-form mini-nutritional assessment (MNA-SF). J Gerontol A Biol Sci Med Sci. 2001 Jun;56(6):M366-72.
45. Rockwood K, Theou O. Using the Clinical Frailty Scale in Allocating Scarce Health Care Resources. Can Geriatr J. 2020 Sep 1;23(3):210-215.

© 2015, 2019 TransCelerate BioPharma
