# Supplementary material for: Metformin and epigenetic age in non-diabetic older people with HIV in Madrid (METFORAGING): a double-blind, randomised, placebo-controlled, pilot trial
Source: eClinicalMedicine. 2026 Apr 18;95:103874. doi: 10.1016/j.eclinm.2026.103874 (PMC13098334; doi:10.1016/j.eclinm.2026.103874)
Supplement: Translated abstract Spanish [file mmc3.pdf]

The following translations in Spanish were submitted by the authors and we reproduce them as supplied. They have not been peer reviewed. Our editorial processes have only been applied to the original abstract in English, which should serve as reference for this manuscript.

Resumen en español

Antecedentes La metformina se estudia de forma creciente como posible agente geroprotector en la población general. El objetivo de este estudio fue evaluar la eficacia y seguridad de la metformina para mejorar la edad epigenética en personas mayores con VIH, no diabéticas y clínicamente bien controladas.

Métodos METFORAGING fue un ensayo piloto unicéntrico, doble ciego, aleatorizado, de grupos paralelos y controlado con placebo. Se reclutaron participantes no diabéticos con VIH, de 50 años o más, con supresión virológica mantenida en tratamiento antirretroviral estable —con carga viral indetectable durante al menos 12 meses— y recuento de linfocitos T $CD4^+$ >500 células/µL, procedentes de la consulta de VIH del Hospital Universitario La Paz (Madrid, España). Los participantes fueron asignados aleatoriamente (1:1) a recibir 850 mg de metformina oral o placebo equivalente dos veces al día durante 96 semanas. Participantes, investigadores y evaluadores de resultados permanecieron ciegos respecto a la asignación del tratamiento. Las visitas del estudio se realizaron al inicio y en las semanas 4, 8, 24, 48, 72 y 96. La adherencia se evaluó en cada visita mediante recuento de comprimidos e informe del propio participante. Al inicio y en la semana 96, se obtuvieron muestras de sangre periférica para calcular la edad biológica mediante 11 biomarcadores epigenéticos: relojes epigenéticos de primera generación (reloj de Horvath y reloj de Hannum), de segunda generación (PhenoAge y GrimAge V2), relojes derivados de componentes principales (PC-Horvath, PC-Hannum, PC-PhenoAge y PC-GrimAge), un reloj de tercera generación (DunedinPACE) y el estimador de longitud telomérica en sangre basado en metilación del ADN (DNAmTL). El desenlace primario fue la diferencia ajustada entre grupos en el cambio de la aceleración de la edad epigenética (EAA), medida con el reloj PhenoAge en la semana 96 en la población por protocolo. Los análisis se estratificaron por edad, sexo, recuento basal de CD4, tabaquismo, tratamiento con estatinas y vía de transmisión del VIH. El ensayo está registrado en EudraCT con el número 2021-003299-15.

Hallazgos Entre el 2 de marzo y el 2 de octubre de 2022, se evaluaron 55 personas, de las cuales 40 fueron asignadas aleatoriamente a metformina (n=19) o placebo (n=21). El reclutamiento se cerró con 40 participantes debido a la lentitud en la inclusión, por debajo del objetivo pragmático de 60 previsto en el protocolo. La mediana de edad fue de 56,4 años (RIQ 53,0–60,8), 12 participantes (30%) eran mujeres y 35 (87,5%) se identificaban como de raza blanca. La adherencia media por recuento de comprimidos fue del 97,5% en ambos grupos. Treinta y cinco participantes (87,5%) completaron el tratamiento hasta la semana 96 (n=17 en el grupo metformina y n=18 en el grupo placebo; población por protocolo). En la semana 96, la diferencia ajustada entre grupos (metformina frente a placebo) para la EAA de PhenoAge fue de −1,02 años (IC del 95%: −5,30 a 3,26; p=0,627). Se registraron 48 acontecimientos adversos en 16 participantes (84%) del grupo metformina y 48 en 19 participantes (90%) del grupo placebo. En el grupo metformina no se atribuyó ningún acontecimiento adverso grave al fármaco, y no se produjeron fallecimientos ni hospitalizaciones.

Interpretación Aunque no se observó una diferencia estadísticamente significativa en el desenlace primario entre grupos, estos hallazgos preliminares avalan la factibilidad de los ensayos de gerociencia en esta población y justifican la realización de estudios de mayor tamaño y con potencia estadística adecuada para determinar si la metformina es capaz de modificar el envejecimiento biológico en personas con VIH.

Financiación Fondo de Investigaciones Sanitarias, Instituto de Salud Carlos III y Unión Europea.
Keywords HIV, ageing, metformin, epigenetic age
